# Supplementary material for: A comparison of the prevalence of and modifiable risk factors for cognitive impairment among community-dwelling Canadian seniors over two decades, 1991–2009
Source: PLoS One. 2020 Dec 16;15(12):e0242911. doi: 10.1371/journal.pone.0242911 (PMC7743951; doi:10.1371/journal.pone.0242911)
Supplement: S1 File — (PDF) [file pone.0242911.s001.pdf]

Canadian Community Health Survey (CCHS)

Annual Component - Public Use Microdata File, 2009-2010

Derived Variable (DV) Specifications

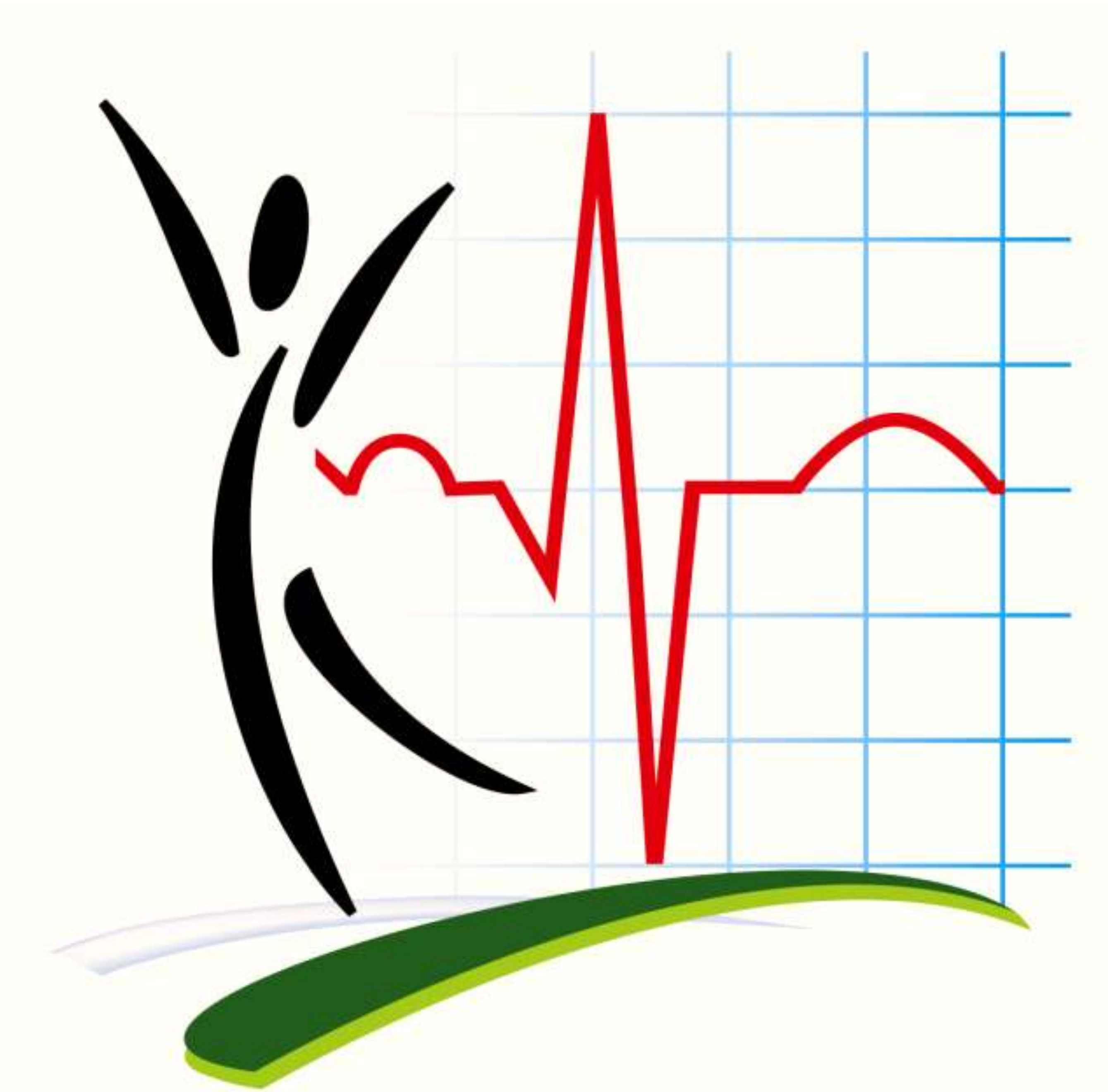

Statistics Canada Statistique Canada

Canada

Table of Contents

ADL Activities of Daily Living (1 DV)

1) ADLF6R - Need for help with instrumental activities of daily living 1

ALC Alcohol use (1 DV)

1) ALCDTTM - Type of Drinker (12 Months) 2

ALW Alcohol use during the past week (2 DVs)

1) ALWDWKY - Weekly Consumption 3
2) ALWDDLY - Average Daily Alcohol Consumption 3

CCC Chronic conditions (2 DVs)

1) CCCG102 - Diabetes - age first diagnosed - (G) 5
2) CCCDDIA - Diabetes type 5

CHP Contacts with health professionals (16 DVs)

1) CHPG02 - Number of nights as patient -(G) 7
2) CHPG04 - Number of consultations - fam. doctor/gen. practitioner -(G) 7
3) CHPG05 - Location of most recent contact - family doctor - (G) 7
4) CHPG07 - Number of consultations - eye specialist - (G) 8
5) CHPG09 - Number of consultations - other medical doctor - (G) 8
6) CHPG10 - Location of most recent contact - other medical doctor - (G) 9
7) CHPG12 - Number of consultations - nurse - (G) 9
8) CHPG13 - Location of most recent contact - nurse - (G) 10
9) CHPG15 - Number of consultations - dentist or orthodontist - (G) 10
10) CHPG17 - Number of consultations - chiropractor - (G) 11
11) CHPG19 - Number of consultations - physiotherapist - (G) 11
12) CHPG21 - Number of consultations - psychologist - (G) 11
13) CHPG23 - Number of consultations - social worker or counsellor - (G) 12
14) CHPG25 - No. of consultations - speech/audiology/occ. therapist - (G) 12
15) CHPGMDC - Number of Consultations with Medical Doctor/Paediatrician - Grouped 12
16) CHPFCOP - Consultations with Health Professionals 13

CPG Problem gambling (6 DVs)

1) CPGFGAM - Gambling Activity - Gambler vs. Non-gambler 14
2) CPGDSEV - Problem Gambling Severity Index (PGSI) - Modified Version 15
3) CPGDTYP - Type of Gambler 16
4) CPGDACT - Number of Types of Gambling Activities in the List Used to Calculate CPGI 17

5) CPGDINT - Gambling Interference - Mean 19

6) CPGFINT - Flag for Gambling Interference 20

DHH **Dwelling and household variables (7 DVs)**

1) DHHGAGE - Age - Grouped 21

2) DHHGMS - Marital status - Grouped 21

3) DHHGLE5 - Number of Persons in Household With Less Than 6 Years of Age - Grouped 22

4) DHHG611 - Number of Persons in Household between 6 to 11 Years of Age - Grouped 22

5) DHHGL12 - Number of Persons in Household With Less Than 12 Years of Age - Grouped 23

6) DHHGLVG - Living/Family Arrangement of Selected Respondent - Grouped 23

7) DHHGHSZ - Household size - Grouped 24

DIS **Distress (3 DVs)**

1) DISDK6 - Distress Scale - K6 27

2) DISDCHR - Chronicity of Distress and Impairment Scale 27

3) DISDDSX - Distress Scale - K10 28

DPS **Depression (4 DVs)**

1) DPSDSF - Derived Depression Scale - Short Form Score 31

2) DPSDPP - Depression Scale - Probability of Caseness to Respondents 32

3) DPSDWK - Number of Weeks Feeling Depressed - 12-Months 32

4) DPSDMT - Specific Month Last Felt Depressed 33

DRV **Driving and safety (1 DV)**

1) DRVFSBU - Passenger Seat Belt Use (Motor Vehicle) 34

EDU **Education (2 DVs)**

1) EDUDH04 - Highest Level of Education - Household, 4 Levels 35

2) EDUDR04 - Highest Level of Education - Respondent, 4 Levels 35

ETS **Exposure to second-hand smoke (1 DV)**

1) ETSG11 - Number of people who smoke inside home - (G) 36

FDC **Food choices (3 DVs)**

1) FDCFAVD - Avoids Certain Foods for Certain Content Reasons 37

2) FDCFCAH - Chooses or Avoids Certain Foods Because of Certain Health Concerns 37

3) FDCFCHO - Chooses Certain Foods for Certain Content Reasons 38

FSC **Food security (3 DVs)**

1) FSCDHFS2 - Household Food Security Status - Modified version 42

2) FSCDAFS2 - Food Security - Adult Status 43
3) FSCDCFS2 - Food Security - Child Status 44

FVC **Fruit and vegetable consumption (8 DVs)**

1) FVCDJUI - Daily Consumption - Fruit Juice 46
2) FVCDFRU - Daily Consumption - Other Fruit 46
3) FVCDSAL - Daily Consumption - Green Salad 47
4) FVCDPOT - Daily Consumption - Potatoes 47
5) FVCDCAR - Daily Consumption - Carrots 48
6) FVCDVEG - Daily Consumption - Other Vegetables 48
7) FVCDTOT - Daily Consumption - Total Fruit and Vegetable 49
8) FVCGTOT - Grouping of Daily Consumption - Total Fruit and Vegetable 49

GEN **General health (3 DVs)**

1) GENDHDI - Perceived Health 51
2) GENDMHI - Perceived Mental Health 51
3) GENGSWL - Satisfaction with life in general - (G) 51

GEO **Geography variables (3 DVs)**

1) GEOGPRV - Province of residence of respondent - (G) 53
2) GEODPMF - Health region - Grouped 53
3) GEODBCHA - Health Authority - British Columbia 54

HUI **Health utilities index (8 DVs)**

1) HUIGVIS - Vision Trouble (Function Code) - Grouped 55
2) HUIGHER - Hearing Problems (Function Code) - Grouped 56
3) HUIGSPE - Speech Trouble (Function Code) - Grouped 57
4) HUIGMOB - Mobility Trouble (Function Code) - Grouped 58
5) HUIGDEX - Dexterity Trouble (Function Code) - Grouped 59
6) HUIDEMO - Emotion Health Status 60
7) HUIDCOG - Cognition Health Status 61
8) HUIDHSI - Health Utilities Index 62

HUP **Health utilities index - Pain and discomfort (1 DV)**

1) HUPDPAD - Pain Health Status 63

HWT **Height and weight - Self-reported (5 DVs)**

1) HWTGHTM - Height (Metres) - Self-Reported - Grouped 64
2) HWTGWTK - Weight (kilograms - grouped) 66

3) HWTGBMI - Body mass index - grouped 67
4) HWTGISW - BMI classification for adults aged 18 and over (self reported) - international standard - grouped 67
5) HWTDCOL - BMI classification for children aged 12 to 17 (self-reported) - Cole classification system 68

IDG **Illicit drug use (9 DVs)**

1) IDGFLCA - Cannabis Drug Use - Lifetime (Including "One Time Only" Use) 73
2) IDGFLCM - Cannabis Drug Use - Lifetime (Excluding "One Time Only" Use) 73
3) IDGFYCM - Cannabis Drug Use - 12 month (Excluding "One Time Only" Use) 73
4) IDGFLA - Any Illicit Drug Use - Lifetime (Including "One Time Only" Use of Cannabis) 74
5) IDGFLAC - Any Illicit Drug Use - Lifetime (Excluding "One Time Only" Use of Cannabis) 75
6) IDGFYA - Any Illicit Drug Use - 12-Month (Including "One Time Only" Use of Cannabis) 75
7) IDGFYAC - Any Illicit Drug Use - 12-Month (Excluding "One Time Only" Use of Cannabis) 76
8) IDGDINT - Illicit Drug Interference 12-Month - Mean 77
9) IDGFINT - Flag for Illicit Drug Interference - 12-Month 77

INC **Income (7 DVs)**

1) INCG2 - Total household income - main source - Grouped 82
2) INCG7 - Main source of personal income - Grouped 83
3) INCGHH - Total Household Income - All Sources - Grouped 83
4) INCGPER - Personal Income - All Sources - Grouped 84
5) INCDRCA - Distribution of household income - National level 84
6) INCDRPR - Distribution of household income - Provincial level 85
7) INCDRRS - Distribution of household income - Health region level 86

INJ **Injuries (11 DVs)**

1) INJG02 - Number of injuries in past 12 months 87
2) INJG05 - Most Serious Injury 87
3) INJG06 - Most Serious Injury - body part affected - Grouped 88
4) INJG08 - Most Serious Injury - Place of occurrence - Grouped 88
5) INJG092 - Most Serious Injury - Activity when injured - Grouped 89
6) INJG11A - Most Serious Injury - How fell - Grouped 89
7) INJG14C - Most Serious Injury - Treated in clinic - Grouped 90
8) INJG14J2 - Most Serious Injury - treated at physio, mass therapist, chiro, other - Grouped 90
9) INJG17 - Other injuries - number (G) 91
10) INJGCAU - Cause of Injury - Grouped 91
11) INJDSTT - Injury Status 92

INW **Workplace injury (1 DV)**

1) INWGSOC - Occupation group (SOC) where injury occurred - (G) 93

LBS **Labour force (5 DVs)**

1) LBSG31 - Employment status - 12 months - (G) 94
2) LBSGHPW - Total usual hours worked - current jobs - Grouped 94
3) LBSDPFT - Full-time/part-time working status (for total usual hours) 94
4) LBSDWSS - Working status last week 95
5) LBSGSOC - Occupation group - (G) 95

MAS **Mastery (1 DV)**

1) MASDM1 - Derived Mastery Scale 97

MEX **Maternal experiences - Breastfeeding (3 DVs)**

1) MEXDEBF2 - Length of exclusive breastfeeding 99
2) MEXFEB6 - Exclusively Breastfed for 6 months (or more) 100

MXS **Maternal experiences - Smoking during pregnancy (4 DVs)**

1) MXSG02 - No. of cigarettes daily - last pregnancy (daily smoker) 102
2) MXSG03 - No. of cigarettes daily - last pregnancy (occasional smoker) 102
3) MXSG05 - No. of cigarettes daily - while breastfeeding (daily smoker) 103
4) MXSG06 - No. of cigarettes daily - while breastfeeding (occasional smoker) 103

OH2 **Oral health 2 (2 DVs)**

1) OH2FLIM - Social Limitation Due to Oral Health Status 105
2) OH2FOFP - Oral and Facial Pain and Discomfort 105

PAC **Physical activities (10 DVs)**

1) PACG2G - Number of times / 3 months - ice hockey - (G) 107
2) PACDEE - Daily Energy Expenditure in Leisure Time Physical Activities 107
3) PACFLEI - Participant In Leisure Time Physical Activity 115
4) PACDFM - Average Monthly Frequency of Leisure Time Physical Activity Lasting Over 15 Minutes 115
5) PACDFR - Frequency of All Leisure Time Physical Activity Lasting Over 15 Minutes 119
6) PACFD - Participant In Daily Leisure Time Physical Activity Lasting Over 15 Minutes 119
7) PACDPAI - Leisure Time Physical Activity Index 120
8) PACDLTI - Transportation and Leisure Time Physical Activity Index 120
9) PACDTLE - Daily Energy Expenditure in Transportation and Leisure Time Physical Activities 121
10) PACFLTI - Participant In Transportation or Leisure Time Physical Activity 122

PAF **Physical activities - Facilities at work (1 DV)**

1) PAFFACC - Access to Physical Activity Facilities at Work 123

PWB **Psychological well-being (1 DV)**

1) PWBDPWB - Psychological Well-Being Manifestation Scale (WBMMS) 124

RAC **Restriction of activities (3 DVs)**

1) RACG5 - Cause of health problem 128

2) RACDIMP - Impact of Health Problems 128

3) RACDPAL - Participation and Activity Limitation 129

REP **Repetitive strain injuries (1 DV)**

1) REPG3 - Repetitive strain injury 130

SAC **Sedentary activities (6 DVs)**

1) SACG1 - Number of hours - on a computer - past 3 mo - (G) 131

2) SACG2 - Number of hours - playing video games - past 3 mo - (G) 131

3) SACG3 - Number of hours - watching tv/videos - past 3 mo - (G) 132

4) SACG4 - Number of hours - reading - past 3 mo - (G) 132

5) SACDTOT - Total Number of Hours Per Week Spent In Sedentary Activities 133

6) SACDTER - Total number of hours per week spent in sedentary activities (excluding reading) 134

SCH **Smoking - Stages of change (1 DV)**

1) SCHDSTG - Smoking Stages of Change (Current and Former Smokers) 136

SDC **Socio-demographic characteristics (6 DVs)**

1) SDCGCBG - Country of Birth - Grouped 138

2) SDCGLHM - Language(s) spoken at home - (D, G) - Grouped 138

3) SDCFIMM - Immigration flag 139

4) SDCGRES - Length of time in Canada since immigration - Grouped 139

5) SDCGLNG - Language(s) in which respondent can converse - Grouped 140

6) SDCGCGT - Culture / Race Flag - Grouped 140

SFE **Self-esteem (1 DV)**

1) SFEDE1 - Derived Self-Esteem Scale 142

SFR **Health status (SF-36) (10 DVs)**

1) SFRDPFS - Physical Functioning Scale 145

2) SFRDSFS - Social Functioning Scale 146

3) SFRDPRF - Role Functioning (Physical) Scale 146

4) SFRDMRF - Role Functioning (Mental) Scale 147

5) SFRDGMH - General Mental Health Scale 147

6) SFRDVTS - Vitality Scale 147
7) SFRDBPS - Bodily Pain Scale 148
8) SFRDGHP - General Health Perceptions Scale 148
9) SFRDPCS - Summary Measure of Physical Health 149
10) SFRDMCS - Summary Measure of Mental Health 150

SMK Smoking (3 DVs)

1) SMKDSTY - Type of Smoker 152
2) SMKGSTP - Number of Years Since Stopping Smoking Completely - Grouped 152
3) SMKDYCS - Number of Years Smoked Daily (Current Daily Smokers Only) 153

SSA Social support - Availability (4 DVs)

1) SSADTNG - Tangible Social Support - MOS Subscale 157
2) SSADAFF - Affection - MOS Subscale 157
3) SSADSOC - Positive Social Interaction - MOS Subscale 158
4) SSADEMO - Emotional or Informational Support - MOS Subscale 158

UPE Use of protective equipment (3 DVs)

1) UPEFILS - Wears Protective Equipment when In-Line Skating 160
2) UPEFSKB - Wears Protective Equipment when Skateboarding 160
3) UPEFSNB - Wears Protective Equipment when Snowboarding 161

WTM Waiting times (9 DVs)

1) WTMDSO - Number of Waiting Days to See a Medical Specialist - Seen Specialist 162
2) WTMDSN - Number of Waiting Days to See a Medical Specialist - Not Seen Specialist 162
3) WTMDSA - Number of Acceptable Waiting Days to See a Medical Specialist 163
4) WTMDCO - Number of Waiting Days to Receive Non-Emergency Surgery - Surgery Done 163
5) WTMDCN - Number of Waiting Days to Receive Non-Emergency Surgery - Surgery Not Done 164
6) WTMDCA - Number of Acceptable Waiting Days to Receive Non-Emergency Surgery 164
7) WTMDTO - Number of Waiting Days for Diagnostic Test - Test Done 165
8) WTMDTN - Number of Waiting Days for Diagnostic Test - Test Not Done 165
9) WTMDTA - Number of Acceptable Waiting Days for Diagnostic Test 166

Activities of Daily Living (1 DV)

1) Need for help with instrumental activities of daily living

Variable name: ADLF6R

Based on: ADL_01, ADL_02, ADL_03, ADL_04, ADL_05, ADL_06

Description: This variable classifies respondents according to their need for help (because of health reasons) with instrumental activities of daily living such as preparing meals, shopping for groceries or other necessities, doing everyday housework, doing heavy household chores (washing walls, yard work), and personal care (washing, dressing or eating), moving about inside the house or paying bills.

Note: Prior to 2009, ADLF6R was called RACF6R and was a part of the Restriction of Activities (RAC) module. In 2009, all of the questions associated with the derived variable RACF6R were moved into a new module called Activities of Daily Living (ADL).

RACFUR is modified from RACAF6 (CCHS Cycle 1.1) by adding RAC_6G. The series of tasks included was revised based on the Participation and Activity Limitation Survey. Hence, this derived variable has been modified to take into account the revised set of tasks and thus this DV is not entirely comparable to RACAF6.

The variable was also modified in 2007 as question RAC_6D was no longer asked.

Specifications

Value	Condition(s)	Description	Notes
1	ADL_01 = 1 or<br>ADL_02 = 1 or<br>ADL_03 = 1 or<br>ADL_04 = 1 or<br>ADL_05 = 1 or<br>ADL_06 = 1	Needs help with at least one task	
2	ADL_01 = 2 and<br>ADL_02 = 2 and<br>ADL_03 = 2 and<br>ADL_04 = 2 and<br>ADL_05 = 2 and<br>ADL_06 = 2	Does not need help	
9	(ADL_01 = DK, R, NS) or<br>(ADL_02 = DK, R, NS) or<br>(ADL_03 = DK, R, NS) or<br>(ADL_04 = DK, R, NS) or<br>(ADL_05 = DK, R, NS) or<br>(ADL_06 = DK, R, NS)	At least one required question was not answered (don't know, refusal, not stated)	NS

Alcohol use (1 DV)

1) Type of Drinker (12 Months)

Variable name: ALCDTTM

Based on: ALC_1, ALC_2

Description: This variable indicates the type of drinker the respondent is based on his/her drinking habits in the past 12 months.

Note: This derived variable was introduced in 2007. Some of the questions contained within the Alcohol Use module in previous cycles moved to the Alcohol Use During the Past Week (ALW) and Alcohol Use - Former Drinkers (ALN) modules. As the new modules are optional content, most of the derived variables that were formerly calculated for all respondents in the Alcohol Use (ALC) module are now found in ALW and ALN and are only calculated for the health regions that selected these modules. ALCDTTM was created to allow the classification of all respondents according to their drinking habits in the past 12 months.

Specifications

Value	Condition(s)	Description	Notes
9	(ALC_1 in (97,98,99)) or<br>(ALC_2 in (97,98,99))	At least one required question was not answered (don't know, refusal, not stated)	NS
1	(2 <= ALC_2 < 96)	Regular drinker	
2	ALC_2 = 1	Occasional drinker	
3	ALC_1 = 2	Did not drink in the last 12 months	

Alcohol use during the past week (2 DVs)

1) Weekly Consumption

Variable name: ALWDWKY

Based on: ALC_1, ALW_1, ALW_2A1, ALW_2A2, ALW_2A3, ALW_2A4, ALW_2A5, ALW_2A6, ALW_2A7

Description: This variable indicates the total number of drinks consumed in the week prior to the interview.

Note: Respondents who did not have at least one drink in the past 12 months were excluded from the population. Before 2007, this derived variable was called ALCnDWKY. It was included in the Derived Variable Specifications for the Alcohol Use (ALC) module and was calculated for all respondents. It is now only calculated for respondents residing the health regions that selected the Alcohol Use During the Past Week (ALW) module.

Specifications

Value	Condition(s)	Description	Notes
996	DOALW = 2	Module not selected	NA
996	ALC_1 = 2	Population exclusions	NA
0	ALW_1 = 2	Has not had a drink in past week	
999	(ALW_1 = DK, R, NS) or (ALW_2A1 = DK, R, NS) or (ALW_2A2 = DK, R, NS) or (ALW_2A3 = DK, R, NS) or (ALW_2A4 = DK, R, NS) or (ALW_2A5 = DK, R, NS) or (ALW_2A6 = DK, R, NS) or (ALW_2A7 = DK, R, NS)	At least one required question was not answered (don't know, refusal, not stated)	NS
ALW_2A1 + ALW_2A2 + ALW_2A3 + ALW_2A4 + ALW_2A5 + ALW_2A6 + ALW_2A7	(0 <= ALW_2A1 < 100) and (0 <= ALW_2A2 < 100) and (0 <= ALW_2A3 < 100) and (0 <= ALW_2A4 < 100) and (0 <= ALW_2A5 < 100) and (0 <= ALW_2A6 < 100) and (0 <= ALW_2A7 < 100)	Number of drinks consumed in past week	(min: 0; max: 693)

2) Average Daily Alcohol Consumption

Variable name: ALWDDLY

Based on: ALWDWKY

Description: This variable indicates the average number of drinks the respondent consumed per day in the week prior to the interview.

Note: Respondents who did not have at least one drink in the last 12 months were excluded from the population. Before 2007, this derived variable was called ALCnDDLY. It was included in the Derived Variable Specifications for the Alcohol Use (ALC) module and was calculated for all respondents. It is now only calculated for respondents residing the health regions that selected the Alcohol Use During the Past Week (ALW) module.

Specifications

Value	Condition(s)	Description	Notes
996	DOALW = 2	Module not selected	NA
996	ALWDWKY = NA	Population exclusions	NA
999	ALWDWKY = NS	At least one required question was not answered (don't know, refusal, not stated)	NS

ALWDWKY / 7	ALWDWKY < 694	Average daily alcohol consumption	(Rounded to integer)<br>(min: 0; max: 99)

Chronic conditions (2 DVs)

1) Diabetes - age first diagnosed - (G)

Variable name: CCCG102

Based on: CCC_Q102

Description: The respondent's age when first diagnosed with diabetes.

Specifications

Value	Condition(s)	Description	Notes
96	CCC_Q102 = 96	Population exclusions	NA
99	CCC_Q102 = 99	At least one required question was not answered (don't know, refusal, not stated)	NS
1	CCC_Q102=<11	The respondent's age when first diagnosed with diabetes.	
2	12=<CCC_Q102=<17	The respondent's age when first diagnosed with diabetes.	
3	18=<CCC_Q102=<24	The respondent's age when first diagnosed with diabetes.	
4	25=<CCC_Q102=<29	The respondent's age when first diagnosed with diabetes.	
5	30=<CCC_Q102=<34	The respondent's age when first diagnosed with diabetes.	
6	35=<CCC_Q102=<39	The respondent's age when first diagnosed with diabetes.	
7	40=<CCC_Q102=<44	The respondent's age when first diagnosed with diabetes.	
8	45=<CCC_Q102=<49	The respondent's age when first diagnosed with diabetes.	
9	50=<CCC_Q102=<54	The respondent's age when first diagnosed with diabetes.	
10	55=<CCC_Q102=<59	The respondent's age when first diagnosed with diabetes.	
11	60=<CCC_Q102=<64	The respondent's age when first diagnosed with diabetes.	
12	65=<CCC_Q102=<69	The respondent's age when first diagnosed with diabetes.	
13	70=<CCC_Q102=<74	The respondent's age when first diagnosed with diabetes.	
14	75=<CCC_Q102=<79	The respondent's age when first diagnosed with diabetes.	
15	80=<CCC_Q102	The respondent's age when first diagnosed with diabetes.	

2) Diabetes type

Variable name: CCCDDIA

Based on: CCC_10A, CCC_10B, CCC_10C, CCC_101, CCC_102, CCC_105, CCC_106, DHH_AGE, DHH_SEX

Description: This is variable classifies diabetes as Type 1, Type 2, or Gestational, using the Ng-Dasgupta-Johnson algorithm (Health Reports, 19(1), March 2008).

Note: This derived variable was introduced in 2009.

Specifications

Value	Condition(s)	Description	Notes
6	CCC_101 > 1	Population exclusions	NA
9	(CCC_10A in (7,8,9)) or<br>(CCC_10B in (7,8,9)) or<br>(CCC_10C in (97,98,99)) or<br>(CCC_101 in (7,8,9)) or<br>(CCC_102 in (997,998,999)) or<br>(CCC_105 in (7,8,9)) or<br>(CCC_106 in (7,8,9))	At least one required question was not answered (don’t know, refusal, not stated)	NS
1	((DHH_SEX = 1) and<br>(CCC_101 = 1) and<br>(CCC_105 = 1) and<br>(CCC_106 = 2) and<br>((CCC_10C <=3) and ((DHH_AGE < 30) or<br>(CCC_102 < 30)))) or<br>((DHH_sex = 2) and<br>(CCC_101 = 1) and<br>(CCC_10B in (1,6)) and<br>(CCC_105 = 1) and<br>(CCC_106 = 2) and<br>((CCC_10C <=3) and ((DHH_AGE < 30) or<br>CCC_102 < 30))))	Type 1 diabetes	
2	CCC_101 = 1 and<br>((CCC_102 >=30) or<br>((CCC_102 <30) and<br>(CCC_106 =1) and<br>(CCC_10C >3)) or<br>((CCC_102 < 30) and<br>(CCC_106=1) and<br>(CCC_105 =1) and<br>(CCC_10C < 3))	Type 2 diabetes	
3	CCC_101 = 1 and<br>DHH_SEX = 2 and<br>CCC_10A = 1 and<br>CCC_10B = 2	Gestational diabetes	
4	Else	Unable to classify	

Contacts with health professionals (16 DVs)

1) Number of nights as patient -(G)

Variable name: CHPG02

Based on: CHP_02

Description: The number of nights as patient.

Note: In processing, if a respondent answered CHP_01 = 2 (no), the variable CHP_02 is given the value of 0. The "not stated" category includes respondents who reported in CHP module not having been a patient overnight in a hospital, nursing home or convalescent home in the past 12 months and who reported in INJ having been admitted to a hospital for one night following the injury that occurred in the past 12 months. // Prior to 2009, CHPG02 was called HCUG01A and was calculated with questions from the Health care utilization (HCU) module. In 2009, the HCU module was split and all questions associated with the derived variable CHPG02 were moved into a new module called Contacts with Health Professionals (CHP)

Specifications

Value	Condition(s)	Description	Notes
CHP_02	CHP_02 >= 1 and<br>CHP_02 <= 30	CHP_02	
31	CHP_02 > 30	31 or more	

2) Number of consultations - fam. doctor/gen. practitioner -(G)

Variable name: CHPG04

Based on: CHP_04

Description: This variable indicates the number of consultations with a family doctor/general practioner in the past 12 months.

Note: For respondents aged less than 18, includes consultations with pediatricians.

Specifications

Value	Condition(s)	Description	Notes
99	CHP_04 = DK, R, NS	At least one required question was not answered (don’t know, refusal, not stated)	NS
CHP_04	CHP_04	Number of consultations with a family doctor/general practioner	
31	31=<CHP_04	Number of consultations with a family doctor/general practioner	

3) Location of most recent contact - family doctor - (G)

Variable name: CHPG05

Based on: CHP_05

Description: This variable groups the location of the respondent's most recent contact with a family doctor.

Specifications

Value	Condition(s)	Description	Notes
96	CHP_05 = 96	Population exclusions	NA

99	CHP_05 = 99	At least one required question was not answered (don’t know, refusal, not stated)	NS
1	CHP_05 = 1	The most recent contact with a family doctor took place at the doctor's office.	
2	CHP_05 = 2	The most recent contact with a family doctor took place at a hospital emergency room.	
3	CHP_05 = 3	The most recent contact with a family doctor took place at a hospital outpatient clinic.	
4	CHP_05 = 4	The most recent contact with a family doctor took place at a walk-in clinic.	
5	CHP_05 = 5	The most recent contact with a family doctor took place at an appointment clinic.	
6	CHP_05 = 6	The most recent contact with a family doctor took place at a community health centre/CLSC.	
7	CHP_05 = 7, 8, ou 11	The most recent contact with a family doctor took place at work/at school/other.	
8	CHP_05 = 9	The most recent contact with a family doctor took place at home.	
9	CHP_05 = 10	The most recent contact with a family doctor took place as a telephone consultation only.	

4) Number of consultations - eye specialist - (G)

Variable name: CHPG07

Based on: CHP_07

Description: This variable indicates the number of consultations with an eye specialist in the past 12 months.

Specifications

Value	Condition(s)	Description	Notes
99	CHP_07 = DK, R, NS	At least one required question was not answered (don’t know, refusal, not stated)	NS
CHP_07	CHP_07	Number of consultations with an eye specialist	
12	12=<CHP_07	Number of consultations with an eye specialist	

5) Number of consultations - other medical doctor - (G)

Variable name: CHPG09

Based on: CHP_09

Description: This variable indicates the number of consultations with any other medical doctor (such as surgeon, allergist, orthopedist, gynaecologist, or psychiatrist) in the past 12 months.

Specifications

Value	Condition(s)	Description	Notes
99	CHP_09 = DK, R, NS	At least one required question was not answered (don’t know, refusal, not stated)	NS

CHP_09	CHP_09	Number of consultations with any other medical doctor (such as surgeon, allergist, orthopedist, gynaecologist, or psychiatrist)	
12	12=<CHP_09	Number of consultations with any other medical doctor (such as surgeon, allergist, orthopedist, gynaecologist, or psychiatrist)	

6) Location of most recent contact - other medical doctor - (G)

Variable name: CHPG10

Based on: CHP_10

Description: This variable groups the location of the respondent's most recent contact with - other medical doctor.

Specifications

Value	Condition(s)	Description	Notes
96	CHP_10 = 96	Population exclusions	NA
99	CHP_10 = 99	At least one required question was not answered (don't know, refusal, not stated)	NS
1	CHP_10 = 1	The most recent contact with the other medical doctor took place at the doctor's office.	
2	CHP_10 = 2	The most recent contact with the other medical doctor took place at a hospital emergency room.	
3	CHP_10 = 3	The most recent contact with the other medical doctor took place at a hospital outpatient clinic.	
4	CHP_10 = 4	The most recent contact with the other medical doctor took place at a walk-in clinic.	
5	CHP_10= 5	The most recent contact with the other medical doctor took place at an appointment clinic.	
6	CHP_10 = 6	The most recent contact with the other medical doctor took place at a community health centre/CLSC.	
7	CHP_10 = 7, 8, ou 11	The most recent contact with the other medical doctor took place at work/at school/other.	
8	CHP_10 = 9	The most recent contact with the other medical doctor took place at home.	
9	CHP_10 = 10	The most recent contact with the other medical doctor took place as a telephone consultation only.	

7) Number of consultations - nurse - (G)

Variable name: CHPG12

Based on: CHP_12

Description: This variable indicates the number of consultations with a nurse in the past 12 months.

Specifications

Value	Condition(s)	Description	Notes
99	CHP_12 = DK, R, NS	At least one required question was not answered	NS

		(don’t know, refusal, not stated)	
CHP_12	CHP_12	Number of consultations with a nurse	
12	12=<CHP_12	Number of consultations with a nurse	

8) Location of most recent contact - nurse - (G)

Variable name: CHPG13

Based on: CHP_13

Description: This variable groups the location of the respondent's most recent contact with a nurse.

Specifications

Value	Condition(s)	Description	Notes
96	CHP_13 = 96	Population exclusions	NA
99	CHP_13 = 99	At least one required question was not answered (don’t know, refusal, not stated)	NS
1	CHP_13 = 1	The most recent contact with a nurse took place at the doctor's office.	
2	CHP_13 = 2	The most recent contact with a nurse took place at a hospital emergency room.	
3	CHP_13 = 3	The most recent contact with a nurse took place at a hospital outpatient clinic.	
4	CHP_13 = 4	The most recent contact with a nurse took place at a walk-in clinic.	
5	CHP_13 = 5	The most recent contact with a nurse took place at an appointment clinic.	
6	CHP_13 = 6	The most recent contact with a nurse took place at a community health centre/CLSC.	
7	CHP_13 = 7, 8, ou 11	The most recent contact with a nurse took place at work/at school/other.	
8	CHP_13 = 9	The most recent contact with a nurse took place at home.	
9	CHP_13 = 10	The most recent contact with a nurse took place as a telephone consultation only.	

9) Number of consultations - dentist or orthodontist - (G)

Variable name: CHPG15

Based on: CHP_15

Description: This variable indicates the number of consultations with a dentist or orthodontist in the past 12 months.

Specifications

Value	Condition(s)	Description	Notes
99	CHP_15 = DK, R, NS	At least one required question was not answered (don’t know, refusal, not stated)	NS

CHP_15	CHP_15	Number of consultations with a dentist or orthodontist	
12	12=<CHP_15	Number of consultations with a dentist or orthodontist	

10) Number of consultations - chiropractor - (G)

Variable name: CHPG17

Based on: CHP_17

Description: This variable indicates the number of consultations with a chiropractor in the past 12 months.

Specifications

Value	Condition(s)	Description	Notes
99	CHP_17 = DK, R, NS	At least one required question was not answered (don't know, refusal, not stated)	NS
CHP_17	CHP_17	Number of consultations with a chiropractor	
31	31=<CHP_17	Number of consultations with a chiropractor	

11) Number of consultations - physiotherapist - (G)

Variable name: CHPG19

Based on: CHP_19

Description: This variable indicates the number of consultations with a physiotherapist in the past 12 months.

Specifications

Value	Condition(s)	Description	Notes
99	CHP_19 = DK, R, NS	At least one required question was not answered (don't know, refusal, not stated)	NS
CHP_19	CHP_19	Number of consultations with a physiotherapist	
31	31=<CHP_19	Number of consultations with a physiotherapist	

12) Number of consultations - psychologist - (G)

Variable name: CHPG21

Based on: CHP_21

Description: This variable indicates the number of consultations with a psychologist in the past 12 months.

Specifications

Value	Condition(s)	Description	Notes
99	CHP_21 = DK, R, NS	At least one required question was not answered (don't know, refusal, not stated)	NS
CHP_21	CHP_21	Number of consultations with a psychologist	

12	12=<CHP_21	Number of consultations with a psychologist	

13) Number of consultations - social worker or counsellor - (G)

Variable name: CHPG23

Based on: CHP_23

Description: This variable indicates the number of consultations with a social worker or counsellor in the past 12 months.

Specifications

Value	Condition(s)	Description	Notes
99	CHP_23 = DK, R, NS	At least one required question was not answered (don't know, refusal, not stated)	NS
CHP_23	CHP_23	Number of consultations with a social worker or counsellor	
12	12=<CHP_23	Number of consultations with a social worker or counsellor	

14) No. of consultations - speech/audiology/occ. therapist - (G)

Variable name: CHPG25

Based on: CHP_25

Description: This variable indicates the number of consultations with a speech, audiology or occupational therapist in the past 12 months.

Specifications

Value	Condition(s)	Description	Notes
99	CHP_25 = DK, R, NS	At least one required question was not answered (don't know, refusal, not stated)	NS
CHP_25	CHP_25	Number of consultations with a speech, audiology or occupational therapist	
12	12=<CHP_25	Number of consultations with a speech, audiology or occupational therapist	

15) Number of Consultations with Medical Doctor/Paediatrician - Grouped

Variable name: CHPGMDC

Based on: CHP_04, CHP_09

Description: This variable indicates the number of respondent's consultations, including over the phone, with medical doctor in the last 12 months.

Note: This variable has been grouped according to "less than 31 Consultations" and "31 or more".

Specifications

Value	Condition(s)	Description	Notes
999	(CHP_04 = DK, R, NS) or	At least one required question was not answered	NS

	(CHP_09 = DK, R, NS)	(don’t know, refusal, not stated)	
CHP_04 + CHP_09	(0 <= CHP_04 <= 366) and (0 <= CHP_09 <= 300)	Number of consultations with medical doctor. 31 or more consultations are grouped together.	(min: 0; max: 666)

16) Consultations with Health Professionals

Variable name: CHPFCOP

Based on: CHP_04, CHP_07, CHP_09, CHP_12, CHP_15, CHP_17, CHP_19, CHP_21, CHP_23, CHP_25

Description: This variable indicates whether respondents saw or talked to at least 1 health professional in the last 12 months.

Note: Prior to 2009, CHPFCOP was called HCUFCOP and was derived with questions from the Health care utilization (HCU) module. In 2009, the HCU module was split and all questions associated with the derived variable HCUFCOP were moved into a new module called Contacs with Health Professionals (CHP).

Specifications

Value	Condition(s)	Description	Notes
2	CHP_04 = 0 and<br>CHP_07 = 0 and<br>CHP_09 = 0 and<br>CHP_12 = 0 and<br>CHP_15 = 0 and<br>CHP_17 = 0 and<br>CHP_19 = 0 and<br>CHP_21 = 0 and<br>CHP_23 = 0 and<br>CHP_25 = 0	Did not consult a health professional last year	
1	(0 < CHP_04 < NA) or<br>(0 < CHP_07 < NA) or<br>(0 < CHP_09 < NA) or<br>(0 < CHP_12 < NA) or<br>(0 < CHP_15 < NA) or<br>(0 < CHP_17 < NA) or<br>(0 < CHP_19 < NA) or<br>(0 < CHP_21 < NA) or<br>(0 < CHP_23 < NA) or<br>(0 < CHP_25 < NA)	Consulted a health professional at least once last year	
9	(CHP_04 = DK, R, NS) or<br>(CHP_07 = DK, R, NS) or<br>(CHP_09 = DK, R, NS) or<br>(CHP_12 = DK, R, NS) or<br>(CHP_15 = DK, R, NS) or<br>(CHP_17 = DK, R, NS) or<br>(CHP_19 = DK, R, NS) or<br>(CHP_21 = DK, R, NS) or<br>(CHP_23 = DK, R, NS) or<br>(CHP_25 = DK, R, NS)	At least one required question was not answered (don’t know, refusal, not stated)	NS

Problem gambling (6 DVs)

This module assesses gambling activity and problems with gambling. The questionnaire and derived variables are based on the Canadian Problem Gambling Index (CPGI) but a number of modifications made both to the questionnaire and the calculation of the derived variables (described below) means that the results are not directly comparable to the CPGI.

Temporary Reformat

Value	Condition(s)	Description	Notes
**CPGT03**			
(CPG_03-1)	(CPG_03 = 1, 2, 3, 4)	Rescale the variables so that the range is from 0 to 3 instead of 1 to 4	
**CPGT04**			
(CPG_04-1)	(CPG_04 = 1, 2, 3, 4)	Rescale the variables so that the range is from 0 to 3 instead of 1 to 4	
**CPGT05**			
(CPG_05-1)	(CPG_05 = 1, 2, 3, 4)	Rescale the variables so that the range is from 0 to 3 instead of 1 to 4	
**CPGT06**			
(CPG_06-1)	(CPG_06 = 1, 2, 3, 4)	Rescale the variables so that the range is from 0 to 3 instead of 1 to 4	
**CPGT07**			
(CPG_07-1)	(CPG_07 = 1, 2, 3, 4)	Rescale the variables so that the range is from 0 to 3 instead of 1 to 4	
**CPGT08**			
(CPG_08-1)	(CPG_08 = 1, 2, 3, 4)	Rescale the variables so that the range is from 0 to 3 instead of 1 to 4	
**CPGT09**			
(CPG_09-1)	(CPG_09 = 1, 2, 3, 4)	Rescale the variables so that the range is from 0 to 3 instead of 1 to 4	
**CPGT10**			
(CPG_10-1)	(CPG_10 = 1, 2, 3, 4)	Rescale the variables so that the range is from 0 to 3 instead of 1 to 4	
**CPGT13**			
(CPG_13-1)	(CPG_13 = 1, 2, 3, 4)	Rescale the variables so that the range is from 0 to 3 instead of 1 to 4	

1) Gambling Activity - Gambler vs. Non-gambler

Variable name: CPGFGAM

Based on: CPG_01A, CPG_01B, CPG_01C, CPG_01D, CPG_01E, CPG_01F, CPG_01G, CPG_01H, CPG_01I, CPG_01J, CPG_01K, CPG_01L,CPG_01M

Description: This variable categorizes respondents as gamblers or non-gamblers. A non-gambler is defined as someone who has not engaged at all in the past year in any type of the gambling activities listed. A gambler is defined as someone who has engaged in at least one type of gambling activity in the past year.

Specifications

Value	Condition(s)	Description	Notes
6	DOCPG = 2	Module not selected	NA
9	ADM_PRX = 1	Module not asked - proxy interview	NS

1	(1 <= CPG_01A <= 7) or<br>(1 <= CPG_01B <= 7) or<br>(1 <= CPG_01C <= 7) or<br>(1 <= CPG_01D <= 7) or<br>(1 <= CPG_01E <= 7) or<br>(1<= CPG_01F <= 7) or<br>(1 <= CPG_01G <= 7) or<br>(1 <= CPG_01H <= 7) or<br>(1 <= CPG_01I <= 7) or<br>(1 <= CPG_01J <= 7) or<br>(1 <= CPG_01K <= 7) or<br>(1 <= CPG_01L <= 7) or<br>(1 <= CPG_01M <= 7)	Gambler	
2	CPG_01A = 8 and<br>CPG_01B = 8 and<br>CPG_01C = 8 and<br>CPG_01D = 8 and<br>CPG_01E = 8 and<br>CPG_01F = 8 and<br>CPG_01G = 8 and<br>CPG_01H = 8 and<br>CPG_01I = 8 and<br>CPG_01J = 8 and<br>CPG_01K = 8 and<br>CPG_01L = 8 and<br>CPG_01M = 8	Non-gambler	
9	(CPG_01A = DK, R, NS) or<br>(CPG_01B = DK, R, NS) or<br>(CPG_01C = DK, R, NS) or<br>(CPG_01D = DK, R, NS) or<br>(CPG_01E = DK, R, NS) or<br>(CPG_01F = DK, R, NS) or<br>(CPG_01G = DK, R, NS) or<br>(CPG_01H = DK, R, NS) or<br>(CPG_01I = DK, R, NS) or<br>(CPG_01J = DK, R, NS) or<br>(CPG_01K = DK, R, NS) or<br>(CPG_01L = DK, R, NS) or<br>(CPG_01M = DK, R, NS)	At least one required question was not answered (don’t know, refusal, not stated)	NS

2) Problem Gambling Severity Index (PGSI) - Modified Version

Variable name: CPGDSEV

Based on: CPG_02, CPG_03, CPG_04, CPG_05, CPG_06, CPG_07, CPG_08, CPG_09, CPG_10, CPG_13, CPGFGAM

Description: This variable indicates the level of gambling problems of respondents using a 9 item scale.

Note: A modification from the CPGI is that if respondents volunteered in CPGB_02 that "I am not a gambler", they were not asked the severity questions despite having reported gambling activity in the past 12 months. These respondents are assigned a code of 95 for this variable. In addition, respondents who reported participating in each gambling activity from CPGB_01B to CPGB_01M at most 1 to 5 times each during the past year were not asked questions on problem gambling. Finally, gambling activities were regrouped in the questionnaire into fewer categories than used in the original CPGI. Modifications made to the original instrument were approved by Dr. Wynne. Non-gamblers have been excluded from the population. Higher scores indicate more problems associated with gambling.

Specifications

Value	Condition(s)	Description	Notes
96	DOCPG = 2	Module not selected	NA
96	CPGFGAM = 2	Population exclusions	NA
99	ADM_PRX = 1	Module not asked - proxy interview	NS

99	CPGFGAM = NS or (CPGT03 = DK, R, NS) or (CPGT04 = DK, R, NS) or (CPGT05 = DK, R, NS) or (CPGT06 = DK, R, NS) or (CPGT07 = DK, R, NS) or (CPGT08 = DK, R, NS) or (CPGT09 = DK, R, NS) or (CPGT10 = DK, R, NS) or (CPGT13 = DK, R, NS)	At least one required question was not answered (don’t know, refusal, not stated)	NS
95	CPG_02 = 5	Does not consider himself a gambler - severity questions not asked	
0	CPGFGAM = 1 and CPG_02 = NA	Gambled at most 1-5 times a year for each gambling activity mentioned - severity questions not asked	
CPGT03 + CPGT04 + CPGT05 + CPGT06 + CPGT07 + CPGT08 + CPGT09 + CPGT10 + CPGT13	(CPGT03 = 0, 1, 2, 3) and (CPGT04 = 0, 1, 2, 3) and (CPGT05 = 0, 1, 2, 3) and (CPGT06 = 0, 1, 2, 3) and (CPGT07 = 0, 1, 2, 3) and (CPGT08 = 0, 1, 2, 3) and (CPGT09 = 0, 1, 2, 3) and (CPGT10 = 0, 1, 2, 3) and (CPGT13 = 0, 1, 2, 3)	Score obtained on the problem gambling severity index	(min: 0; max: 27)

Reference: Modified from the CPGI (Canadian Problem Gambling Index) developed by Harold Wynne and Jackie Ferris. "The Canadian Problem Gambling Index, Final Report." - Final Report, Submitted to the Canadian Centre on Substance Abuse. Jackie Ferris, Harold Wynne.

3) Type of Gambler

Variable name: CPGDTYP

Based on: CPGDSEV, CPGFGAM

Description: This variable categorizes respondents based on the severity of their problems associated with gambling.

Note: A modification from the CPGI is that if respondents volunteered in CPG_02 that "I am not a gambler", they were not asked the severity questions despite having reported gambling activity in the past 12 months. These respondents are assigned a code of 95. In addition, respondents who reported participating in each gambling activity from CPG_01 to CPG_01M at most 1 to 5 times each during the past year were not asked questions on problem gambling. Finally, gambling activities were regrouped in the questionnaire into fewer categories than used in the original CPGI. Modifications made to the original instrument were approved by Dr. Wynne.

Specifications

Value	Condition(s)	Description	Notes
96	DOCPG = 2	Module not selected	NA
99	ADM_PRX = 1	Module not asked (proxy interview)	NS
99	CPGDSEV = NS	At least one required question was not answered (don't know, refusal, not stated)	NS
95	CPGDSEV = 95	Does not consider himself a gambler - severity questions not asked	
1	CPGFGAM = 2	Non-gambler	
2	CPGDSEV = 0	Non-problem gambler	
3	(CPGDSEV = 1, 2)	Low risk gambler	
4	(CPGDSEV = 3, 4, 5, 6, 7)	Moderate risk gambler	
5	CPGDSEV >= 8	Problem gambler	

Reference: Modified from the CPGI (Canadian Problem Gambling Index) developed by Harold Wynne and Jackie Ferris. "The Canadian Problem Gambling Index, Final Report." - Final Report, Submitted to the Canadian Centre on Substance Abuse. Jackie Ferris, Harold Wynne.

4) Number of Types of Gambling Activities in the List Used to Calculate CPGI

Variable name: CPGDACT

Based on: CPG_01A, CPG_01B, CPG_01C, CPG_01D, CPG_01E, CPG_01F, CPG_01G, CPG_01H, CPG_01I, CPG_01J, CPG_01K, CPG_01L, CPG_01M

Description: This variable indicates the number of different types of gambling activities, in the list of gambling activities used to calculate CPGI, in which the respondent participated.

Temporary Reformat

Value	Condition(s)	Description	Notes
**CPGT01A**			
0	CPG_01A = 8	Temporarily recode 8 to 0 so that "never" does not count in sum of different types of gambling activity participated in.	
1	(1<= CPG_01A <=7)	Temporarily recode 1 to 7 to 1 so that each activity can be counted as a different type of gambling activity participated in.	
**CPGT01B**			
0	CPG_01B = 8	Temporarily recode 8 to 0 so that "never" does not count in sum of different types of gambling activity participated in.	
1	(1<= CPG_01B <=7)	Temporarily recode 1 to 7 to 1 so that each activity can be counted as a different type of gambling activity participated in.	
**CPGT01C**			
0	CPG_01C = 8	Temporarily recode 8 to 0 so that "never" does not count in sum of different types of gambling activity participated in.	
1	(1<= CPG_01C <=7)	Temporarily recode 1 to 7 to 1 so that each activity can be counted as a different type of gambling activity participated in.	
**CPGT01D**			
0	CPG_01D = 8	Temporarily recode 8 to 0 so that "never" does not count in sum of different types of gambling activity participated in.	
1	(1<= CPG_01D <=7)	Temporarily recode 1 to 7 to 1 so that each activity can be counted as a different type of gambling activity participated in.	
**CPGT01E**			
0	CPG_01E = 8	Temporarily recode 8 to 0 so that "never" does not count in sum of different types of gambling activity participated in.	
1	(1<= CPG_01E <=7)	Temporarily recode 1 to 7 to 1 so that each activity can be counted as a different type of gambling activity participated in.	
**CPGT01F**			
0	CPG_01F = 8	Temporarily recode 8 to 0 so that "never" does not count in sum of different types of gambling activity participated in.	

1	(1<= CPG_01F <=7)	Temporarily recode 1 to 7 to 1 so that each activity can be counted as a different type of gambling activity participated in.	
**CPGT01G**			
0	CPG_01G = 8	Temporarily recode 8 to 0 so that "never" does not count in sum of different types of gambling activity participated in.	
1	(1<= CPG_01G <=7)	Temporarily recode 1 to 7 to 1 so that each activity can be counted as a different type of gambling activity participated in.	
**CPGT01H**			
0	CPG_01H = 8	Temporarily recode 8 to 0 so that "never" does not count in sum of different types of gambling activity participated in.	
1	(1<= CPG_01H <=7)	Temporarily recode 1 to 7 to 1 so that each activity can be counted as a different type of gambling activity participated in.	
**CPGT01I**			
0	CPG_01I = 8	Temporarily recode 8 to 0 so that "never" does not count in sum of different types of gambling activity participated in.	
1	(1<= CPG_01I <=7)	Temporarily recode 1 to 7 to 1 so that each activity can be counted as a different type of gambling activity participated in.	
**CPGT01J**			
0	CPG_01J = 8	Temporarily recode 8 to 0 so that "never" does not count in sum of different types of gambling activity participated in.	
1	(1<= CPG_01J <=7)	Temporarily recode 1 to 7 to 1 so that each activity can be counted as a different type of gambling activity participated in.	
**CPGT01K**			
0	CPG_01K = 8	Temporarily recode 8 to 0 so that "never" does not count in sum of different types of gambling activity participated in.	
1	(1<= CPG_01K <=7)	Temporarily recode 1 to 7 to 1 so that each activity can be counted as a different type of gambling activity participated in.	
**CPGT01L**			
0	CPG_01L = 8	Temporarily recode 8 to 0 so that "never" does not count in sum of different types of gambling activity participated in.	
1	(1<= CPG_01L <=7)	Temporarily recode 1 to 7 to 1 so that each activity can be counted as a different type of gambling activity participated in.	
**CPGT01M**			
0	CPG_01M = 8	Temporarily recode 8 to 0 so that "never" does not count in sum of different types of gambling activity participated in.	
1	(1<= CPG_01M <=7)	Temporarily recode 1 to 7 to 1 so that each activity can be counted as a different type of gambling activity participated in.	

Specifications

Value	Condition(s)	Description	Notes

96	DOCPG = 2	Module not selected	NA
99	ADM_PRX = 1	Module not asked - proxy interview	NS
99	(CPGT01A = DK, R, NS) or<br>(CPGT01B = DK, R, NS) or<br>(CPGT01C = DK, R, NS) or<br>(CPGT01D = DK, R, NS) or<br>(CPGT01E = DK, R, NS) or<br>(CPGT01F = DK, R, NS) or<br>(CPGT01G = DK, R, NS) or<br>(CPGT01H = DK, R, NS) or<br>(CPGT01I = DK, R, NS) or<br>(CPGT01J = DK, R, NS) or<br>(CPGT01K = DK, R, NS) or<br>(CPGT01L = DK, R, NS) or<br>(CPGT01M = DK, R, NS)	At least one required question was not answered (don't know, refusal, not stated)	NS
CPGT01A +<br>CPGT01B +<br>CPGT01C +<br>CPGT01D +<br>CPGT01E +<br>CPGT01F +<br>CPGT01G +<br>CPGT01H +<br>CPGT01I +<br>CPGT01J +<br>CPGT01K +<br>CPGT01L +<br>CPGT01M	(CPGT01A = 0, 1) and<br>(CPGT01B = 0, 1) and<br>(CPGT01C = 0, 1) and<br>(CPGT01D = 0, 1) and<br>(CPGT01E = 0, 1) and<br>(CPGT01F = 0, 1) and<br>(CPGT01G = 0, 1) and<br>(CPGT01H = 0, 1) and<br>(CPGT01I = 0, 1) and<br>(CPGT01J = 0, 1) and<br>(CPGT01K = 0, 1) and<br>(CPGT01L = 0, 1) and<br>(CPGT01M = 0, 1)	Number of different types of gambling activities participated in, in the list used to calculate CPGI, during the previous 12 months	(min: 0; max: 13)

5) Gambling Interference - Mean

Variable name: CPGDINT

Based on: CPG_19A, CPG_9B1, CPG_9B2, CPG_19C, CPG_19D

Description: This variable indicates the interference that gambling had on daily activities and responsibilities in the past 12 months. This is a mean of the 5 items.

Note: Respondents who did not gamble enough or did not indicate problems with gambling were excluded from the population. Higher scores indicate greater interference.

Specifications

Value	Condition(s)	Description	Notes
99.6	DOCPG = 2	Module not selected	NA
99.6	CPG_19A = NA	Population exclusions	NA
99.9	ADM_PRX = 1	Module not asked - proxy interview	NS
99.9	(CPG_19A = DK, R, NS) or<br>(CPG_9B1 = DK, R, NS) or<br>(CPG_9B21 = DK, R, NS) or<br>(CPG_19C = DK, R, NS) or<br>(CPG_19D = DK, R, NS)	At least one required question was not answered (don't know, refusal, not stated)	NS
((CPG_19A +<br>CPG_9B1 +<br>CPG_91B2 +<br>CPG_19C +<br>CPG_19D) / 5	(0 <= CPG_9B1 <= 10) and<br>(0 <= CPG_9B2 <= 10) and<br>(0 <= CPG_19A <= 10) and<br>(0 <= CPG_19C <= 10) and<br>(0 <= CPG_19D <= 10)	Degree of gambling interference = mean of all 5 items<br>(mean value based on all 5 questions)	(Rounded to one decimal place)<br>(min: 0; max: 10.0)

(CPG_19A + CPG_9B2 + CPG_19C + CPG_19D) / 4	CPG_9B1 = 11 and (0 <= CPG_9B2 <= 10) and (0 <= CPG_19A <= 10) and (0 <= CPG_19C <= 10) and (0 <= CPG_19D <= 10)	Degree of gambling interference (mean value based on 4 questions) Interference = mean of 4 items that applied CPG_9B1 (ability to attend school was not applicable)	(Rounded to one decimal place) (min: 0; max: 10.0)
(CPG_19A + CPG_9B1 + CPG_19C + CPG_19D) / 4	(0 <= CPG_9B1 <= 10) and CPG_9B2 = 11 and (0 <= CPG_19A <= 10) and (0 <= CPG_19C <= 10) and (0 <= CPG_19D <= 10)	Degree of gambling interference (mean value based on 4 questions) Interference = mean of 4 items that applied CPG_9B2 (ability to work at a job was not applicable)	(Rounded to one decimal place) (min: 0; max: 10.0)
(CPG_19A + CPG_19C + CPG_19D) / 3	CPG_9B1 = 11 and CPG_9B2 = 11 and (0 <= CPG_19A <= 10) and (0 <= CPG_19C <= 10) and (0 <= CPG_19D <= 10)	Degree of gambling interference (mean value based on 3 questions) Interference = mean of 3 items that applied CPG_9B1 and CPG_9B2 were not applicable	(Rounded to one decimal place) (min: 0; max: 10.0)

6) Flag for Gambling Interference

Variable name: CPGFINT

Based on: CPG_19A, CPG_9B1, CPG_9B2, CPG_19C, CPG_19D

Description: This variable indicates the interference that gambling had on daily activities and responsibilities in the past 12 months. This is a threshold that indicates whether gambling interferes significantly with the person’s normal routine, occupational (academic) functioning, or social activities or relationships.

Note: Respondents who did not gamble enough or did not indicate problems with gambling were excluded from the population.

Specifications

Value	Condition(s)	Description	Notes
6	DOCPG = 2	Module not selected	NA
6	CPG_19A = NA	Population exclusions	NA
9	ADM_PRX = 1	Module not asked - proxy interview	NS
1	(4 <= CPG_19A <= 10) or (4 <= CPG_9B1 <= 10) or (4 <= CPG_9B2 <= 10) or (4 <= CPG_19C <= 10) or (4 <= CPG_19D <= 10)	Gambling interfered significantly with the normal routine, occupational (academic) functioning, or social activities or relationships in the past 12 months	
2	(0 <= CPG_19A <= 3) and [(0 <= CPG_9B1 <= 3) or CPG_9B1 = 11] and [(0 <= CPG_9B2 <= 3) or CPG_9B2 = 11] and (0 <= CPG_19C <= 3) and (0 <= CPG_19D <= 3)	Gambling did not interfere significantly with the normal routine, occupation (academic) functioning or social activities or relationships in the past 12 months	
9	(CPG_19A = DK, R, NS) or (CPG_9B1 = DK, R, NS) or (CPG_9B2 = DK, R, NS) or (CPG_19C = DK, R, NS) or (CPG_19D = DK, R, NS)	At least one required question was not answered (don’t know, refusal, not stated)	NS

Dwelling and household variables (7 DVs)

1) Age - Grouped

Variable name: DHHGAGE

Based on: DHH_AGE

Description: This variable indicates the age of the selected respondent.

Specifications

Value	Condition(s)	Description	Notes
1	12 <= DHH_AGE <= 14	Age between 12 and 14	
2	15 <= DHH_AGE <= 17	Age between 15 and 17	
3	18 <= DHH_AGE <= 19	Age between 18 and 19	
4	20 <= DHH_AGE <= 24	Age between 20 and 24	
5	25 <= DHH_AGE <= 29	Age between 25 and 29	
6	30 <= DHH_AGE <= 34	Age between 30 and 34	
7	35 <= DHH_AGE <= 39	Age between 35 and 39	
8	40 <= DHH_AGE <= 44	Age between 40 and 44	
9	45 <= DHH_AGE <= 49	Age between 45 and 49	
10	50 <= DHH_AGE <= 54	Age between 50 and 54	
11	55 <= DHH_AGE <= 59	Age between 55 and 59	
12	60 <= DHH_AGE <= 64	Age between 60 and 64	
13	65 <= DHH_AGE <= 69	Age between 65 and 69	
14	70 <= DHH_AGE <= 74	Age between 70 and 74	
15	75 <= DHH_AGE <= 79	Age between 75 and 79	
16	DHH_AGE >= 80	Age 80 and older	

2) Marital status - Grouped

Variable name: DHHGMS

Based on: DHH_MS

Description: This variable indicates the marital status for the selected respondent.

Specifications

Value	Condition(s)	Description	Notes
9	DHH_MS = (DK, R, NS)	Required question was not answered (don’t know, refusal, not stated)	NS
1	DHH_MS = 1	Married	
2	DHH_MS = 2	Common-law	
3	DHH_MS = 3, 4, 5	Widowed/Divorced/Separated	
4	DHH_MS = 6	Single	

3) Number of Persons in Household With Less Than 6 Years of Age - Grouped

Variable name: DHHGLE5

Based on: PERSONID, DHH_AGE

Description: This variable indicates the number of people living within the household whose age is less than 6 years old.

Note: The variable DHHDLE5 is derived by sorting the household roster dataset by SAMPLEID and PERSONID and by counting the number of PERSONIDs that have a DHH_AGE value of 5 and under within each SAMPLEID. DHHGLE5 is a regrouping of DHHDLE5.

Temporary Reformat

Value	Condition(s)	Description	Notes
**DHHDLE5**			
Total number of PERSONIDs within each SAMPLEID	DHH_AGE <= 5 (Member file)	Number of persons under 6 in a household	(values: 0-40)

Specifications

Value	Condition(s)	Description	Notes
0	DHHDLE5 = 0	No persons under 6 in the household	
1	DHHDLE5 >= 1	One or more persons under 6 in the household	

4) Number of Persons in Household between 6 to 11 Years of Age - Grouped

Variable name: DHHG611

Based on: PERSONID, DHH_AGE

Description: This variable indicates the number of people living within the household who are aged 6 to 11 years old.

Note: The variable DHHD611 is derived by sorting the household roster dataset by SAMPLEID and PERSONID and by counting the number of PERSONIDs that have a DHH_AGE value from 6 to 11 within each SAMPLEID.

Temporary Reformat

Value	Condition(s)	Description	Notes
**DHHED611**			
Total number of PERSONIDs within each SAMPLEID	(6 <= DHH_AGE <= 11) (Member file)	Number of persons aged 6 to 11 in a household	(min: 1; max: 40)

Specifications

Value	Condition(s)	Description	Notes
0	DHHD611 = 0	No persons aged 6 to 11 in the household	
1	DHHD611 => 1	One or more persons aged 6 to 11 in the household	

5) Number of Persons in Household With Less Than 12 Years of Age - Grouped

Variable name: DHHGL12

Based on: PERSONID, DHH_AGE, DHHDL12

Description: This variable indicates the number of people living within the household whose age is less than 12 years old.

Note: The variable DHHDL12 is derived by sorting the household roster dataset by SAMPLEID and PERSONID and by counting the number of PERSONIDs that have a DHH_AGE value less than 12 within each SAMPLEID. DHHGL12 is a regrouping of DHHDL12.

Temporary Reformat

Value	Condition(s)	Description	Notes
**DHHDL12**			
Total number of PERSONIDs within each SAMPLEID	DHH_AGE < 12 (Member file)	Number of persons under 12 in a household	(min: 0; max: 40)

Specifications

Value	Condition(s)	Description	Notes
0	DHHDL12 = 0	No persons under 12 in the household	
1	DHHDL12 >= 1	One or more persons under 12 in the household	

6) Living/Family Arrangement of Selected Respondent - Grouped

Variable name: DHHGLVG

Based on: DHH_REL of selected respondent, DHHDHSZ, DHHDLVG

Description: This variable identifies the family relationships between the selected respondent and the rest of the household.

Note: The necessary data are collected using a set of relationship codes that define a link between each pair of persons in a household. DHHGLVG is a regrouping of DHHDLVG.

Temporary Reformat

Value	Condition(s)	Description	Notes
**DHH_REL**			
L1	F5*, G0*, H0*, Z0	Temporary recodes to collapse relationships - Non-relative	RELATIONSHIP CODES: * All Foster relationships (foster sister/brother, parent, or child) have been recoded into the "Non relative" category due to the temporary nature of the relationships.
K1	I0, J0, K0, L0	Temporary recodes to collapse relationships - Other relative	RELATIONSHIP CODES:

C1	F0, F1, F2, F3, F4	Temporary recodes to collapse relationships - Sibling	RELATIONSHIP CODES:
B1	E0, E1, E2, E3	Temporary recodes to collapse relationships - Child	RELATIONSHIP CODES:
A1	D0, D1, D2, D3	Relationship codes used - Parental	RELATIONSHIP CODES:
Z1	ZZ, L8, L9	Temporary recodes to collapse relationships - Not stated	RELATIONSHIP CODES:
X1	A0, B0, C0	Temporary recodes to collapse relationships - Spouse/Partner	RELATIONSHIP CODES:

DHHDHSZ

Total number of PERSONIDs within each SAMPLEID	Sort the file (Member file) by SAMPLEID and PERSONID	Number of persons in a household	(min: 1; max: 40)

Specifications

Value	Condition(s)	Description	Notes
99	Any DHH_REL = Z1	Not stated	NS
1	DHHDHSZ = 1	Unattached individual living alone<br>(Selected respondent lives alone. Household size = 1)	
2	DHHDHSZ > 1 and<br>(no DHH_REL = X1) and<br>(no DHH_REL = A1) and<br>(no DHH_REL = B1)	Unattached individual living with others<br>(Selected respondent lives with others. He/she cannot have a marital/commonlaw or parental relationship but other relationships such as siblings are allowed)	
3	DHHDHSZ = 2 and<br>DHH_REL = X1	Spouse/partner living with spouse/partner<br>(Selected respondent lives with spouse/partner only. Household size = 2)	
4	DHHDHSZ > 2 and<br>one DHH_REL = X1 and<br>all other DHH_REL = A1	Parent living with spouse/partner and children<br>(Selected respondent lives with spouse/partner and one or more children)	
5	DHHDHSZ > 1 and<br>all DHH_REL = A1	Single parent living with children<br>(Selected respondent lives with one or more children. No other relationships are permitted)	
6	(DHHDHSZ = 2 and<br>DHH_REL = B1) or<br>(DHHDHSZ > 2 and<br>one DHH_REL = B1 and<br>all other DHH_REL = C1)	Selected respondent is a child living with a single parent with or without siblings	
7	(DHHDHSZ = 3 and<br>all DHH_REL = B1) or<br>(DHHDHSZ > 3 and<br>two DHH_REL = B1 and<br>all other DHH_REL = C1)	Selected respondent is a child living with two parents with or without siblings	
8	Else	Other<br>(Selected respondent lives in a household composition not classified above)	

7) Household size - Grouped

Variable name: DHHGHSZ

Based on: SAMPLEID, PERSONID, DHHDHSZ

Description: This variable indicates the number of people living within a household.

Note: This variable is derived by sorting the household roster dataset by SAMPLEID and PERSONID and by counting the number of PERSONIDs within each SAMPLEID. DHHGHSZ is a grouping of DHHDHSZ.

Specifications

Value	Condition(s)	Description	Notes
1	DHHDHSZ = 1	Exact number of persons living in household	
2	DHHDHSZ = 2	Exact number of persons living in household	
3	DHHDHSZ = 3	Exact number of persons living in household	
4	DHHDHSZ = 4	Exact number of persons living in household	
5	DHHDHSZ >= 5	Grouped - 5 or more persons live in the household	

Distress (3 DVs)

Both the K10 and K6 scale questions were developed from a pool of 612 questions drawn from existing distress and depression screening scales (Kessler RC, et al, 2002). After eliminating redundant and unclear questions, the remaining questions in the pool were organized to retain items consistent with 15 domains represented in the DSM-III-R diagnoses of major depression and generalized anxiety disorder plus the positive affect domain. These items were eventually reduced to those found in the K6 and K10 through processes involving ratings by an expert advisory panel, and analyses using item response theory of two subsequent pilot surveys. The final K10 and K6 scale questions were generated from the analysis of the telephone pilot survey using factor-analysis (Kessler RC. et al. 2002; http://www.hcp.med.harvard.edu/ncs/k6_scales.php)

The effectiveness of the K6 and K10 measurement scales of non-specific psychological distress were subsequently tested in the Australian National Survey of Mental Health and Well-Being against the criteria for the DSM-IV disorders and both scales performed well (Furukawa TA et al. 2003.)

DSM refers to the Diagnostic and Statistical Manual of Mental Disorders used by the American Psychiatric Association. It is an internationally recognized classification of mental disorders with several versions.

Temporary Reformat

Value	Condition(s)	Description	Notes
**DIST10A**			
(5 – DIS_10A)	DIS_10A <= 5	Rescale and invert the question answers from 1 to 5 to 4 to 0	
DIS_10A	DIS_10A > 5	Carry through cases of RF, DK, NS	
**DIST10B**			
DIS_10B	DIS_10B > 5	Carry through cases of RF, DK, NS	
(5 – DIS_10B)	DIS_10B <= 5	Rescale and invert the question answers from 1 to 5 to 4 to 0	
**DIST10C**			
DIS_10C	DIS_10C > 5	Carry through cases of RF, DK, NS	
(5 – DIS_10C)	DIS_10C <= 5	Rescale and invert the question answers from 1 to 5 to 4 to 0	
**DIST10D**			
DIS_10D	DIS_10D > 5	Carry through cases of RF, DK, NS	
(5 – DIS_10D)	DIS_10D <= 5	Rescale and invert the question answers from 1 to 5 to 4 to 0	
**DIST10E**			
DIS_10E	DIS_10E > 5	Carry through cases of RF, DK, NS	
(5 – DIS_10E)	DIS_10E <= 5	Rescale and invert the question answers from 1 to 5 to 4 to 0	
**DIST10F**			
DIS_10F	DIS_10F > 5	Carry through cases of RF, DK, NS	
(5 – DIS_10F)	DIS_10F <= 5	Rescale and invert the question answers from 1 to 5 to 4 to 0	
**DIST10G**			
DIS_10G	DIS_10G > 5	Carry through cases of RF, DK, NS	
(5 – DIS_10G)	DIS_10G <= 5	Rescale and invert the question answers from 1 to 5 to 4 to 0	
**DIST10H**			
DIS_10H	DIS_10H > 5	Carry through cases of RF, DK, NS	
(5 – DIS_10H)	DIS_10H <= 5	Rescale and invert the question answers from 1 to 5 to 4 to 0	
**DIST10I**			
(5 – DIS_10I)	DIS_10I <= 5	Rescale and invert the question answers from 1 to 5 to 4 to 0	
DIS_10I	DIS_10I > 5	Carry through cases of RF, DK, NS	
**DIST10J**			
DIS_10J	DIS_10J > 5	Carry through cases of RF, DK, NS	
(5 – DIS_10J)	DIS_10J <= 5	Rescale and invert the question answers from 1 to 5 to 4 to 0	

1) Distress Scale - K6

Variable name: DISDK6

Based on: DIS_10B, DIS_10D, DIS_10E, DIS_10H, DIS_10I, DIS_10J

Description: This variable determines the respondent's level of distress using six questions.

Note: This variable is based on 6 items and is known as the K6. Higher scores indicate more distress.

Internet site: http://www.hcp.med.havard.edu/ncs/k6_scales.php

Specifications

Value	Condition(s)	Description	Notes
96	DODIS = 2	Module not selected	NA
99	ADM_PRX = 1	Module not asked - proxy interview	NS
99	(DIST10B = DK, R, NS) or<br>(DIST10D = DK, R, NS) or<br>(DIST10E = DK, R, NS) or<br>(DIST10H = DK, R, NS) or<br>(DIST10I = DK, R, NS) or<br>(DIST10J = DK, R, NS)	At least one required question was not answered (don't know, refusal, not stated)	NS
DIST10B +<br>DIST10D +<br>DIST10E +<br>DIST10H +<br>DIST10I +<br>DIST10J	DIST10B <= 4 and<br>DIST10D <= 4 and<br>DIST10E <= 4 and<br>DIST10H <= 4 and<br>DIST10I <= 4 and<br>DIST10J <= 4	Score obtained on the distress scale (K6)	(min: 0; max: 24)

2) Chronicity of Distress and Impairment Scale

Variable name: DISDCHR

Based on: DIS_10K, DIS_10L, DIS_10M

Description: This variable classifies respondents according to the frequency of their distress feelings in the last month compared with usual.

Internet site: http://www.hcp.med.havard.edu/ncs/k6_scales.php

Specifications

Value	Condition(s)	Description	Notes
96	DODIS = 2	Module not selected	NA
99	ADM_PRX = 1	Module not asked - proxy interview	NS
99	(DIS_10K = DK, R, NS) or<br>(DIS_10L = DK, R, NS) or<br>(DIS_10M = DK, R, NS)	At least one required question was not answered (don't know, refusal, not stated)	NS
1	DIS_10L = 1	A lot more distress than usual	
2	DIS_10L = 2	Somewhat more distress than usual	
3	DIS_10L = 3	A little more distress than usual	
4	DIS_10K = 3	About the same distress as usual	
5	DIS_10M = 3	A little less distress than usual	

6	DIS_10M = 2	Somewhat less distress than usual
7	DIS_10M = 1	A lot less distress than usual
8	DIS_10K = 4	Never had any distress

3) Distress Scale - K10

Variable name: DISDDSX

Based on: DIS_10A, DIS_10B, DIS_10C, DIS_10D, DIS_10E, DIS_10F, DIS_10G, DIS_10H, DIS_10I, DIS_10J

Description: This variable determines the respondent’s level of distress using ten questions.

Note: This variable is based on 10 items and is known as the K10. Higher scores indicate more distress.

Internet site: http://www.hcp.med.harvard.edu/ncs/k6_scales.php

Specifications

Value	Condition(s)	Description	Notes
96	DODIS = 2	Module not selected	NA
99	ADM_PRX = 1	Module not asked - proxy interview	NS
99	(DIST10A = DK, R, NS) or<br>(DIST10B = DK, R, NS) or<br>(DIST10C = DK, R, NS) or<br>(DIST10D = DK, R, NS) or<br>(DIST10E = DK, R, NS) or<br>(DIST10F = DK, R, NS) or<br>(DIST10G = DK, R, NS) or<br>(DIST10H = DK, R, NS) or<br>(DIST10I = DK, R, NS) or<br>(DIST10J = DK, R, NS)	At least one required question was not answered (don’t know, refusal, not stated)	NS
DIST10A +<br>DIST10B +<br>DIST10C +<br>DIST10D +<br>DIST10E +<br>DIST10F +<br>DIST10G +<br>DIST10H +<br>DIST10I +<br>DIST10J	DIST10A <= 4 and<br>DIST10B <= 4 and<br>DIST10C <= 4 and<br>DIST10D <= 4 and<br>DIST10E <= 4 and<br>DIST10F <= 4 and<br>DIST10G <= 4 and<br>DIST10H <= 4 and<br>DIST10I <= 4 and<br>DIST10J <= 4	Score obtained on the distress scale (K10)	(min: 0; max: 40)

Depression (4 DVs)

The depression module used in CCHS is based on a long form of the Composite International Diagnostic Interview (CIDI) scale, which was developed in the late 1980s/early 1990s. This scale was never fully validated by the CIDI research team and its psychometric properties are therefore not well understood. Statistics Canada is currently exploring strategies to complete such a validation. At this time, Statistics Canada recommends that analysis of data from this module be restricted to examination of depression as a correlate of other health behaviours and characteristics. For now, use of the data as an indicator for the probability of depression or to calculate simple population prevalence is discouraged.

Temporary Reformat

Value	Condition(s)	Description	Notes
**DPST02**			
0	DPS_02 = 2	Rescale answers needed for calculation so that answers are 1 for yes and 0 for no	
1	DPS_02 = 1	Rescale answers needed for calculation so that answers are 1 for yes and 0 for no	
DPS_02	DPS_02 > 2	Carry through cases of RF, DK, NS	
**DPST05**			
0	DPS_05 = 2	Rescale answers needed for calculation so that answers are 1 for yes and 0 for no	
1	DPS_05 = 1	Rescale answers needed for calculation so that answers are 1 for yes and 0 for no	
DPS_05	DPS_05 > 2	Carry through cases of RF, DK, NS	
**DPST06**			
0	DPS_06 = 2	Rescale answers needed for calculation so that answers are 1 for yes and 0 for no	
1	DPS_06 = 1	Rescale answers needed for calculation so that answers are 1 for yes and 0 for no	
DPS_06	DPS_06 > 2	Carry through cases of RF, DK, NS	
**DPST08A**			
0	(DPS_07 = 3, 4) or [DPS _07 > 2 or (DPS_08A = DK, R, NS)]	For DPS_07, answers are rescaled so 0 = respondents whose weight stayed the same or were on a diet	
0	[DPS _07 <= 2 and (DPS_08A <> DK, R, NS)] and [(DPS_08A <= 9 and DPS_08B = 1) or (DPS_08A <= 4 and DPS_08B = 2)]	For DPS_08A, answers are rescaled so 1 = respondent gained or lost more than 4 kg (9 lbs.) and 0 if less or did not lose/gain weight	
1	[DPS _07 <= 2 and (DPS_08A <> DK, R, NS)] and [(DPS_08A > 9 and DPS_08B = 1) or (DPS_08A > 4 and DPS_08B = 2)]	For DPS_08A, answers are rescaled so 1 = respondent gained or lost more than 4 kg (9 lbs.) and 0 if less or did not lose/gain weight	
DPS_08A	Else	Rescale answers needed for calculation so that answers are 1 for yes and 0 for no	
**DPST10**			
0	DPS_10 = 3 or DPS_09 = 2	For DPS_10, answers are rescaled so 1 = respondent had trouble falling asleep every night or nearly every night and 0 if less often or not at all	
1	DPS_10 = 1, 2	For DPS_10, answers are rescaled so 1 = respondent had trouble falling asleep every night or nearly every night and 0 if less often or not at all	
DPS_10	DPS_10 > 3	Carry through cases of RF, DK, NS	
**DPST11**			
0	DPS_11 = 2	Rescale answers needed for calculation so that answers are 1 for yes and 0 for no	
1	DPS_11 = 1	Rescale answers needed for calculation so that answers are 1 for yes and 0 for no	
DPS_11	DPS_11 > 2	Carry through cases of RF, DK, NS	
**DPST12**			
0	DPS_12 = 2	Rescale answers needed for calculation so that answers are 1 for yes and 0 for no	

1	DPS_12 = 1	Rescale answers needed for calculation so that answers are 1 for yes and 0 for no
DPS_12	DPS_12 > 2	Carry through cases of RF, DK, NS
**DPST13**		
0	DPS_13 = 2	Rescale answers needed for calculation so that answers are 1 for yes and 0 for no
1	DPS_13 = 1	Rescale answers needed for calculation so that answers are 1 for yes and 0 for no
DPS_13	DPS_12 > 2	Carry through cases of RF, DK, NS
**DPST16**		
0	DPS_16 = 2	Rescale answers needed for calculation so that answers are 1 for yes and 0 for no
1	DPS_16 = 1	Rescale answers needed for calculation so that answers are 1 for yes and 0 for no
DPS_16	DPS_16 > 2	Carry through cases of RF, DK, NS
**DPST19**		
0	DPS_19 = 2	Rescale answers needed for calculation so that answers are 1 for yes and 0 for no
1	DPS_19 = 1	Rescale answers needed for calculation so that answers are 1 for yes and 0 for no
DPS_19	DPS_19 > 2	Carry through cases of RF, DK, NS
**DPST21A**		
0	(DPS_20 = 3, 4) or [DPS _20 > 2 or (DPS_21A = DK, R, NS)]	For DPS_21, answers are rescaled so 0 = respondents whose weight stayed the same or were on a diet
0	[DPS _20 <= 2 and (DPS_21A <> DK, R, NS)] and [(DPS_21A <= 9 and DPS_21B = 1) or (DPS_21A <= 4 and DPS_21B = 2)]	For DPS_21, answers are rescaled so 1 = respondent gained or lost more than 4 kg (9 lbs.) and 0 if less or did not lose/gain weight
1	[DPS _20 <= 2 and (DPS_21A <> DK, R, NS)] and [(DPS_21A > 9 and DPS_21B = 1) or (DPS_21A > 4 and DPS_21B = 2)]	For DPS_21 answers are rescaled so 1 = respondent gained or lost more than 4 kg (9 lbs.) and 0 if less or did not lose/gain weight
DPS_21A	Else	Rescale answers needed for calculation so that answers are 1 for yes and 0 for no
**DPST23**		
0	DPS_23 = 3 or DPS_22=2	For DPS_23 answers are rescaled so 1 = respondent had trouble falling asleep every night or nearly every night and 0 if less often or not at all
1	DPS_23 = 1, 2	For DPS_23 answers are rescaled so 1 = respondent had trouble falling asleep every night or nearly every night and 0 if less often or not at all
DPS_23	DPS_23 > 3	Carry through cases of RF, DK, NS
**DPST24**		
0	DPS_24 = 2	Rescale answers needed for calculation so that answers are 1 for yes and 0 for no
1	DPS_24 = 1	Rescale answers needed for calculation so that answers are 1 for yes and 0 for no
DPS_24	DPS_24 > 2	Carry through cases of RF, DK, NS
**DPST25**		
0	DPS_25 = 2	Rescale answers needed for calculation so that answers are 1 for yes and 0 for no
1	DPS_25 = 1	Rescale answers needed for calculation so that answers are 1 for yes and 0 for no
DPS_25	DPS_25 > 2	Carry through cases of RF, DK, NS
**DPST26**		
0	DPS_26 = 2	Rescale answers needed for calculation so that answers are 1 for yes and 0 for no

1	DPS_26 = 1	Rescale answers needed for calculation so that answers are 1 for yes and 0 for no
DPS_26	DPS_26 > 2	Carry through cases of RF, DK, NS

1) Derived Depression Scale - Short Form Score

Variable name: DPSDSF

Based on: DPS_02, DPS_05, DPS_06, DPS_08A, DPS_08B, DPS_10, DPS_11, DPS_12, DPS_13, DPS_16, DPS_17, DPS_18, DPS_19, DPS_21A, DPS_21B, DPS_23, DPS_24, DPS_25, DPS_26

Description: This variable assesses the depression level of respondents who felt depressed or lost interest in things for 2 weeks or more last year. These include normal periods of sadness (for example, after the death of a loved one), as well as "serious" depression.

Note: The items used to measure depression are based on the work of Kessler and Mroczek (from University of Michigan). They selected a subset of items from the Composite International Diagnostic Interview (CIDI) that measure major depressive episodes (MDE). The CIDI is a structure diagnostic instrument that was designed to produce diagnoses according to the definitions and the criteria of both DSM-lll-R and the Diagnostic Criteria for the Research of the ICD-10. The short-form of MDE used in the CCHS was developed to operationalize Criteria A through C of the DSM-III-R diagnosis of MDE. The diagnostic hierarchy rules defined in the Criterion D (not superimposed on schizophrenia, schizophrenia form disorder, delusional disorders, or psychotic disorders NOS) were ignored.

Higher scores indicate higher level of depression.

Internet site: National Comorbidity Survey: www.hcp.med.harvard.edu/ncs/
Composite International Diagnostic Interview (CIDI): www.who.int/msa/cidi/index.htm

Specifications

Value	Condition(s)	Description	Notes
96	DODEP = 2	Module not selected	NA
99	ADM_PRX = 1	Module not asked - proxy interview	NS
99	(DPST02 = DK, R, NS) or<br>(DPST05 = DK, R, NS) or<br>(DPST06 = DK, R, NS) or<br>(DPST08A = DK, R, NS) or<br>(DPST10 = DK, R, NS) or<br>(DPST11 = DK, R, NS) or<br>(DPST12 = DK, R, NS) or<br>(DPST13 = DK, R, NS) or<br>(DPST16 = DK, R, NS) or<br>(DPS_17 = DK, R, NS) or<br>(DPS_18 = DK, R, NS) or<br>(DPST19 = DK, R, NS) or<br>(DPST21A = DK, R, NS) or<br>(DPST23 = DK, R, NS) or<br>(DPST24 = DK, R, NS) or<br>(DPST25 = DK, R, NS) or<br>(DPST26 = DK, R, NS)	At least one required question was not answered (don't know, refusal, not stated)	NS
0	DPST02 < NA and<br>DPST05 = NA and<br>DPST19 = NA	Did not feel depressed or did not lose interest in things for two weeks last year, or did so only mildly (less than most of day and at least almost everyday for at least two weeks)	
DPST02 +<br>DPST05 +<br>DPST06 +<br>DPST08A +<br>DPST10 +<br>DPST11 +<br>DPST12 +<br>DPST13	DPST02 = 1 and<br>(DPST05 = 1, 0) and<br>(DPST06 = 1, 0) and<br>(DPST08A = 1, 0) and<br>(DPST10 = 1, 0) and<br>(DPST11 = 1, 0) and<br>(DPST12 = 1, 0) and<br>(DPST13 = 1, 0)	Felt depressed for 2 weeks or more last year	(min: 1; max: 8)

DPST16 +<br>DPST19 +<br>DPST21A +<br>DPST23 +<br>DPST24 +<br>DPST25 +<br>DPST26	DPST16 = 1 and<br>(DPST19 = 1, 0) and<br>(DPST21A = 1, 0) and<br>(DPST23 = 1, 0) and<br>(DPST24 = 1, 0) and<br>(DPST25 = 1, 0) and<br>(DPST26 = 1, 0)	Lost interest in things for 2 weeks or more last year	(min: 1; max: 7)

2) Depression Scale - Probability of Caseness to Respondents

Variable name: DPSDPP

Based on: DPSDSF

Description: This variable calculates from the score obtained on the depression scale, the probability (expressed as a proportion) that the respondent would have been diagnosed as having experienced a major depressive episode in the past 12 months, if they had completed the Long-Form Composite International Diagnostic Interview (CIDI).

Note: A probability of caseness of 0 was assigned to respondents who denied the stem questions.

Internet site: National Comorbidity Survey: www.hcp.med.harvard.edu/ncs/
Composite International Diagnostic Interview (CIDI): www.who.int/msa/cidi/index.htm

Specifications

Value	Condition(s)	Description	Notes
9.96	DODEP = 2	Module not selected	NA
9.99	ADM_PRX = 1	Module not asked - proxy interview	NS
9.99	DPSDSF = NS	At least one required question was not answered (don’t know, refusal, not stated) or module not asked (proxy interview)	NS
0	DPSDSF = 0	Probability of caseness to respondents	
0.05	DPSDSF = 1	Probability of caseness to respondents	
0.25	DPSDSF = 2	Probability of caseness to respondents	
0.50	DPSDSF = 3	Probability of caseness to respondents	
0.80	DPSDSF = 4	Probability of caseness to respondents	
0.90	DPSDSF > 4	Probability of caseness to respondents	

3) Number of Weeks Feeling Depressed - 12-Months

Variable name: DPSDWK

Based on: DPS_14, DPS_27

Description: This variable indicates the number of weeks the respondent felt depressed in the last 12 months.

Note: Respondents who did not show any required signs of depression have been excluded from the population.

Specifications

Value	Condition(s)	Description	Notes
96	DODEP = 2	Module not selected	NA
96	DPS_14 = NA and<br>DPS_27 = NA	Population exclusions	NA

99	ADM_PRX = 1	Module not asked - proxy interview	NS
99	(DPS_14 = DK, R, NS) or<br>(DPS_27 = DK, R, NS) or<br>(DPS_08A = DK, R, NS) or<br>(DPS_21A = DK, R, NS)	At least one required question was not answered (don't know, refusal, not stated)	NS
DPS_14	DPS_14 < NA	Number of weeks respondent felt sad, blue or depressed in the last year	
DPS_27	DPS_14 >= NA and<br>DPS_27 < NA	Number of weeks respondent lost interest in things in the last year	

4) Specific Month Last Felt Depressed

Variable name: DPSDMT

Based on: DPS_14, DPS_15, DPS_27, DPS_28

Description: This variable indicates the specific month when the respondent last felt depressed in the last year.

Note: The following respondents have been excluded from the population:
1) respondents who did not show any required signs of depression; or
2) respondents who have been depressed for more than 51 weeks in the past year

Specifications

Value	Condition(s)	Description	Notes
96	DODEP = 2	Module not selected	NA
96	DPS_15 = NA and<br>DPS_28 = NA	Population exclusions	NA
99	ADM_PRX = 1	Module not asked - proxy interview	NS
99	(DPS_14 = 52, DK, R, NS) or<br>(DPS_15 = DK, R, NS) or<br>(DPS_27 = 52, DK, R, NS) or<br>(DPS_28 = DK, R, NS) or<br>(DPS_08A = DK, R, NS) or<br>(DPS_21A = DK, R, NS)	Was depressed for >51 weeks last year or at least one required question was not answered (don't know, refusal, not stated)	NS
DPS_15	DPS_14 < 52 and<br>DPS_15 < NA	Specific month respondent felt sad, blue or depressed for at least 2 weeks in a row	(min : 1; max : 12)
DPS_28	DPS_14 >= NA and<br>DPS_27 < 52 and<br>DPS_28< NA	Specific month respondent last lost interest in things for at least 2 weeks in a row	(min : 1; max : 12)

Driving and safety (1 DV)

1) Passenger Seat Belt Use (Motor Vehicle)

Variable name: DRVFSBU

Based on: DRV_08A, DRV_08B

Description: This variable indicates whether the respondent always fastens his/her seatbelt when he/she is a front seat or back seat passenger in a car, truck or van.

Note: Those who are never a front-seat and never a rear-set passenger in a car, truck or van are excluded from the population.

Specifications

Value	Condition(s)	Description	Notes
6	DODRV = 2	Module not selected	NA
9	ADM_PRX = 1	Module not asked - proxy interview	NS
6	DRV_08A = 5 and<br>DRV_08B = 5	Population exclusions	NA
1	(DRV_08A = 1, 5) and<br>(DRV_08B = 1, 5)	Always fastens seatbelt when a passenger in a private vehicle	
2	(DRV_08A = 2, 3, 4) or<br>(DRV_08B = 2, 3, 4)	Does not always fasten seat belt when a passenger in a private vehicle	
9	(DRV_08A = DK, R, NS) or<br>(DRV_08B = DK, R, NS)	At least one required question was not answered (don’t know, refusal, not stated)	NS

Education (2 DVs)

1) Highest Level of Education - Household, 4 Levels

Variable name: EDUDH04

Based on: EDUDR04 for each member of the household

Description: This variable indicates the highest level of education acquired by any member of the household.

Note: This variable is derived by temporarily creating EDUDR04 for each member of the household (all PERSONID within SAMPLEID). The highest value is then obtained by comparing values of EDUDR04 for all members within the household. If any PERSONID has EDUDR04 of NS (not stated) then NS is returned. If all of EDUDR04 are NA (not applicable) then NA is returned.

2) Highest Level of Education - Respondent, 4 Levels

Variable name: EDUDR04

Based on: EDU_1, EDU_2, EDU_3, EDU_4

Description: This variable indicates the highest level of education acquired by the respondent.

Specifications

Value	Condition(s)	Description	Notes
1	[((EDU_1 = 1, 2) or EDU_2 = 2) and EDU_3 = 2]	Less than secondary school graduation	
2	EDU_2 = 1 and EDU_3 = 2	Secondary school graduation, no post-secondary education	
3	EDU_4 = 1	Some post-secondary education	
4	(2 <= EDU_4 <= 6)	Post-secondary degree/diploma	
9	(EDU_2 = DK, R, NS) or (EDU_3 = DK, R, NS) or (EDU_4 = DK, R, NS) or ((DHH_AGE = 14 or 15) and PMKPROXY = 2)	At least one required question was not answered (don’t know, refusal, not stated)	NS

Exposure to second-hand smoke (1 DV)

1) Number of people who smoke inside home - (G)

Variable name: ETSG11

Based on: ETS_11

Description: This variable groups the number of people who smoke inside the home.

Specifications

Value	Condition(s)	Description	Notes
6	ETS_11 = 96	Not applicable	
9	ETS_11 = 97, 98, or 99	Not stated	
	ETS_11 = 1	One person smokes inside the home.	
	ETS_11 = 2	Two people smoke inside the home.	
	ETS_11 = 3	Three people smoke inside the home.	
	ETS_11 >= 4	At least 4 people smoke inside the home.	

Food choices (3 DVs)

1) Avoids Certain Foods for Certain Content Reasons

Variable name: FDCFAVD

Based on: FDC_3A, FDC_3B, FDC_3C, FDC_3D, FDC_3E

Description: This variable indicates whether the respondent avoids certain foods because of concerns about fat, the type of fat, salt, cholesterol or calorie content.

Specifications

Value	Condition(s)	Description	Notes
6	DOFDC = 2	Module not selected	NA
9	ADM_PRX = 1	Module not asked - proxy interview	NS
2	FDC_3A = 2 and<br>FDC_3B = 2 and<br>FDC_3C = 2 and<br>FDC_3D = 2 and<br>FDC_3E = 2	Does not avoid certain foods because of concerns about fat, the type of fat, salt, cholesterol and calorie content	
1	FDC_3A = 1 or<br>FDC_3B = 1 or<br>FDC_3C = 1 or<br>FDC_3D = 1 or<br>FDC_3E = 1	Avoids certain foods because of concerns about fat, the type of fat, salt, cholesterol or calorie content	
9	(FDC_3A = DK, R, NS) or<br>(FDC_3B = DK, R, NS) or<br>(FDC_3C = DK, R, NS) or<br>(FDC_3D = DK, R, NS) or<br>(FDC_3E = DK, R, NS)	At least one required question was not answered (don't know, refusal, not stated)	NS

2) Chooses or Avoids Certain Foods Because of Certain Health Concerns

Variable name: FDCFCAH

Based on: FDC_1A, FDC_1B, FDC_1C, FDC_1D

Description: This variable indicates whether the respondent chooses or avoids certain types of foods because of one or more of the following health concerns: body weight, heart disease, cancer, and osteoporosis.

Specifications

Value	Condition(s)	Description	Notes
6	DOFDC = 2	Module not selected	NA
9	ADM_PRX = 1	Module not asked - proxy interview	NS
2	FDC_1A = 2 and<br>FDC_1B = 2 and<br>FDC_1C = 2 and<br>FDC_1D = 2	Does not choose or avoid certain foods because of health concerns related to body weight, heart disease, cancer, osteoporosis	
1	FDC_1A = 1 or<br>FDC_1B = 1 or<br>FDC_1C = 1 or<br>FDC_1D = 1	Choose or avoids certain foods because of health concerns related to body weight, heart disease, cancer or osteoporosis	

9	(FDC_1A = DK, R, NS) or<br>(FDC_1B = DK, R, NS) or<br>(FDC_1C = DK, R, NS) or<br>(FDC_1D = DK, R, NS)	At least one required question was not answered (don’t know, refusal, not stated)	NS

3) Chooses Certain Foods for Certain Content Reasons

Variable name: FDCFCHO

Based on: FDC_2A, FDC_2B, FDC_2C

Description: This variable indicates whether the respondent chooses certain foods because of concerns about fat, fibre, or calcium content.

Specifications

Value	Condition(s)	Description	Notes
6	DOFDC = 2	Module not selected	NA
9	ADM_PRX = 1	Module not asked - proxy interview	NS
2	FDC_2A = 2 and<br>FDC_2B = 2 and<br>FDC_2C = 2	Does not choose certain foods because of concerns about fat, fibre and calcium content	
1	FDC_2A = 1 or<br>FDC_2B = 1 or<br>FDC_2C = 1	Chooses certain foods because of concerns about fat, fibre or calcium content	
9	(FDC_2A = DK, R, NS) or<br>(FDC_2B = DK, R, NS) or<br>(FDC_2C = DK, R, NS)	At least one required question was not answered (don’t know, refusal, not stated)	NS

Food security (3 DVs)

Temporary Reformat

Value	Condition(s)	Description	Notes
**DHHTDKS**			
0	DHHDYKD = 0 and DHHDOKD = 0	Set value to 0 to indicate households WITHOUT children (aged less than 18)	
1	DHHDYKD <> 0 or DHHDOKD <> 0	Set value to 1 to indicate households WITH children (aged less than 18)	
**FSCASUM**			
FSCT020 + FSCT030 + FSCT040 + FSCT080 + FSCT081 + FSCT090 + FSCT100 + FSCT110 + FSCT120 + FSCT121	All	Sum of all temporary variables for adults to be used in determining the level of household food insecurity<br><br>Total will range from 0 to 10.	(Min: 0; Max: 10)
**FSCCSUM**			
FSCT050 + FSCT060 + FSCT070 + FSCT130 + FSCT140 + FSCT141 + FSCT150 + FSCT160	All	Sum of all temporary variables for children to be used in determining the level of household food insecurity<br><br>Total will range from 0 to 8.	(Min: 0; Max: 8)
**FSCT020**			
0	(FSC_020 = 3) and DOFSC = 1	Set the value to 0 if respondent did not provide an "affirmative" response to food security questions. Set the value to 1, if respondent did provide an "affirmative" response. See note above.	
1	(FSC_020 in (1, 2)) and DOFSC = 1	Set the value to 0 if respondent did not provide an "affirmative" response to food security questions. Set the value to 1, if respondent did provide an "affirmative" response. See note above.	
**FSCT030**			
0	(FSC_030 = 3) and DOFSC = 1	Set the value to 0 if respondent did not provide an "affirmative" response to food security questions. Set the value to 1, if respondent did provide an "affirmative" response. See note above.	
1	(FSC_030 in (1,2)) and DOFSC = 1	Set the value to 0 if respondent did not provide an "affirmative" response to food security questions. Set the value to 1, if respondent did provide an "affirmative" response. See note above.	
**FSCT040**			
0	(FSC_040 = 3) and DOFSC = 1	Set the value to 0 if respondent did not provide an "affirmative" response to food security questions. Set the value to 1, if respondent did provide an "affirmative" response. See note above.	
1	(FSC_040 in (1,2)) and DOFSC = 1	Set the value to 0 if respondent did not provide an "affirmative" response to food security questions. Set the value to 1, if respondent did provide an "affirmative" response. See note above.	
**FSCT050**			
0	(FSC_050 in (3,6)) and DOFSC = 1	Set the value to 0 if respondent did not provide an "affirmative" response to food security questions. Set the value to 1, if respondent did provide an "affirmative" response. See note above.	
1	(FSC_050 in (1,2)) and DOFSC = 1	Set the value to 0 if respondent did not provide an "affirmative" response to food security questions. Set the value to 1, if respondent did provide an "affirmative" response. See note above.	

**FSCT060**		
0	(FSC_060 in (3,6)) and DOFSC = 1	Set the value to 0 if respondent did not provide an "affirmative" response to food security questions. Set the value to 1, if respondent did provide an "affirmative" response. See note above.
1	(FSC_060 in (1, 2)) and DOFSC = 1	Set the value to 0 if respondent did not provide an "affirmative" response to food security questions. Set the value to 1, if respondent did provide an "affirmative" response. See note above.
**FSCT070**		
0	(FSC_070 in (3,6)) and DOFSC = 1	Set the value to 0 if respondent did not provide an "affirmative" response to food security questions. Set the value to 1, if respondent did provide an "affirmative" response. See note above.
1	(FSC_070 in (1,2)) and DOFSC = 1	Set the value to 0 if respondent did not provide an "affirmative" response to food security questions. Set the value to 1, if respondent did provide an "affirmative" response. See note above.
**FSCT080**		
0	(FSC_080 in (2,6)) and DOFSC = 1	Set the value to 0 if respondent did not provide an "affirmative" response to food security questions. Set the value to 1, if respondent did provide an "affirmative" response. See note above.
1	(FSC_080 = 1) and DOFSC = 1	Set the value to 0 if respondent did not provide an "affirmative" response to food security questions. Set the value to 1, if respondent did provide an "affirmative" response. See note above.
**FSCT081**		
0	(FSC_081 in (3,6)) and DOFSC = 1	Set the value to 0 if respondent did not provide an "affirmative" response to food security questions. Set the value to 1, if respondent did provide an "affirmative" response. See note above.
1	(FSC_081 in (1, 2)) and DOFSC = 1	Set the value to 0 if respondent did not provide an "affirmative" response to food security questions. Set the value to 1, if respondent did provide an "affirmative" response. See note above.
**FSCT090**		
0	(FSC_090 in (2,6)) and DOFSC = 1	Set the value to 0 if respondent did not provide an "affirmative" response to food security questions. Set the value to 1, if respondent did provide an "affirmative" response. See note above.
1	(FSC_090 = 1) and DOFSC = 1	Set the value to 0 if respondent did not provide an "affirmative" response to food security questions. Set the value to 1, if respondent did provide an "affirmative" response. See note above.
**FSCT100**		
0	(FSC_100 in (2,6)) and DOFSC = 1	Set the value to 0 if respondent did not provide an "affirmative" response to food security questions. Set the value to 1, if respondent did provide an "affirmative" response. See note above.
1	(FSC_100 = 1) and DOFSC = 1	Set the value to 0 if respondent did not provide an "affirmative" response to food security questions. Set the value to 1, if respondent did provide an "affirmative" response. See note above.
**FSCT110**		
0	(FSC_110 in (2,6)) and DOFSC = 1	Set the value to 0 if respondent did not provide an "affirmative" response to food security questions. Set the value to 1, if respondent did provide an "affirmative" response. See note above.
1	(FSC_110 = 1) and DOFSC = 1	Set the value to 0 if respondent did not provide an "affirmative" response to food security questions. Set the value to 1, if respondent did provide an "affirmative" response. See note above.
**FSCT120**		

0	(FSC_120 in (2,6)) and DOFSC = 1	Set the value to 0 if respondent did not provide an "affirmative" response to food security questions. Set the value to 1, if respondent did provide an "affirmative" response. See note above.
1	(FSC_120 = 1) and DOFSC = 1	Set the value to 0 if respondent did not provide an "affirmative" response to food security questions. Set the value to 1, if respondent did provide an "affirmative" response. See note above.
**FSCT121**		
0	(FSC_121 in (3,6)) and DOFSC = 1	Set the value to 0 if respondent did not provide an "affirmative" response to food security questions. Set the value to 1, if respondent did provide an "affirmative" response. See note above.
1	(FSC_121 in (1,2)) and DOFSC = 1	Set the value to 0 if respondent did not provide an "affirmative" response to food security questions. Set the value to 1, if respondent did provide an "affirmative" response. See note above.
**FSCT130**		
0	(FSC_130 in (2,6)) and DOFSC = 1	Set the value to 0 if respondent did not provide an "affirmative" response to food security questions. Set the value to 1, if respondent did provide an "affirmative" response. See note above.
1	(FSC_130 = 1) and DOFSC = 1	Set the value to 0 if respondent did not provide an "affirmative" response to food security questions. Set the value to 1, if respondent did provide an "affirmative" response. See note above.
**FSCT140**		
0	(FSC_140 in (2,6)) and DOFSC = 1	Set the value to 0 if respondent did not provide an "affirmative" response to food security questions. Set the value to 1, if respondent did provide an "affirmative" response. See note above.
1	(FSC_140 = 1) and DOFSC = 1	Set the value to 0 if respondent did not provide an "affirmative" response to food security questions. Set the value to 1, if respondent did provide an "affirmative" response. See note above.
**FSCT141**		
0	(FSC_141 in (3,6)) and DOFSC = 1	Set the value to 0 if respondent did not provide an "affirmative" response to food security questions. Set the value to 1, if respondent did provide an "affirmative" response. See note above.
1	(FSC_141 in (1,2)) and DOFSC = 1	Set the value to 0 if respondent did not provide an "affirmative" response to food security questions. Set the value to 1, if respondent did provide an "affirmative" response. See note above.
**FSCT150**		
0	(FSC_150 in (2,6)) and DOFSC = 1	Set the value to 0 if respondent did not provide an "affirmative" response to food security questions. Set the value to 1, if respondent did provide an "affirmative" response. See note above.
1	(FSC_150 = 1) and DOFSC = 1	Set the value to 0 if respondent did not provide an "affirmative" response to food security questions. Set the value to 1, if respondent did provide an "affirmative" response. See note above.
**FSCT160**		
0	(FSC_160 in (2,6)) and DOFSC = 1	Set the value to 0 if respondent did not provide an "affirmative" response to food security questions. Set the value to 1, if respondent did provide an "affirmative" response. See note above.
1	(FSC_160 = 1)and DOFSC = 1	Set the value to 0 if respondent did not provide an "affirmative" response to food security questions. Set the value to 1, if respondent did provide an "affirmative" response. See note above.

1) Household Food Security Status - Modified version

Variable name: FSCDHFS2

Based on: FSC_020, FSC_030, FSC_040, FSC_050, FSC_060, FSC_070, FSC_080, FSC_081, FSC_090, FSC_100, FSC_110, FSC_120, FSC_121, FSC_130, FSC_140, FSC_141, FSC_150, FSC_160

Description: This variable is based on a set of 18 questions and describes the food security situation of the household in the previous 12 months. It captures three kinds of situations:

1- Food secure: No, or one, indication of difficulty with income-related food access.
2- Moderately food insecure: Indication of compromise in quality and/or quantity of food consumed.
3- Severely food insecure: Indication of reduced food intake and disrupted eating patterns.

This variable is adopted from the Health Canada model of food security status.

Note: When using the person weight (WTS_M), this variable reflects the number of people living the household with food insecurity. When using the household weight (WTS_MHH), this variable reflects the number of households with food insecurity.

Households with children are defined as households with individuals who are either aged 15 or less (DHHDYKD=1), or aged 16 or 17 (DHHDOKD=1) and who are the child, grandchild, child-in-law, niece or nephew of another household member.

In order to determine household food security status, responses to each question are first coded as either "affirmative" or "negative". Some of this coding is obvious because the only response options are "yes" or "no". For questions with less obvious response categories, the procedure for coding is as follows: response categories such as "Often true", "Sometimes true", "Almost every month", "Some months but not every month" are coded as "affirmative" (i.e. coded equal to 1). Response categories such as "Never true", "Only 1 or 2 months" are coded as "negative" (i.e. coded equal to 0).

In 2009, an error in the model was corrected. Please see the Canadian Community Health Survey Errata for more information.

Internet site: http://www.hc-sc.gc.ca/fn-an/surveill/nutrition/commun/insecurit/status-situation-eng.php

Specifications

Value	Condition(s)	Description	Notes
6	DOFSC = 2	Module not selected	NA
9	(FSC_020 in (97,98,99)) or<br>(FSC_030 in (97,98,99)) or<br>(FSC_040 in (97,98,99)) or<br>(FSC_050 in (97,98,99)) or<br>(FSC_060 in (97,98,99)) or<br>(FSC_070 in (97,98,99)) or<br>(FSC_080 in (97,98,99)) or<br>(FSC_081 in (97,98,99)) or<br>(FSC_090 in (97,98,99)) or<br>(FSC_100 in (97,98,99)) or<br>(FSC_110 in (97,98,99)) or<br>(FSC_120 in (97,98,99)) or<br>(FSC_121 in (97,98,99)) or<br>(FSC_130 in (97,98,99)) or<br>(FSC_140 in (97,98,99)) or<br>(FSC_141 in (97,98,99)) or<br>(FSC_150 in (97,98,99)) or<br>(FSC_160 in (97,98,99)) or<br>PMKProxy = 2	At least one required question was not answered (don’t know, refusal, not stated) or the person most knowledgeable about the household was not available to answer questions for respondents aged 16 or younger.	NS
0	(DHHTDKS = 1 and<br>(FSCASUM >=0 and<br>FSCASUM <= 1) and<br>(FSCCSUM >=0 and<br>FSCCSUM <= 1)) or<br>(DHHTDKS = 0 and<br>(FSCASUM >= 0 and<br>FSCASUM <= 1))	Food secure	

1	[DHHTDKS = 1 and (FSCASUM >=2 and FSCASUM <= 5) and (FSCCSUM >= 2 and FSCCSUM <= 4)) or (DHHTDKS = 1 and (((FSCASUM >= 2 and FSCASUM <= 5) and (FSCCSUM <= 4)) or ((FSCASUM <= 5) and (FSCCSUM >= 2 and FSCCSUM <= 4)))) or (DHHTDKS = 0 and (FSCASUM >= 2 and FSCASUM <= 5))	Moderately food insecure	
2	(DHHTDKS = 1 and (FSCASUM >=6 and FSCASUM <= 10) or (FSCCSUM >= 5 and FSCCSUM <= 8)) or (DHHTDKS = 0 and (FSCASUM >=6 FSCASUM <= 10))	Severely food insecure	

Reference: The model for FSCDHFS2 is adopted from the Health Canada model of food security status levels published by Health Canada in 2007. For more information about this model, please see The Office of Nutrition Policy and Promotion, Health Canada, "Canadian Community Health Survey, Cycle 2.2, Nutrition (2004)-Income-Related Household Food Security in Canada".

2) Food Security - Adult Status

Variable name: FSCDAFS2

Based on: FSC_020, FSC_030, FSC_040, FSC_080, FSC_081, FSC_090, FSC_100, FSC_110, FSC_120, FSC_121

Description: This variable is based on a set of 10 adult-referenced questions and describes the food security situation of the adult members of the household. It captures three kinds of situations:

1-Food secure: No, or one, indication of difficulty with income-related food access.
2-Moderately food insecure: indication of compromise in quality and/or quantity of food consumed (2 to 5 affirmative responses).
3-Severely food insecure: indication of reduced food intake and disrupted eating patterns (>= 6 affirmative responses)

This variable is adopted from the Health Canada model of food security status.

Note: This variable does not necessarily reflect the experience of all adult members in the household. When using the person weights (WTS_M), this variable reflects the number of people living in households with food insecurity among the adult members of the household. When using the household weights (WTS_MHH), this variable reflects the number of households with food insecurity among the adult members of the household.

In order to determine household food security status, responses to each question are first coded as either "affirmative" or "negative". Some of this coding is obvious because the only response options are "yes" or "no". For questions with less obvious response categories, the procedure for coding is as follows: response categories such as "Often true", "Sometimes true", "Almost every month", "Some months but not every month" are coded as "affirmative" (i.e. coded equal to 1). Response categories such as "Never true", "Only 1 or 2 months" are coded as "negative" (i.e. coded equal to 0).

This derived variable was introduced in 2010.

Internet site: http://www.hc-sc.gc.ca/fn-an/surveill/nutrition/commun/insecurit/status-situation-eng.php

Specifications

Value	Condition(s)	Description	Notes
6	DOFSC = 2	Module not selected	NA

9	FSC_020 in (97,98,99) or FSC_030 in (97,98,99) or FSC_040 in (97,98,99) or FSC_080 in (97,98,99) or FSC_081 in (97,98,99) or FSC_090 in (97,98,99) or FSC_100 in (97,98,99) or FSC_110 in (97,98,99) or FSC_120 in (97,98,99) or FSC_121 in (97,98,99) or PMKPROXY = 2	At least one required question was not answered (don't know, refusal, not stated) or the person most knowledgeable about the household was not available to answer questions for respondents aged 16 or younger.	NS
0	(FSCASUM >= 0 and FSCASUM <= 1)	Food secure	
1	(FSCASUM >= 2 and FSCASUM <= 5)	Moderately food secure	
2	(FSCASUM >= 6 and FSCASUM <= 10)	Severely food insecure	

Reference: The model for FSCDAFS2 is adopted from the Health Canada model of food security status levels published by Health Canada in 2007. For more information about this model, please see The Office of Nutrition Policy and Promotion, Health Canada, "Canadian Community Health Survey, Cycle 2.2, Nutrition (2004)-Income-Related Household Food Security in Canada".

3) Food Security - Child Status

Variable name: FSCDCFS2

Based on: FSC_050, FSC_060, FSC_070, FSC_130, FSC_140, FSC_141, FSC_150, FSC_160

Description: This variable is based on a set of 8 child-referenced questions and describes the food security situation of the child (less than 18 years old) members of the household in the previous 12 months. It captures three kinds of situations:

1-Food secure: No, or one, indication of difficulty with income-related food access.
2-Moderately food insecure: indication of compromise in quality and/or quantity of food consumed (2 to 4 affirmative responses).
3-Severely food insecure: indication of reduced food intake and disrupted eating patterns (>= 5 affirmative responses)

This variable is adopted from the Health Canada model of food security status.

Note: This variable is only defined for households with individuals who are either aged 15 or less (DHHDYKD=1), or aged 16 or 17 (DHHDOKD=1) and who are the child, grandchild, child-in-law, niece or nephew of another household member. This variable does not necessarily reflect the experience of all child members in the household. When using the person weights (WTS_M), this variable reflects the number of people living in households with food insecurity among the child members of the household. When using the household weights (WTS_MHH), this variable reflects the number of households with food insecurity among the child members of the household.

In order to determine household food security status, responses to each question are first coded as either "affirmative" or "negative". Some of this coding is obvious because the only response options are "yes" or "no". For questions with less obvious response categories, the procedure for coding is as follows: response categories such as "Often true", "Sometimes true", "Almost every month", "Some months but not every month" are coded as "affirmative" (i.e. coded equal to 1). Response categories such as "Never true", "Only 1 or 2 months" are coded as "negative" (i.e. coded equal to 0).

This derived variable was introduced in 2010.

Internet site: http://www.hc-sc.gc.ca/fn-an/surveill/nutrition/commun/insecurit/status-situation-eng.php

Specifications

Value	Condition(s)	Description	Notes
6	DHHTDKS = 0	Population exclusions (households without children less than 18 years of age)	NA
6	DOFSC = 2	Module not selected	NA

9	(FSC_050 in (97,98,99)) or<br>(FSC_060 in (97,98,99)) or<br>(FSC_070 in (97,98,99)) or<br>(FSC_130 in (97,98,99)) or<br>(FSC_140 in (97,98,99)) or<br>(FSC_141 in (97,98,99)) or<br>(FSC_150 in (97,98,99)) or<br>(FSC_160 in (97,98,99)) or<br>PMKPROXY = 2	At least one required question was not answered (don’t know, refusal, not stated) or the person most knowledgeable about the house hold was not available to answer questions for respondents aged 16 or younger.	NS
0	DHHTDKS = 1 AND<br>(FSCCSUM >= 0 AND<br>FSCCSUM <= 1)	Food secure	
1	DHHTDKS = 1 AND<br>(FSCCSUM >= 2 AND<br>FSCCSUM <= 4)	Moderately food insecure	
2	DHHTDKS = 1 AND<br>(FSCCSUM >= 5 AND<br>FSCCSUM <= 8)	Severely food insecure	

Reference: The model for FSCDCFS2 is adopted from the Health Canada model of food security status levels published by Health Canada in 2007. For more information about this model, please see The Office of Nutrition Policy and Promotion, Health Canada, "Canadian Community Health Survey, Cycle 2.2, Nutrition (2004)-Income-Related Household Food Security in Canada".

Fruit and vegetable consumption (8 DVs)

1) Daily Consumption - Fruit Juice

Variable name: FVCDJUI

Based on: FVC_1A, FVC_1B, FVC_1C, FVC_1D, FVC_1E

Description: This variable indicates the usual number of times per day the respondent drinks fruit juice.

Note: The CCHS measures the number of times (frequency), not the amount consumed.

Specifications

Value	Condition(s)	Description	Notes
999.9	ADM_PRX = 1	Module not asked - proxy interview	NS
999.9	(FVC_1A = DK, R, NS) or (FVC_1B = DK, R, NS) or (FVC_1C = DK, R, NS) or (FVC_1D = DK, R, NS) or (FVC_1E = DK, R, NS)	At least one required question was not answered (don’t know, refusal, not stated)	NS
FVC_1B	FVC_1A = 1	Number of times/day	
FVC_1C / 7	FVC_1A = 2	Number of times/day (reported "times per week")	(rounded to one decimal place)
FVC_1D / 30	FVC_1A = 3	Number of times/day (reported "times per month")	(rounded to one decimal place)
FVC_1E / 365	FVC_1A = 4	Number of times/day (reported "times per year")	(rounded to one decimal place)
0	FVC_1A = 5	Never drinks fruit juice	

2) Daily Consumption - Other Fruit

Variable name: FVCDFRU

Based on: FVC_2A, FVC_2B, FVC_2C, FVC_2D, FVC_2E

Description: This variable indicates the usual number of times per day the respondent consumes fruit, excluding fruit juices.

Note: The CCHS measures the number of times (frequency), not the amount consumed.

Specifications

Value	Condition(s)	Description	Notes
999.9	ADM_PRX = 1	Module not asked - proxy interview	NS
999.9	(FVC_2A = DK, R, NS) or (FVC_2B = DK, R, NS) or (FVC_2C = DK, R, NS) or (FVC_2D = DK, R, NS) or (FVC_2E = DK, R, NS)	At least one required question was not answered (don’t know, refusal, not stated)	NS
FVC_2B	FVC_2A = 1	Number of times/day	
FVC_2C / 7	FVC_2A = 2	Number of times/day (reported "times per week")	(rounded to one decimal place)
FVC_2D / 30	FVC_2A = 3	Number of times/day (reported "times per month")	(rounded to one decimal place)

FVC_2E / 365	FVC_2A = 4	Number of times/day<br>(reported "times per year")	(rounded to one decimal place)
0	FVC_2A = 5	Never eats fruit	

3) Daily Consumption - Green Salad

Variable name: FVCDSAL

Based on: FVC_3A, FVC_3B, FVC_3C, FVC_3D, FVC_3E

Description: This variable indicates the usual number of times per day the respondent consumes green salad.

Note: The CCHS measures the number of times (frequency), not the amount consumed.

Specifications

Value	Condition(s)	Description	Notes
999.9	ADM_PRX = 1	Module not asked - proxy interview	NS
999.9	(FVC_3A = DK, R, NS) or<br>(FVC_3B = DK, R, NS) or<br>(FVC_3C = DK, R, NS) or<br>(FVC_3D = DK, R, NS) or<br>(FVC_3E = DK, R, NS)	At least one required question was not answered (don't know, refusal, not stated)	NS
FVC_3B	FVC_3A = 1	Number of times/day	
FVC_3C / 7	FVC_3A = 2	Number of times/day<br>(reported "times per week")	(rounded to one decimal place)
FVC_3D / 30	FVC_3A = 3	Number of times/day<br>(reported "times per month")	(rounded to one decimal place)
FVC_3E / 365	FVC_3A = 4	Number of times/day<br>(reported "times per year")	(rounded to one decimal place)
0	FVC_3A = 5	Never eats green salad	

4) Daily Consumption - Potatoes

Variable name: FVCDPOT

Based on: FVC_4A, FVC_4B, FVC_4C, FVC_4D, FVC_4E

Description: This variable indicates the usual number of times per day the respondent consumes potatoes, excluding French fries, fried potatoes, or potato chips.

Note: The CCHS measures the number of times (frequency), not the amount consumed.

Specifications

Value	Condition(s)	Description	Notes
999.9	ADM_PRX = 1	Module not asked - proxy interview	NS
999.9	(FVC_4A = DK, R, NS) or<br>(FVC_4B = DK, R, NS) or<br>(FVC_4C = DK, R, NS) or<br>(FVC_4D = DK, R, NS) or<br>(FVC_4E = DK, R, NS)	At least one required question was not answered (don't know, refusal, not stated)	NS
FVC_4B	FVC_4A = 1	Number of times/day	

FVC_4C / 7	FVC_4A = 2	Number of times/day<br>(reported "times per week")	(rounded to one decimal place)
FVC_4D / 30	FVC_4A = 3	Number of times/day<br>(reported "times per month")	(rounded to one decimal place)
FVC_4E / 365	FVC_4A = 4	Number of times/day<br>(reported "times per year")	(rounded to one decimal place)
0	FVC_4A = 5	Never eats potatoes	

5) Daily Consumption - Carrots

Variable name: FVCDCAR

Based on: FVC_5A, FVC_5B, FVC_5C, FVC_5D, FVC_5E

Description: This variable indicates the usual number of times per day the respondent consumes carrots.

Note: The CCHS measures the number of times (frequency), not the amount consumed.

Specifications

Value	Condition(s)	Description	Notes
999.9	ADM_PRX = 1	Module not asked - proxy interview	NS
999.9	(FVC_5A = DK, R, NS) or<br>(FVC_5B = DK, R, NS) or<br>(FVC_5C = DK, R, NS) or<br>(FVC_5D = DK, R, NS) or<br>(FVC_5E = DK, R, NS)	At least one required question was not answered (don't know, refusal, not stated)	NS
FVC_5B	FVC_5A = 1	Number of times/day	
FVC_5C / 7	FVC_5A = 2	Number of times/day<br>(reported "times per week")	(rounded to one decimal place)
FVC_5D / 30	FVC_5A = 3	Number of times/day<br>(reported "times per month")	(rounded to one decimal place)
FVC_5E / 365	FVC_5A = 4	Number of times/day<br>(reported "times per year")	(rounded to one decimal place)
0	FVC_5A = 5	Never eats carrots	

6) Daily Consumption - Other Vegetables

Variable name: FVCDVEG

Based on: FVC_6A, FVC_6B, FVC_6C, FVC_6D, FVC_6E

Description: This variable indicates the respondent's usual daily consumption of other vegetables, excluding carrots, potatoes, or salad. Respondents are asked to report in 'servings' rather than 'times' so that all different fruits or vegetables eaten at the same meal are counted. Servings should not be interpreted as referring to a specific quantity.

Note: In this question, the CCHS measures the number of servings, not the amount consumed.

Specifications

Value	Condition(s)	Description	Notes
999.9	ADM_PRX = 1	Module not asked -proxy interview	NS

999.9	(FVC_6A = DK, R, NS) or<br>(FVC_6B = DK, R, NS) or<br>(FVC_6C = DK, R, NS) or<br>(FVC_6D = DK, R, NS) or<br>(FVC_6E = DK, R, NS)	At least one required question was not answered (don’t know, refusal, not stated)	NS
FVC_6B	FVC_6A = 1	Number of servings/day	
FVC_6C / 7	FVC_6A = 2	Number of servings/day<br>(reported "servings per week")	(rounded to one decimal place)
FVC_6D / 30	FVC_6A = 3	Number of servings/day<br>(reported "servings per month")	(rounded to one decimal place)
FVC_6E / 365	FVC_6A = 4	Number of servings/day<br>(reported "servings per year")	(rounded to one decimal place)
0	FVC_6A = 5	Never eats other vegetables	

7) Daily Consumption - Total Fruit and Vegetable

Variable name: FVCDTOT

Based on: FVCDJUI, FVCDFRU, FVCDSAL, FVCDPOT, FVCDCAR, FVCDVEG

Description: This variable indicates the total number of times per day the respondent eats fruits and vegetables.

Note: The CCHS measures the number of times (frequency), not the amount consumed.

Specifications

Value	Condition(s)	Description	Notes
999.9	ADM_PRX = 1	Module not asked - proxy interview	NS
999.9	FVCDJUI = NS or<br>FVCDFRU = NS or<br>FVCDSAL = NS or<br>FVCDPOT = NS or<br>FVCDCAR = NS or<br>FVCDVEG = NS	At least one required question was not answered (don't know, refusal, not stated)	NS
FVCDJUI +<br>FVCDFRU +<br>FVCDSAL +<br>FVCDPOT +<br>FVCDCAR +<br>FVCDVEG	(0 <= FVCDJUI <= 20) and<br>(0 <= FVCDFRU <= 20) and<br>(0 <= FVCDSAL <= 20) and<br>(0 <= FVCDPOT <= 20) and<br>(0 <= FVCDCAR <= 20) and<br>(0 <= FVCDVEG <= 20)	Total number of times the respondent eats fruits and vegetables	(min : 0.0; max : 120.0)

8) Grouping of Daily Consumption - Total Fruit and Vegetable

Variable name: FVCGTOT

Based on: FVCDTOT

Description: This variable classifies the respondent based on the total number of times per day he/she eats fruits and vegetables.

Note: The CCHS measures the number of times (frequency), not the amount consumed.

Specifications

Value	Condition(s)	Description	Notes
9	ADM_PRX = 1	Module not asked - proxy interview	NS

9	FVCDTOT = NS	At least one required question was not answered (don't know, refusal, not stated)	NS
1	FVCDTOT < 5	Eats fruits and vegetables less than 5 times per day.	
2	(5 <= FVCDTOT <= 10)	Eats fruits and vegetables between 5 and 10 times per day	
3	FVCDTOT > 10	Eats fruits and vegetables more than 10 times per day	

General health (3 DVs)

1) Perceived Health

Variable name: GENDHDI

Based on: GEN_01

Description: This variable indicates the respondent's health status based on his/her own judgement or his/her proxy. Higher scores indicate positive perceived health status.

Note: Prior to 2007, this variable was named self-rated health.

Specifications

Value	Condition(s)	Description	Notes
9	(GEN_01 = DK, R, NS)	Required question was not answered (don’t know, refusal, not stated)	NS
0	GEN_01 = 5	Poor	
1	GEN_01 = 4	Fair	
2	GEN_01 = 3	Good	
3	GEN_01 = 2	Very good	
4	GEN_01 = 1	Excellent	

2) Perceived Mental Health

Variable name: GENDMHI

Based on: GEN_02B

Description: This variable indicates the respondent's mental health status based on his/her own judgement. Higher scores indicate positive perceived mental health status.

Note: Prior to 2007, this variable was named self-rated mental health.

Specifications

Value	Condition(s)	Description	Notes
9	ADM_PRX = 1	Module not asked - proxy interview	NS
9	(GEN_02B = DK, R, NS)	Required question was not answered (don’t know, refusal, not stated)	NS
0	GEN_02B = 5	Poor	
1	GEN_02B = 4	Fair	
2	GEN_02B = 3	Good	
3	GEN_02B = 2	Very good	
4	GEN_02B = 1	Excellent	

3) Satisfaction with life in general - (G)

Variable name: GENGSWL

Based on: GEN_02A2

Description: This variable groups the 11-point scale used in GEN_02A2 to rate a respondent's satisfaction with life into 5 categories. The 5 categories were used for GEN_02A prior to 2009.

Note: This variable is available for the purpose of comparing data from question GEN_02A produced previous to 2009. Users should be aware that although a good concordance was determined, GEN_02A was based on a 5-point answer category vs. an 11-point scale for the variable GEN_02A2.

Specifications

Value	Condition(s)	Description	Notes
9	ADM_PRX = 1	Question not asked - proxy interview	NS
9	GEN_02A2 in (97,98,99)	At least one required question was not answered (don't know, refusal, not stated)	NS
1	(GEN_02A2 >= 9 and GEN_02A2 <= 10)	Very Satisfied	
2	(GEN_02A2 >= 6 and GEN_02A2 <= 8)	Satisfied	
3	GEN_02A2 = 5	Neither satisfied nor dissatisfied	
4	(GEN_02A2 >= 2 and GEN_02A2 <= 4)	Dissatisfied	
5	(GEN_02A2 >= 0 and GEN_02A2 <= 1)	Very Dissatisfied	

Geography variables (3 DVs)

The January 2009 Postal Code Conversion File (PCCF) was used in the derivation of the geographic variables. All geographic variables use the geography from the 2006 Census except for GEODDA01 and GEODCMA1, which use the 2001 Census.

1) Province of residence of respondent - (G)

Variable name: GEOGPRV

Based on: GEO_PRV

Description: This is the respondent's province of residence.

Specifications

Value	Condition(s)	Description	Notes
GEO_PRV	GEO_PRV=<59	Province of residence of respondent.	
60	60=<GEO_PRV=<62	Yukon/Northwest/Nunavut Territories	

2) Health region - Grouped

Variable name: GEODPMF

Based on: GEODHR4

Description: This variable is a 5-digit number that identifies the sub-provincial health areas. It is based on the 4-digit health regions specified by the Provincial Ministries of Health. This reconstruction is as follows:
- positions 1-2 (first two positions of GEODHR4);
- position 3 (value of "9");
- positions 4-5 (3rd, 4th position of GEODHR4).

Note: The variable GEODHR4 is the health region based on GEODPC (postal code) and is derived using the information available on the survey frame at the time of sampling and the geographic information provided by the respondent. GEODHR4 and GEODPC are not included in the Public Use Microdata File.

Specifications

Value	Condition(s)	Description	Notes
10913	GEODHR4 in (1013, 1014)	Group:<br>Western Regional Integrated Health Authority<br>Labrador-Grenfell Integrated Health Authority	
11901	GEODHR4 in (1101, 1102, 1103)	Group:<br>Prince Health Region<br>Kings Health Region<br>Queens Health Region	
13904	GEODHR4 in (1304, 1305)	Group:<br>Zone 4<br>Zone 5	
13906	GEODHR4 in (1306, 1307)	Group:<br>Zone 6<br>Zone 7	
35939	GEODHR4 in (3539, 3554)	Group:<br>Huron County Health Unit<br>Perth District Health Unit	
35947	GEODHR4 in (3547, 3563)	Group:<br>North Bay Parry Sound District Health Unit<br>Timiskaming Health Unit	

46915	GEODHR4 in (4615, 4645)	Group:<br>Brandon Regional Health Authority<br>Assiniboine Regional Health Authority	
46920	GEODHR4 in (4620, 4625)	Group:<br>North Eastman Regional Health Authority<br>South Eastman Regional Health Authority	
46931	GEODHR4 in (4630, 4660)	Group:<br>Interlake Regional Health Authority<br>Parkland Regional Health Authority	New grouping for 2009-2010 PUMF
46970	GEODHR4 in (4670, 4685)	Group:<br>NOR-MAN Regional Health Authority<br>Burntwood Regional Health Authority/Churchill Regional Health Authority	New grouping for 2009-2010 PUMF
47901	GEODHR4 in (4701, 4702, 4703)	Group:<br>Sun Country Regional Health Authority<br>Five Hills Regional Health Authority<br>Cypress Regional Health Authority	
47905	GEODHR4 in (4705, 4708)	Group:<br>Sunrise Regional Health Authority<br>Kelsey trail Regional Health Authority	
47907	GEODHR4 in (4707, 4710)	Group:<br>Heartland Regional Health Authority<br>Prairie North Regional Health Authority	
47909	GEODHR4 in (4709, 4714)	Group:<br>Prince Albert Parkland Regional Health Authority<br>Mamawetan Churchill River RHA/Keewatin Yatthé RHA/Athabasca Health Authority	
60901	GEODHR4 in (6001, 6101, 6201)	Group:<br>Yukon<br>Northwest Territories<br>Nunavut	

3) Health Authority - British Columbia

Variable name: GEODBCHA

Based on: GEODPC

Description: This variable is a 4-digit number that identifies the 5 Health Authorities for British Columbia. It is equal to 9996 (for not applicable) anywhere else. This variable is derived using the information available on the survey frame at the time of sampling and the geographic information provided by the respondent. As of 2009, this variable is based on the dissemination areas from the 2006 Census.

Health utilities index (8 DVs)

The Health Utilties Index (HUI) is a multi-attribute health status classification system for measuring generic health status and health-related quality of life. The version used by CCHS has been adapted from the HUI Mark 3 (HUI3) for NPHS. The questions are slighlty different than the original HUI3 developed at McMaster University. This instrument allows the calculation of a generic health status index based on attributes found in two different CCHS modules - the Health utilities index (HUI) and Health utilities index - Pain and discomfort (HUP). For more information see "Feeny D, Furlong W, Torrance GW et al. Multi-attribute and single-attribute utility functions for the Health Utilities Index Mark 3 system. Med Care 2002; 40: 113-128."

1) Vision Trouble (Function Code) - Grouped

Variable name: HUIGVIS

Based on: HUI_01, HUI_02, HUI_03, HUI_04, HUI_05

Description: This variable classifies the respondents based on their vision state.

Specifications

Value	Condition(s)	Description	Notes
96	DOHUI = 2	Module not selected	NA
99	(HUI_01 = DK, R, NS) or<br>(HUI_02 = DK, R, NS) or<br>(HUI_03 = DK, R, NS) or<br>(HUI_04 = DK, R, NS) or<br>(HUI_05 = DK, R, NS)	At least one required question was not answered (don't know, refusal,<br>not stated)	NS
1	HUI_01 = 1 and<br>HUI_02 = 6 and<br>HUI_03 = 6 and<br>HUI_04 = 1 and<br>HUI_05 = 6	No visual problems	
2	(HUI_01 = 1 and<br>HUI_02 = 6 and<br>HUI_03 = 6 and<br>HUI_04 = 2 and<br>HUI_05 = 1)<br>or<br>(HUI_01 = 2 and<br>HUI_02 = 1 and<br>HUI_03 = 6 and<br>HUI_04 = 1 and<br>HUI_05 = 6)<br>or<br>(HUI_01 = 2 and<br>HUI_02 = 1 and<br>HUI_03 = 6 and<br>HUI_04 = 2 and<br>HUI_05 = 1)	Problems corrected by lenses (distance, close, or both)	
3	(HUI_01 = 1 and<br>HUI_02 = 6 and<br>HUI_03 = 6 and<br>HUI_04 = 2 and<br>HUI_05 = 2)<br>or<br>(HUI_01 = 2 and<br>HUI_02 = 1 and<br>HUI_03 = 6 and<br>HUI_04 = 2 and<br>HUI_05 = 2)	Problems seeing distance - not corrected	

4	(HUI_01 = 2 and<br>HUI_02 = 2 and<br>HUI_03 = 1 and<br>HUI_04 = 1 and<br>HUI_05 = 6)<br>or<br>(HUI_01 = 2 and<br>HUI_02 = 2 and<br>HUI_03 = 1 and<br>HUI_04 = 2 and<br>HUI_05 = 1)	Problems seeing close - not corrected	
5	HUI_01 = 2 and<br>HUI_02 = 2 and<br>HUI_03 = 1 and<br>HUI_04 = 2 and<br>HUI_05 = 2<br>or<br>HUI_01 = 2 and<br>HUI_02 = 2 and<br>HUI_03 = 2 and<br>HUI_04 = 6 and<br>HUI_05 = 6	Problem seeing close and distance - not corrected,<br>or no sight at all	

2) Hearing Problems (Function Code) - Grouped

Variable name: HUIGHER

Based on: HUI_06, HUI_07, HUI_07A, HUI_08, HUI_09

Description: This variable classifies the respondents based on their hearing state.

Specifications

Value	Condition(s)	Description	Notes
96	DOHUI = 2	Module not selected	NA
99	(HUI_06 = DK, R, NS) or<br>(HUI_07 = DK, R, NS) or<br>(HUI_07A = DK, R, NS) or<br>(HUI_08 = DK, R, NS) or<br>(HUI_09 = DK, R, NS)	At least one required question was not answered<br>(don't know, refusal,<br>not stated)	NS
1	HUI_06 = 1 and<br>HUI_07 = 6 and<br>HUI_07A = 6 and<br>HUI_08 = 6 and<br>HUI_09 = 6	No hearing problems	
2	HUI_06 = 2 and<br>HUI_07 = 1 and<br>HUI_07A = 6 and<br>HUI_08 = 1 and<br>HUI_09 = 6<br>or<br>(HUI_06 = 2 and<br>HUI_07 = 1 and<br>HUI_07A = 6 and<br>HUI_08 = 2 and<br>HUI_09 = 1)<br>or<br>(HUI_06 = 2 and<br>HUI_07 = 1 and<br>HUI_07A =6 and<br>HUI_08 = 2 and<br>HUI_09 = 2)	Problem hearing in group - corrected<br>Or<br>Problem hearing in group and individual - corrected	

3	HUI_06 = 2 and<br>HUI_07 = 2 and<br>HUI_07A =1 and<br>HUI_08 = 1 and<br>HUI_09 = 6<br>or<br>HUI_06 = 2 and<br>HUI_07 = 2 and<br>HUI_07A =1 and<br>HUI_08 = 2 and<br>HUI_09 = 1<br>or<br>(HUI_06 = 2 and<br>HUI_07 = 2 and<br>HUI_07A =1 and<br>HUI_08 = 2 and<br>HUI_09 = 2)<br>or<br>(HUI_06 = 2 and<br>HUI_07 = 2 and<br>HUI_07A =2 and<br>HUI_08 = 6 and<br>HUI_09 = 6)	Problem hearing in group - not corrected<br>Or<br>Problem hearing in group and individual - individual corrected<br>Or<br>Cannot hear	

3) Speech Trouble (Function Code) - Grouped

Variable name: HUIGSPE

Based on: HUI_10, HUI_11, HUI_12, HUI_13

Description: This variable classifies the respondents based on their state of speech trouble.

Specifications

Value	Condition(s)	Description	Notes
6	DOHUI = 2	Module not selected	NA
9	(HUI_010 = DK, R, NS) or<br>(HUI_011 = DK, R, NS) or<br>(HUI_012 = DK, R, NS) or<br>(HUI_013 = DK, R, NS)	At least one required question was not answered (don’t know, refusal, not stated)	NS
1	HUI_10 = 1 and<br>HUI_11 = 6 and<br>HUI_12 = 6 and<br>HUI_13 = 6	No speech problems	

2	HUI_10 = 2 and<br>HUI_11 = 1 and<br>HUI_12 = 1 and<br>HUI_13 = 6<br>or<br>HUI_10 = 2 and<br>HUI_11 = 1 and<br>HUI_12 = 2 and<br>HUI_13 = 1<br>or<br>(HUI_10 = 2 and<br>HUI_11 = 2 and<br>HUI_12 = 1 and<br>HUI_13 = 6)<br>or<br>(HUI_10 = 2 and<br>HUI_11 = 2 and<br>HUI_12 = 2 and<br>HUI_13 = 1)<br>or<br>(HUI_10 = 2 and<br>HUI_11 = 1 and<br>HUI_12 = 2 and<br>HUI_13 = 2)<br>or<br>(HUI_10 = 2 and<br>HUI_11 = 2 and<br>HUI_12 = 2 and<br>HUI_13 = 2)	Partially understood by strangers<br>Or<br>Partially understood by friends<br>Or<br>Not understood by strangers<br>Or<br>Not understood by friends	

4) Mobility Trouble (Function Code) - Grouped

Variable name: HUIGMOB

Based on: HUI_14, HUI_15, HUI_16, HUI_17, HUI_18

Description: This variable classifies the respondents based on their state of mobility trouble.

Specifications

Value	Condition(s)	Description	Notes
96	DOHUI = 2	Module not selected	NA
99	(HUI_14 = DK, R, NS) or<br>(HUI_15 = DK, R, NS) or<br>(HUI_16 = DK, R, NS) or<br>(HUI_17 = DK, R, NS) or<br>(HUI_18 = DK, R, NS)	At least one required question was not answered (don’t know, refusal, not stated)	NS
1	HUI_14 = 1 and<br>HUI_15 = 6 and<br>HUI_16 = 6 and<br>HUI_17 = 6 and<br>HUI_18 = 6	No mobility problems	
2	HUI_14 = 2 and<br>HUI_15 = 1 and<br>HUI_16 = 2 and<br>HUI_17 = 2 and<br>HUI_18 = 2	Problem - no aid required	

3	HUI_14 = 2 and<br>HUI_15 = 1 and<br>HUI_16 = 1 and<br>HUI_17 = 2 and<br>HUI_18 = 2<br>or<br>(HUI_14 = 2 and<br>HUI_15 = 1 and<br>HUI_16 = 1 and<br>HUI_17 = 2 and<br>HUI_18 = 1)<br>or<br>(HUI_14 = 2 and<br>HUI_15 = 1 and<br>HUI_16 = 2 and<br>HUI_17 = 2 and<br>HUI_18 = 1)	Problem - requires mechanical support or wheelchair	
4	(HUI_14 = 2 and<br>HUI_15 = 1 and<br>HUI_16 = 1 and<br>HUI_17 = 1 and<br>HUI_18 = 1)<br>or<br>(HUI_14 = 2 and<br>HUI_15 = 1 and<br>HUI_16 = 1 and<br>HUI_17 = 1 and<br>HUI_18 = 2)<br>or<br>(HUI_14 = 2 and<br>HUI_15 = 1 and<br>HUI_16 = 2 and<br>HUI_17 = 1 and<br>HUI_18 = 1)<br>or<br>(HUI_14 = 2 and<br>HUI_15 = 1 and<br>HUI_16 = 2 and<br>HUI_17 = 1 and<br>HUI_18 = 2)<br>or<br>(HUI_14 = 2 and<br>HUI_15 = 2 and<br>HUI_16 = 6 and<br>HUI_17 = 6 and<br>HUI_18 = 1)<br>or<br>(HUI_14 = 2 and<br>HUI_15 = 2 and<br>HUI_16 = 6 and<br>HUI_17 = 6 and<br>HUI_18 = 2)	Problem - requires help from people<br>Or<br>Cannot walk	

5) Dexterity Trouble (Function Code) - Grouped

Variable name: HUIGDEX

Based on: HUI_21, HUI_22, HUI_23, HUI_24

Description: This variable classifies the respondents based on their state of dexterity trouble.

Specifications

Value	Condition(s)	Description	Notes
96	DOHUI = 2	Module not selected	NA

99	(HUI_21 = DK, R, NS) or<br>(HUI_22 = DK, R, NS) or<br>(HUI_23 = DK, R, NS) or<br>(HUI_24 = DK, R, NS)	At least one required question was not answered (don't know, refusal, not stated)	NS
1	HUI_21 = 1 and<br>HUI_22 = 6 and<br>HUI_23 = 6 and<br>HUI_24 = 6	No dexterity problems	
2	HUI_21 = 2 and<br>HUI_22 = 2 and<br>HUI_23 = 6 and<br>HUI_24 = 2	Dexterity problem - no help required	
3	HUI_21 = 2 and<br>HUI_22 = 2 and<br>HUI_23 = 6 and<br>HUI_24 = 1<br>or<br>(HUI_21 = 2 and<br>HUI_22 = 1 and<br>HUI_23 = 1 and<br>HUI_24 = 1)<br>or<br>(HUI_21 = 2 and<br>HUI_22 = 1 and<br>HUI_23 = 1 and<br>HUI_24 = 2)<br>or<br>(HUI_21 = 2 and<br>HUI_22 = 1 and<br>HUI_23 = 2 and<br>HUI_24 = 1)<br>or<br>(HUI_21 = 2 and<br>HUI_22 = 1 and<br>HUI_23 = 2 and<br>HUI_24 = 2)<br>or<br>(HUI_21 = 2 and<br>HUI_22 = 1 and<br>HUI_23 = 3 and<br>HUI_24 =1)<br>or<br>(HUI_21 = 2 and<br>HUI_22 = 1 and<br>HUI_23 = 3 and<br>HUI_24 = 2)<br>or<br>(HUI_21 = 2 and<br>HUI_22 = 1 and<br>HUI_23 = 4 and<br>HUI_24 = 1)<br>or<br>(HUI_21 = 2 and<br>HUI_22 = 1 and<br>HUI_23 = 4 and<br>HUI_24 = 2)	Dexterity problem - require special equipment or help with some tasks or help with most tasks or help with all tasks	

6) Emotion Health Status

Variable name: HUIDEMO

Based on: HUI_25

Description: Emotion Health Status refers to a person's emotional well-being. This is based on different levels of happiness and interest in life, and unhapiness.

Specifications			
**Value**	**Condition(s)**	**Description**	**Notes**
6	DOHUI = 2	Module not selected	NA
1	HUI_25 = 1	Happy and interested in life	
2	HUI_25 = 2	Somewhat happy	
3	HUI_25 = 3	Somewhat unhappy	
4	HUI_25 = 4	Very unhappy	
5	HUI_25 = 5	So unhappy that life is not worthwhile	
9	(HUI_25 = DK, R, NS)	Required question was not answered (don't know, refusal, not stated)	NS

Reference: Reference: For more information on the Health Utilities Index and more details on each category please see http://www.statcan.gc.ca/subjects-sujets/standard-norme/otherclass-subject-autreclass-sujet-eng.htm.

7) Cognition Health Status

Variable name: HUIDCOG

Based on: HUI_26, HUI_27

Description: Cognition health status refers to a person's cognition facility based on his or her ability to remember, think and solve problems.

Specifications			
**Value**	**Condition(s)**	**Description**	**Notes**
96	DOHUI = 2	Module not selected	NA
1	HUI_26 = 1 and<br>HUI_27 = 1	Able to remember and think	
2	(HUI_26 = 1 and<br>HUI_27 = 2)<br>or<br>(HUI_26 = 1 and<br>HUI_27 = 3)	Able to remember and some difficulty thinking	
3	HUI_26 = 2 and<br>HUI_27 = 1	Somewhat forgetful and able to think	
4	(HUI_26 = 2 and<br>HUI_27 = 2)<br>or<br>(HUI_26 = 2 and<br>HUI_27 = 3)	Somewhat forgetful and some difficulty thinking	
5	(HUI_26 = 1 and<br>HUI_27 = 4)<br>or<br>(HUI_26 = 2 and<br>HUI_27 = 4)<br>or<br>(HUI_26 = 3 and<br>HUI_27 = 1)<br>or<br>(HUI_26 = 3 and<br>HUI_27 = 2)<br>or<br>(HUI_26 = 3 and<br>HUI_27 = 3)<br>or<br>(HUI_26 = 3 and<br>HUI_27 = 4)	Very forgetful or great deal of difficulty thinking	

6	(HUI_26 = 1 and HUI_27 = 5) or (HUI_26 = 2 and HUI_27 = 5) or (HUI_26 = 3 and HUI_27 = 5) or (HUI_26 = 4 and HUI_27 = 1) or (HUI_26 = 4 and HUI_27 = 2) or (HUI_26 = 4 and HUI_27 = 3) or (HUI_26 = 4 and HUI_27 = 4) or (HUI_26 = 4 and HUI_27 = 5)	Unable to remember or unable to think	
99	(HUI_26 = DK, R, NS) or (HUI_27 = DK, R, NS)	At least one required question was not answered (don't know, refusal, not stated)	NS

Reference: Reference: For more information on the Health Utilities Index and more details on each category please see http://www.statcan.gc.ca/subjects-sujets/standard-norme/otherclass-subject-autreclass-sujet-eng.htm.

8) Health Utilities Index

Variable name: HUIDHSI

Based on: HUIDVIS, HUIDHER, HUIDSPE, HUIDMOB, HUIDDEX, HUIDEMO, HUIDCOG, HUPDPAD

Description: This derived variable is a Health Utilties Index which provides a description of an individual's overall functional health, based on eight attributes: vision, hearing, speech, ambulation (ability to get around), dexterity (use of hands and fingers), emotion (feelings), cognition (memory and thinking) and pain. The version of the index used in CCHS is adapted from the HUI Mark 3 (HUI3). The index is designed to produce both an overall health utility score and eight individual attribute scores. Analysts can use either a single-attribute utility scale or look at the complete health state (levels on all eight attributes) on the overall utility scale to produce a measure of an individual's perceived health related quality of life (HRQL).

The index is appropriate for use to describe and monitor the health of general populations, and has been extensively validated for use in cross-sectional and longitudinal population health studies.

The 8 single-attribute utility scores measure functional capacity within a single attribute, and range from 1.00 (normal) to 0.00 (most disabled). In combination, these scores are used to produce a multi-attribute utility index producing a score ranging from 1.00 (perfect health), through 0.00 (health status equal to death) to -0.36 (health status worse than death).

Note: HUI3 question content resides in the public domain, and is not subject to copyright restrictions. The HUI3 algorithm is the property of Health Utilities Inc. and is protected by copyright. Statistics Canada is authorized, when requested, to share this algorithm with users who wish to replicate results or analyses conducted by Statistics Canada. The use of the algorithm for other purposes, or the sharing of it with others, is prohibited.

Higher scale indicates better health index
Range: -0.360 to 1 in increments of 0.001

Reference: For a detailed explanation of the calculation of the HUI3 refer to:

- Feeny D, Furlong W, Torrance GW et al. Multiattribute and single-attribute utility functions for the Health Utilities Index Mark 3 system. Med Care 2002; 40: 113-128.

Health utilities index - Pain and discomfort (1 DV)

The Health Utilties Index (HUI) is a multi-attribute health status classification system for measuring generic health status and health-related quality of life. The version used by CCHS is the HUI Mark 3 (HUI3), developed in Canada at McMaster University by Health Utilities Inc. The HUI3 allows the calculation of a generic health status index based on attributes found in two different CCHS modules - Health utilities index - Pain and discomfort (HUP) and the Health utilities index (HUI). HUIDHSI can only be calculated when both HUP and HUI are collected in a given cycle. For more information see "Feeny D, Furlong W, Torrance GW et al. Multi-attribute and single-attribute utility functions for the Health Utilities Index Mark 3 system. Med Care 2002; 40: 113-128."

1) Pain Health Status

Variable name: HUPDPAD

Based on: HUP_01, HUP_03

Description: Pain health status refers to the degree of pain that is usually felt by a person. This concept also considers whether this pain prevents him or her from performing certain activities. This variable is one of the 8 attributes used to calculate the Health Utility Index (HUIDHSI).

Specifications

Value	Condition(s)	Description	Notes
1	HUP_01 = 1 and<br>HUP_03 = 6	No pain or discomfort	
2	HUP_01 = 2 and<br>HUP_03 = 1	Pain - does not prevent activity	
3	HUP_01 = 2 and<br>HUP_03 = 2	Pain prevents a few activities	
4	HUP_01 = 2 and<br>HUP_03 = 3	Pain prevents some activities	
5	HUP_01 = 2 and<br>HUP_03 = 4	Pain prevents most activities	
9	(HUP_01 = DK, R, NS) or<br>(HUP_03 = DK, R, NS)	At least one required question was not answered (don't know, refusal, not stated)	NS

Reference: Reference: For more information on the Health Utilities Index and more details on each category please see http://www.statcan.gc.ca/subjects-sujets/standard-norme/otherclass-subject-autreclass-sujet-eng.htm.

Height and weight - Self-reported (5 DVs)

1) Height (Metres) - Self-Reported - Grouped

Variable name: HWTGHTM

Based on: HWT_2, HWT_2C, HWT_2D, HWT_2E, HWT_2F

Description: This variable indicates the height of the respondent in metres.

Note: For example, an individual who is 5 feet and 8 inches will have a height of 1.727 metres. The 1.727 is the midpoint of the range (1.715-1.739) around the height 5 feet and 8 inches. The range values were calculated as follows for an individual who is 5’8"

Specifications

Value	Condition(s)	Description	Notes
9.999	(HWT_2 = DK, R, NS) or<br>(HWT_2C = DK, R, NS) or<br>(HWT_2D = DK, R, NS) or<br>(HWT_2E = DK, R, NS) or<br>(HWT_2F = DK, R, NS)	At least one required question was not answered (don’t know, refusal, not stated)	NS
0.914	HWT_2 = 3 and<br>HWT_2C = 0	0.926 metres or shorter	
0.940	HWT_2 = 3 and<br>HWT_2C = 1	0.927 to 0.952 metres	
0.965	HWT_2 = 3 and<br>HWT_2C = 2	0.953 to 0.977 metres	
0.991	HWT_2 = 3 and<br>HWT_2C = 3	0.978 to 1.002 metres	
1.016	HWT_2 = 3 and<br>HWT_2C = 4	1.003 to 1.028 metres	
1.041	HWT_2 = 3 and<br>HWT_2C = 5	1.029 to 1.053 metres	
1.067	HWT_2 = 3 and<br>HWT_2C = 6	1.054 to 1.079 metres	
1.092	HWT_2 = 3 and<br>HWT_2C = 7	1.080 to 1.104 metres	
1.118	HWT_2 = 3 and<br>HWT_2C = 8	1.105 to 1.129 metres	
1.143	HWT_2 = 3 and<br>HWT_2C = 9	1.130 to 1.155 metres	
1.168	HWT_2 = 3 and<br>HWT_2C = 10	1.156 to 1.180 metres	
1.194	HWT_2 = 3 and<br>HWT_2C = 11	1.181 to 1.206 metres	
1.219	HWT_2 = 4 and<br>HWT_2D = 0	1.207 to 1.231 metres	
1.245	HWT_2 = 4 and<br>HWT_2D = 1	1.232 to 1.256 metres	
1.270	HWT_2 = 4 and<br>HWT_2D = 2	1.257 to 1.282 metres	
1.295	HWT_2 = 4 and<br>HWT_2D = 3	1.283 to 1.307 metres	

1.321	HWT_2 = 4 and<br>HWT_2D = 4	1.308 to 1.333 metres
1.346	HWT_2 = 4 and<br>HWT_2D = 5	1.334 to 1.358 metres
1.372	HWT_2 = 4 and<br>HWT_2D = 6	1.359 to 1.383 metres
1.397	HWT_2 = 4 and<br>HWT_2D = 7	1.384 to 1.409 metres
1.422	HWT_2 = 4 and<br>HWT_2D = 8	1.410 to 1.434 metres
1.448	HWT_2 = 4 and<br>HWT_2D = 9	1.435 to 1.460 metres
1.473	HWT_2 = 4 and<br>HWT_2D = 10	1.461 to 1.485 metres
1.499	HWT_2 = 4 and<br>HWT_2D = 11	1.486 to 1.510 metres
1.524	HWT_2 = 5 and<br>HWT_2E = 0	1.511 to 1.536 metres
1.549	HWT_2 = 5 and<br>HWT_2E = 1	1.537 to 1.561 metres
1.575	HWTE_2 = 5 and<br>HWT_2E = 2	1.562 to 1.587 metres
1.600	HWT_2 = 5 and<br>HWT_2E = 3	1.588 to 1.612 metres
1.626	HWT_2 = 5 and<br>HWT_2E = 4	1.613 to 1.637 metres
1.651	HWT_2 = 5 and<br>HWT_2E = 5	1.638 to 1.663 metres
1.676	HWT_2 = 5 and<br>HWT_2E = 6	1.664 to 1.688 metres
1.702	HWT_2 = 5 and<br>HWT_2E = 7	1.689 to 1.714 metres
1.727	HWT_2 = 5 and<br>HWT_2E = 8	1.715 to 1.739 metres
1.753	HWT_2 = 5 and<br>HWT_2E = 9	1.740 to 1.764 metres
1.778	HWT_2 = 5 and<br>HWT_2E = 10	1.765 to 1.790 metres
1.803	HWT_2 = 5 and<br>HWT_2E = 11	1.791 to 1.815 metres
1.829	HWT_2 = 6 and<br>HWT_2F = 0	1.816 to 1.841 metres
1.854	HWT_2 = 6 and<br>HWT_2F = 1	1.842 to 1.866 metres
1.880	HWT_2 = 6 and<br>HWT_2F = 2	1.867 to 1.891 metres
1.905	HWT_2 = 6 and<br>HWT_2F = 3	1.892 to 1.917 metres
1.930	HWT_2 = 6 and<br>HWT_2F = 4	1.918 to 1.942 metres
1.956	HWT_2 = 6 and<br>HWT_2F = 5	1.943 to 1.968 metres

1.981	HWT_2 = 6 and HWT_2F = 6	1.969 to 1.993 metres	
2.007	HWT_2 = 6 and HWT_2F = 7	1.994 to 2.018 metres	
2.032	HWT_2 = 6 and HWT_2F = 8	2.019 to 2.044 metres	
2.057	HWT_2 = 6 and HWT_2F = 9	2.045 to 2.069 metres	
2.083	HWT_2 = 6 and HWT_2F = 10	2.070 to 2.095 metres	
2.108	HWT_2 = 6 and HWT_2F = 11	2.096 to 2.120 metres	
2.134	HWT_2 = 7	2.121 metres or taller	

2) Weight (kilograms - grouped)

Variable name: HWTGWTK

Based on: HWT_3, HWT_N4

Description: The following variable describes the weight of the respondent in kilograms.

Note: Some values have been grouped as specified below.

Temporary Reformat

Value	Condition(s)	Description	Notes
**HWTA**			
HWTA_Q3 x .45	HW_N4 = 1	Weight is in pounds, convert to kilograms	
HWTA_Q3	HW_N4 = 2	Weight already in kilograms	

Specifications

Value	Condition(s)	Description	Notes
NS	(HWT_3 = DK, R or NS)	Respondent did not answer (don’t know, refusal, not stated)	
999.99	(HWT_3 = DK, R, NS)	Required question was not answered (don’t know, refusal, not stated)	NS
27	DHH_SEX = 1 and DHH_AGE => 12 and DHH_AGE <=14 and HWTDWTK <= 27	Male 12-14 < = 27	
HWT_3	HWT_N4 = 2	Weight in Kg.	
29	DHH_SEX = 2 and DHH_AGE => 12 and DHH_AGE <=14 and HWTDWTK <= 29	Female 12-14 < = 29	
HWT_3 × .45	HWT_N4 = 1	Weight in Kg., converted from Lbs.	
40	DHH_SEX = 2 and DHH_AGE => 15 and HWTDWTK <= 40	Female >15 < = 40	
41	DHH_SEX = 1 and DHH_AGE => 15 and DHH_AGE <=19 and HWTDWTK <= 41	Male 15-19 < = 41	
50	DHH_SEX = 1 and DHH_AGE => 20 and HWTDWTK <= 50	Male =>20 <=50	

86	DHH_SEX = 2 and DHH_AGE => 12 and DHH_AGE <= 14 and HWTDWTK => 86	Female 12-14 = >86
106	DHH_SEX = 1 and DHH_AGE => 12 and DHH_AGE <=14 and HWTDWTK => 106	Male 12-14 = >106
113	DHH_SEX = 2 and DHH_AGE => 15 and HWTDWTK => 113	Female > 15 = >113
130	DHH_SEX = 1 and DHH_AGE => 15 and DHH_AGE <= 19 and HWTDWTK >= 130	Male 15-19 = >130
137	DHH_SEX = 1 and DHH_AGE => 20 and HWTDWTK => 137	Male = > 20 = >137

3) Body mass index - grouped

Variable name: HWTGBMI

Based on: HWTGHTM, HWTGWTK

Description: The body mass index (BMI) is calculated for persons 20 to 64 years old, excluding pregnant women. BMI values have been regrouped to a minimum of 14 and a maximum of 58.

Note: BMI = WEIGHT (KG) / SQUARED HEIGHT (METRES)

Specifications

Value	Condition(s)	Description	Notes
NA	DHH_AGE < 20 or > 64	Respondent less than 20 or more than 64 years old	
NA	MAM_037 = 1	Respondent is pregnant	
NS	(HWTDHTM = NS) or HWTGWTK = NS	Height and/or weight was not given	
HWTGWTK / (HWTDHTM × HWTDHTM)	(HWTDHTM >= .914 and <= 2.108) and (HWTGWTK > 0 and <= 260)	BMI calculated from height and weight values	(Rounded to one decimal place) Min: 14; Max: 58

4) BMI classification for adults aged 18 and over (self reported) - international standard - grouped

Variable name: HWTGISW

Based on: HWTDBMI, DDH_AGE

Description: This variable assigns adult respondents aged 18 and over (except pregnant women) to one of the following categories, according to their Body Mass Index (BMI): underweight; acceptable weight; overweight; obese class I; obese class II; and, obese class III.

Note: According to Health Canada, this BMI classification system can be used as a screening tool to identify weight-related health risks at the population and individual levels. The following health risks are associated with each of the BMI categories for adult

Internet site: http://www.hc-sc.gc.ca/hpfb-dgpsa/onpp-bppn/weight_book_e.pdf

Specifications

Value	Condition(s)	Description	Notes
96	DDH_AGE < 18 or MAM_037 = 1	Population exclusions	NA

99	HWTDBMI = NS or<br>MAM_037 = DK, R, NS	At least one required question was not answered (don’t know, refusal, not stated)	NS
1	HWTDBMI < 18.50	Underweight	
2	(18.50 <= HWTDBMI <= 24.99)	Normal weight	
3	(25.00 <= HWTDBMI <= 29.99)	Overweight	
4	30.00 <= HWTDBMI	Obese - Class I, II, III	

Reference: For more detailed information see Canadian Guidelines for Body Weight Classification in Adults, Health Canada, 2003

5) BMI classification for children aged 12 to 17 (self-reported) - Cole classification system

Variable name: HWTDCOL

Based on: HWTDBMI, DHH_SEX, DHHYOB, DHHMOB, DHHDOB, ADM_YOI, ADM_MOI, ADM_DOI

Description: This variable classifies children aged 12 to 17 (except female respondents aged 15 to 17 who were pregnant or did not answer the pregnancy question) as "obese", "overweight" or "neither obese nor overweight" according to the age-and-sex-specific BMI cut-off points as defined by Cole et al. The Cole cut-off points are based on pooled international data (Brazil, Great Britain, Hong Kong, Netherlands, Singapore, and United States) for BMI and linked to the widely internationally accepted adult BMI cut-off points of 25 (overweight) and 30 (obese).

Note: Respondents who do not fall within the categories of "Obese" or "Overweight" (as defined by Cole et al.) have been classified by CCHS as "neither obese nor overweight".

This variable excludes respondents who are 18 years old or over (216 months).

Temporary Reformat

Value	Condition(s)	Description	Notes
**AGET1**			
DHH_AGM / 12	DHH_AGM < 9996	Convert respondent’s "age in months" to "age in years"	(Rounded to nearest 0.5)
**DHH_AGM**			
9999	(DHH_DOB = DK, R, NS) or (DHH_MOB = DK, R or NS) or (DHH_YOB = DK, R or NS)	A valid day of birth or month of birth or year of birth is not available for the respondent.	NS
Age in months	Interview date converted in months (ADM_YOI, ADM_MOI and ADM_DOI) - Date of birth converted in months (DHH_YOB, DHH_MOB and DHH_DOB)	Create respondent’s age in months at time of the interview	(min:144; max:1224)

Specifications

Value	Condition(s)	Description	Notes
6	MAM_037 = 1 or<br>(17 < DHH_AGE or DHH_AGE < 12) or<br>(DHH_AGM >= 216 and<br>DHH_AGM < 9999)	Population exclusion	NA
9	HWTDBMI = NS or<br>(MAM_037 = DK, R, NS) or<br>DHH_AGM = NS	At least one required question was not answered (don’t know, refusal, not stated)	NS

3	(AGET1 = 12 and<br>DHH_SEX = 1 and<br>999.96 > HWTDBMI >= 26.02) or<br>(AGET1 = 12 and<br>DHH_SEX = 2 and<br>999.96 > HWTDBMI >= 26.67) or<br>(AGET1 = 12.5 and<br>DHH_SEX = 1 and<br>999.96 > HWTDBMI >= 26.43) or<br>(AGET1 = 12.5 and<br>DHH_SEX = 2 and<br>999.96 > HWTDBMI >= 27.24) or<br>(AGET1 = 13 and<br>DHH_SEX = 1 and<br>999.96 > HWTDBMI >= 26.84) or<br>(AGET1 = 13 and<br>DHH_SEX = 2 and<br>999.96 > HWTDBMI >= 27.76) or<br>(AGET1 = 13.5 and<br>DHH_SEX = 1 and<br>999.96 > HWTDBMI >= 27.25) or<br>(AGET1 = 13.5 and<br>DHH_SEX = 2 and<br>999.96 > HWTDBMI >= 28.20) or<br>(AGET1 = 14 and<br>DHH_SEX = 1 and<br>999.96 > HWTDBMI >= 27.63) or<br>(AGET1 = 14 and<br>DHH_SEX = 2 and<br>999.96 > HWTDBMI >= 28.57) or<br>(AGET1 = 14.5 and<br>DHH_SEX = 1 and<br>999.96 > HWTDBMI >= 27.98) or<br>(AGET1 = 14.5 and<br>DHH_SEX = 2 and<br>999.96 > HWTDBMI >= 28.87) or<br>(AGET1 = 15 and<br>DHH_SEX = 1 and<br>999.96 > HWTDBMI >= 28.30) or<br>(AGET1 = 15 and<br>DHH_SEX = 2 and<br>999.96 > HWTDBMI >= 29.11) or<br>(AGET1 = 15.5 and<br>DHH_SEX = 1 and<br>999.96 > HWTDBMI >= 28.60) or<br>(AGET1 = 15.5 and<br>DHH_SEX = 2 and<br>999.96 > HWTDBMI >= 29.29) or<br>(AGET1 = 16 and<br>DHH_SEX = 1 and<br>999.96 > HWTDBMI >= 28.88) or<br>(AGET1 = 16 and<br>DHH_SEX = 2 and<br>999.96 > HWTDBMI >= 29.43) or<br>(AGET1 = 16.5 and<br>DHH_SEX = 1 and<br>999.96 > HWTDBMI >= 29.14) or<br>(AGET1 = 16.5 and<br>DHH_SEX = 2 and<br>999.96 > HWTDBMI >= 29.56) or<br>(AGET1 = 17 and<br>DHH_SEX = 1 and<br>999.96 > HWTDBMI >= 29.41) or<br>(AGET1 = 17 and<br>DHH_SEX = 2 and<br>999.96 > HWTDBMI >= 29.69) or<br>(AGET1 = 17.5 and<br>DHH_SEX = 1 and<br>999.96 > HWTDBMI >= 29.70) or<br>(AGET1 = 17.5 and<br>DHH_SEX = 2 and<br>999.96 > HWTDBMI >= 29.84) or<br>(AGET1 = 18 and	Obese

	DHH_SEX = 1 and<br>999.96 > HWTDBMI >= 30.00) or<br>(AGET1 = 18 and<br>DHH_SEX = 2 and<br>999.96 > HWTDBMI >= 30.00)

2	(AGET1 = 12 and<br>DHH_SEX = 1 and<br>(21.22 <= HWTDBMI < 26.02)) or<br>(AGET1 = 12 and<br>DHH_SEX = 2 and<br>(21.68 <= HWTDBMI < 26.67)) or<br>(AGET1 = 12.5 and<br>DHH_SEX = 1 and<br>(21.56 <= HWTDBMI < 26.43)) or<br>(AGET1 = 12.5 and<br>DHH_SEX = 2 and<br>(22.14 <= HWTDBMI < 27.24)) or<br>(AGET1 = 13 and<br>DHH_SEX = 1 and<br>(21.91 <= HWTDBMI < 26.84)) or<br>(AGET1 = 13 and<br>DHH_SEX = 2 and<br>(22.58 <= HWTDBMI < 27.76)) or<br>(AGET1 = 13.5 and<br>DHH_SEX = 1 and<br>(22.27 <= HWTDBMI < 27.25)) or<br>(AGET1 = 13.5 and<br>DHH_SEX = 2 and<br>(22.98 <= HWTDBMI < 28.20)) or<br>(AGET1 = 14 and<br>DHH_SEX = 1 and<br>(22.62 <= HWTDBMI < 27.63)) or<br>(AGET1 = 14 and<br>DHH_SEX = 2 and<br>(23.34 <= HWTDBMI < 28.57)) or<br>(AGET1 = 14.5 and<br>DHH_SEX = 1 and<br>(22.96 <= HWTDBMI < 27.98)) or<br>(AGET1 = 14.5 and<br>DHH_SEX = 2 and<br>(23.66 <= HWTDBMI < 28.87)) or<br>(AGET1 = 15 and<br>DHH_SEX = 1 and<br>(23.29 <= HWTDBMI < 28.30)) or<br>(AGET1 = 15 and<br>DHH_SEX = 2 and<br>(23.94 <= HWTDBMI < 29.11)) or<br>(AGET1 = 15.5 and<br>DHH_SEX = 1 and<br>(23.60 <= HWTDBMI < 28.60)) or<br>(AGET1 = 15.5 and<br>DHH_SEX = 2 and<br>(24.17 <= HWTDBMI < 29.29)) or<br>(AGET1 = 16 and<br>DHH_SEX = 1 and<br>(23.90 <= HWTDBMI < 28.88)) or<br>(AGET1 = 16 and<br>DHH_SEX = 2 and<br>(24.37 <= HWTDBMI < 29.43)) or<br>(AGET1 = 16.5 and<br>DHH_SEX = 1 and<br>(24.19 <= HWTDBMI < 29.14)) or<br>(AGET1 = 16.5 and<br>DHH_SEX = 2 and<br>(24.54 <= HWTDBMI < 29.56)) or<br>(AGET1 = 17 and<br>DHH_SEX = 1 and<br>(24.46 <= HWTDBMI < 29.41)) or<br>(AGET1 = 17 and<br>DHH_SEX = 2 and<br>(24.70 <= HWTDBMI < 29.69)) or<br>(AGET1 = 17.5 and<br>DHH_SEX = 1 and<br>(24.73 <= HWTDBMI < 29.70)) or<br>(AGET1 = 17.5 and<br>DHH_SEX = 2 and<br>(24.85 <= HWTDBMI < 29.84)) or<br>(AGET1 = 18 and	Overweight

	DHH_SEX = 1 and<br>(25.00 <= HWTDBMI < 30.00)) or<br>(AGET1 = 18 and<br>DHH_SEX = 2 and<br>(25.00 <= HWTDBMI < 30.00))	
1	Else	Neither overweight nor obese

Reference: For more information about the Cole BMI classification system, see Establishing a Standard Definition for Child Overweight and Obesity Worldwide - International survey, by Tim J Cole, Mary C Bellizzi, Katherine M. Flegal, William H Dietz, published in British Medical Journal, Volume: 320, May 2000.

Illicit drug use (9 DVs)

This module assesses use of various illicit drugs and drug interference. The questions for drug use are based on Canada's Alcohol and Other Drugs Survey (1994). Interference in daily activities and responsibilities is assessed.

1) Cannabis Drug Use - Lifetime (Including "One Time Only" Use)

Variable name: IDGFLCA

Based on: IDG_01

Description: This variable indicates whether respondents have ever used marijuana, cannabis or hashish.

Source: Canada's Alcohol and Other Drugs Survey (1994)

Specifications

Value	Condition(s)	Description	Notes
6	DOIDG = 2	Module not selected	NA
9	ADM_PRX = 1	Module not asked - proxy interview	NS
1	(IDG_01 = 1, 2)	Has used marijuana	
2	IDG_01 = 3	Has never used marijuana	
9	(IDG_01 = DK, R, NS)	At least one required question was not answered (don't know, refusal, not stated)	NS

2) Cannabis Drug Use - Lifetime (Excluding "One Time Only" Use)

Variable name: IDGFLCM

Based on: IDG_01

Description: This variable indicates whether respondents have used marijuana, cannabis or hashish more than just once.

Source: Canada's Alcohol and Other Drugs Survey (1994)

Specifications

Value	Condition(s)	Description	Notes
6	DOIDG = 2	Module not selected	NA
9	ADM_PRX = 1	Module not asked - proxy interview	NS
1	IDG_01 = 2	Has used marijuana more than once	
2	(IDG_01 = 1, 3)	Has not used marijuana more than once	
9	(IDG_01 = DK, R, NS)	The required question was not answered (don't know, refusal, not stated)	NS

3) Cannabis Drug Use - 12 month (Excluding "One Time Only" Use)

Variable name: IDGFYCM

Based on: IDG_01, IDG_02

Description: This variable indicates whether respondents have used marijuana, cannabis or hashish in the past year, excluding one time use in lifetime.

Source: Canada’s Alcohol and Other Drugs Survey (1994)

Specifications

Value	Condition(s)	Description	Notes
6	DOIDG = 2	Module not selected	NA
9	ADM_PRX = 1	Module not asked - proxy interview	NS
1	IDG_01 = 2 and<br>IDG_02 = 1	Has used marijuana in the past 12 months and has used marijuana more than once in his/her lifetime	
2	(IDG_01 = 1 and<br>IDG_02 = 1) or<br>(IDG_02 = 2, NA)	Has not used marijuana in the past 12 months or used it once in the past 12 months and this was the only lifetime use	
9	(IDG_02 = DK, R, NS)	At least one required question was not answered (don’t know, refusal, not stated)	NS

4) Any Illicit Drug Use - Lifetime (Including "One Time Only" Use of Cannabis)

Variable name: IDGFLA

Based on: IDGFLCA, IDGFLCO, IDGFLAM, IDGFLEX, IDGFLHA, IDGFLGL, IDGFLHE, IDGFLST

Description: This variable indicates whether respondents have ever used any of the drugs listed. Includes one time use of cannabis.

Source: Canada’s Alcohol and Other Drugs Survey (1994)

Specifications

Value	Condition(s)	Description	Notes
6	DOIDG = 2	Module not selected	NA
9	ADM_PRX = 1	Module not asked - proxy interview	NS
1	IDGFLCA = 1 or<br>IDGFLCO = 1 or<br>IDGFLAM = 1 or<br>IDGFLEX = 1 or<br>IDGFLHA = 1 or<br>IDGFLGL = 1 or<br>IDGFLHE = 1 or<br>IDGFLST = 1	Has used at least 1 of 8 drugs if lifetime, including "one time only" use of cannabis	
2	IDGFLCA = 2 and<br>IDGFLCO = 2 and<br>IDGFLAM = 2 and<br>IDGFLEX = 2 and<br>IDGFLHA = 2 and<br>IDGFLGL = 2 and<br>IDGFLHE = 2 and<br>IDGFLST = 2	Has never used drugs listed	
9	IDGFLCA = NS or<br>IDGFLCO = NS or<br>IDGFLAM = NS or<br>IDGFLEX = NS or<br>IDGFLHA = NS or<br>IDGFLGL = NS or<br>IDGFLHE = NS or<br>IDGFLST = NS	At least one required question was not answered (don’t know, refusal, not stated)	NS

5) Any Illicit Drug Use - Lifetime (Excluding "One Time Only" Use of Cannabis)

Variable name: IDGFLAC

Based on: IDGFLCM, IDGFLCO, IDGFLAM, IDGFLEX, IDGFLHA, IDGFLGL, IDGFLHE, IDGFLST

Description: This variable indicates whether respondents have ever used any of the drugs listed. Excludes one time use of cannabis.

Source: Canada's Alcohol and Other Drugs Survey (1994)

Specifications

Value	Condition(s)	Description	Notes
6	DOIDG = 2	Module not selected	NA
9	ADM_PRX = 1	Module not asked - proxy interview	NS
1	IDGFLCM = 1 or<br>IDGFLCO = 1 or<br>IDGFLAM = 1 or<br>IDGFLEX = 1 or<br>IDGFLHA = 1 or<br>IDGFLGL = 1 or<br>IDGFLHE = 1 or<br>IDGFLST = 1	Has used at least 1 of 8 drugs, excluding "one time only" use of cannabis	
2	IDGFLCM = 2 and<br>IDGFLCO = 2 and<br>IDGFLAM = 2 and<br>IDGFLEX = 2 and<br>IDGFLHA = 2 and<br>IDGFLGL = 2 and<br>IDGFLHE = 2 and<br>IDGFLST = 2	Has never used drugs listed, excluding one time use of cannabis	
9	IDGFLCM = NS or<br>IDGFLCO = NS or<br>IDGFLAM = NS or<br>IDGFLEX = NS or<br>IDGFLHA = NS or<br>IDGFLGL = NS or<br>IDGFLHE = NS or<br>IDGFLST = NS	At least one required question was not answered (don't know, refusal, not stated)	NS

6) Any Illicit Drug Use - 12-Month (Including "One Time Only" Use of Cannabis)

Variable name: IDGFYA

Based on: IDG_02, IDG_05, IDG_08, IDG_11, IDG_14, IDG_17, IDG_20, IDG_23

Description: This variable indicates whether respondents used any of the drugs listed in the past 12 months. Includes one time use of cannabis.

Source: Canada's Alcohol and Other Drugs Survey (1994)

Specifications

Value	Condition(s)	Description	Notes
6	DOIDG = 2	Module not selected	NA
9	ADM_PRX = 1	Module not asked - proxy interview	NS

1	IDG_02 = 1 or<br>IDG_05 = 1 or<br>IDG_08 = 1 or<br>IDG_11 = 1 or<br>IDG_14 = 1 or<br>IDG_17 = 1 or<br>IDG_20 = 1 or<br>IDG_23 = 1	Has used at least 1 of 8 drugs listed in the past 12 months, including "one time only" use of cannabis	
2	(IDG_02 = 2, NA) and<br>(IDG_05 = 2, NA) and<br>(IDG_08 = 2, NA) and<br>(IDG_11 = 2, NA) and<br>(IDG_14 = 2, NA) and<br>(IDG_17 = 2, NA) and<br>(IDG_20 = 2, NA) and<br>(IDG_23 = 2, NA)	Has not used drugs listed in the past 12 months	
9	(IDG_02 = DK, R, NS) or<br>(IDG_05 = DK, R, NS) or<br>(IDG_08 = DK, R, NS) or<br>(IDG_11 = DK, R, NS) or<br>(IDG_14 = DK, R, NS) or<br>(IDG_17 = DK, R, NS) or<br>(IDG_20 = DK, R, NS) or<br>(IDG_23 = DK, R, NS)	At least one required question was not answered (don’t know, refusal, not stated)	NS

7) Any Illicit Drug Use - 12-Month (Excluding "One Time Only" Use of Cannabis)

Variable name: IDGFYAC

Based on: IDGFYCM, IDG_05, IDG_08, IDG_11, IDG_14, IDG_17, IDG_20, IDG_23

Description: This variable indicates whether respondents used any of the drugs listed in the past 12 months. Excludes one time use of cannabis.

Source: Canada’s Alcohol and Other Drugs Survey (1994)

Specifications

Value	Condition(s)	Description	Notes
6	DOIDG = 2	Module not selected	NA
9	ADM_PRX = 1	Module not asked - proxy interview	NS
1	IDGFYCM = 1 or<br>IDG_05 = 1 or<br>IDG_08 = 1 or<br>IDG_11 = 1 or<br>IDG_14 = 1 or<br>IDG_17 = 1 or<br>IDG_20 = 1 or<br>IDG_23 = 1	Has used at least 1 of 8 drugs listed in the past 12 months, excluding "one time only" lifetime use of cannabis	
2	IDGFYCM = 2 and<br>(IDG_05 = 2, NA) and<br>(IDG_08 = 2, NA) and<br>(IDG_11 = 2, NA) and<br>(IDG_14 = 2, NA) and<br>(IDG_17 = 2, NA) and<br>(IDG_20 = 2, NA) and<br>(IDG_23 = 2, NA)	Has not used drugs listed in the past 12 months, excluding "one time only" lifetime use of cannabis	

9	IDGFYCM = NS or (IDG_05 = DK, R, NS) or (IDG_08 = DK, R, NS) or (IDG_11 = DK, R, NS) or (IDG_14 = DK, R, NS) or (IDG_17 = DK, R, NS) or (IDG_20 = DK, R, NS) or (IDG_23 = DK, R, NS)	At least one required question was not answered (don't know, refusal, not stated)	NS

8) Illicit Drug Interference 12-Month - Mean

Variable name: IDGDINT

Based on: IDG_26A, IDG_6B1, IDG_6B2, IDG_26C, IDG_26D

Description: This variable assesses the interference that drug use had on daily activities and responsibilities in the past 12 months. It is a mean of the 5 items.

Note: Respondents who did not use drugs frequently enough or did not indicate problems with drug use were excluded from the population.

Specifications

Value	Condition(s)	Description	Notes
99.6	DOIDG = 2	Module not selected	NA
99.6	IDG_26A = NA	Population exclusions	NA
99.9	ADM_PRX = 1	Module not asked - proxy interview	NS
99.9	(IDG_26A = DK, R, NS) or (IDG_6B1 = DK, R, NS) or (IDG_6B2 = DK, R, NS) or (IDG_26C = DK, R, NS) or (IDG_26D = DK, R, NS)	At least one required question was not answered (don't know, refusal, not stated)	NS
(IDG_26A + IDG_6B1 + IDG_6B2 + IDG_26C + IDG_26D) / 5	(0 <= IDG_26A <= 10) and (0 <= IDG_6B1 <= 10) and (0 <= IDG_6B2 <= 10) and (0 <= IDG_26C <= 10) and (0 <= IDG_26D <= 10)	Interference = mean of all 5 items. Answered all 5 questions	(Rounded to one decimal place) (min: 0.0; max: 10.0)
(IDG_26A + IDG_6B2 + IDG_26C + IDG_26D) / 4	IDG_6B1 = 11 and (0 <= IDG_6B2 <= 10) and (0 <= IDG_26A <= 10) and (0 <= IDG_26C <= 10) and (0 <= IDG_26D <= 10)	Interference = mean of 4 items that applied IDG_6B1 was not applicable	(Rounded to one decimal place) (min: 0.0; max: 10.0)
(IDG_26A + IDG_6B1 + IDG_26C + IDG_26D) / 4	(0 <= IDG_6B1 <= 10) and IDG_6B2 = 11 and (0 <= IDG_26A <= 10) and (0 <= IDG_26C <= 10) and (0 <= IDG_26D <= 10)	Interference = mean of 4 items that applied IDG_6B2 was not applicable	(Rounded to one decimal place) (min: 0.0; max: 10.0)
(IDG_26A + IDG_26C + IDG_26D) / 3	IDG_6B1= 11 and IDG_6B2 = 11 and (0 <= IDG_26A <= 10) and (0 <= IDG_26C <= 10) and (0 <= IDG_26D <= 10)	Interference = mean of 3 items that applied IDG_6B1 and IDG_6B2 were not applicable	(Rounded to one decimal place) (min: 0.0; max: 10.0)

9) Flag for Illicit Drug Interference - 12-Month

Variable name: IDGFINT

Based on: IDG_26A, IDG_6B1, IDG_6B2, IDG_26C, IDG_26D

Description: This variable assesses the interference that drug use had on daily activities and responsibilities in the past 12 months. This is a classification that indicates whether drug use interferes significantly with the person's normal routine, occupational (academic) functioning, or social activities or relationships.

Note: Respondents who did not use drugs frequently enough or did not indicate problems with drug use where excluded from the population.

Specifications

Value	Condition(s)	Description	Notes
6	DOIDG = 2	Module not selected	NA
6	IDG_26A = NA	Population exclusions	NA
9	ADM_PRX = 1	Module not asked - proxy interview	NS
1	(4 <= IDG_26A <= 10) or<br>(4 <= IDG_6B1 <= 10) or<br>(4 <= IDG_6B2 <= 10) or<br>(4 <= IDG_26C <= 10) or<br>(4 <= IDG_26D <= 10)	Drug use interfered significantly with normal routine, occupational (academic) functioning, or social activities or relationships in the past 12 months	
2	(0 <= IDG_26A <= 3) and<br>[(0 <= IDG_6B1 <= 3) or<br>IDG_6B1 = 11] and<br>[(0 <= IDG_6B2 <= 3) or<br>IDG_6B2 = 11] and<br>(0 <= IDG_26C <= 3) and<br>(0 <= IDG_26D <= 3)	Drug use did not interfere significantly with normal routine, occupation (academic) functioning or social activities or relationships in the past 12 months	
9	(IDG_26A = DK, R, NS) or<br>(IDG_6B1 = DK, R, NS) or<br>(IDG_6B2 = DK, R, NS) or<br>(IDG_26C = DK, R, NS) or<br>(IDG_26D = DK, R, NS)	At least one required question was not answered (don't know, refusal, not stated)	NS

Income (7 DVs)

TEMPORARY VARIABLE

Household income ratio

Variable name: INCTRAT

Based on: INC_5, INCDHH, GEO_PRV, DHHDHSZ, GEODPSZ

This derived variable is a temporary variable used in the calculation of adjusted ratios (INCDADR). While INCDADR is disseminated in the master and share files, INCTRAT is not. The Territories are excluded from this derived variable.

This derived variable is a ratio between the total income of the respondent's household and the low income cut-off corresponding to the number of persons in the household and the size of the community. The low income cut-off is the threshold at which a family would typically spend a larger portion of its income than the average family on the necessities of food, shelter and clothing.

This derived variable is produced in three separate steps. A summary of those steps is provided below.

Step 1: Low income cut-offs for each family and community size were obtained for the 2007 reference year from the Survey of Labour and Income Dynamics (SLID). In the case of CCHS, the income questions refer to the past 12 months. Although the survey data were collected in 2008, at the time the data was to be processed, 2007 was the most recent year for which low income cut-offs could be provided.

A low income cut-off was linked to all respondents (INCTLIC). This cut-off corresponded to the size of the respondent's household (DHHDHSZ) and the size of the community in which the respondent lives (GEODPSZ). Therefore, respondents were assigned one of the 35 possible combinations that exist (7 household size groups times 5 community size groups). For instance, the INCTLIC variable of a respondent living in a household size of 3 people and in an urban community with a population of 47,000 people would be 28,379.

Ref.: Low income cut-offs (INCTLIC) were taken from Table 3 in Low income cut-offs for 2007 and low income measures for 2006. Income Research Paper Series. Catalogue no. 75F0002M No. 004, June 2008.

Step 2a: Household income is obtained using INC_5 questions for a specific amount and INCDHH (INC_5A to INC_5C) for an amount in an interval.

If a specific amount is obtained at question INC_5, that amount is used as household income. If only one interval is reported for INC_5A to INC_5C, a random value within each interval is derived from INCDHH for household income for all intervals but the highest one (see next step).

Step 2b: For the highest household income interval ($100 000 or more), for each province, the median value from the Survey of Labour and Income Dynamics (SLID) for the same interval will be used as the household income. Data from SLID 2007 were used as they were the most recent available at the time CCHS data were processed.

Median provincial household income in 2007 from the SLID for the "100 000 $ or more" category are as follows:

	2007
Newfoundland and Labrador	142 580
Prince Edward Island	133 457
Nova Scotia	145 050
New Brunswick	139 659
Quebec	143 119
Ontario	153 360
Manitoba	149 934
Saskatchewan	145 987
Alberta	182 772
British Columbia	155 787

Step 3: Individual ratios of household income to the low income cut-off are calculated for each household within each household and community size using the DHHDHSZ household size variable and the GEODPSZ community size variable. Ratios are calculated by dividing household income (INCTINC) by the corresponding low income cut-off (INCTLIC).

Temporary Reformat

Value	Condition(s)	Description	Notes
**INCTINC**			
999996	GEO_PRV = 60, 61, 62	Residents of Territories excluded	
999999	INCDHH = 99	None of the income questions was stated	
0	INCDHH = 1	No income	
INC_3	0 < INC_3 < 999996	Specific and positive household income	

RANDOM (MIN=1, MAX=4999)	INCDHH = 2	Random variable for a stated income in an interval of $1 to $4,999
RANDOM (MIN=5000, MAX=9999)	INCDHH = 3	Random variable for a stated income in an interval of $5,000 to $9,999
RANDOM (MIN=10000, MAX=14999)	INCDHH = 4	Random variable for a stated income in an interval of $10,000 to $14,999
RANDOM (MIN=15000, MAX=19999)	INCDHH = 5	Random variable for a stated income in an interval of $15,000 to $19,999
RANDOM (MIN=20000, MAX=29999)	INCDHH = 6	Random variable for a stated income in an interval of $20,000 to $29,999
RANDOM (MIN=30000, MAX=39999)	INCDHH = 7	Random variable for a stated income in an interval of $30,000 to $39,999
RANDOM (MIN=40000, MAX=49999)	INCDHH = 8	Random variable for a stated income in an interval of $40,000 to $49,999
RANDOM (MIN=50000, MAX=59999)	INCDHH = 9	Random variable for a stated income in an interval of $50,000 to $59,999
RANDOM (MIN=60000, MAX=69999)	INCDHH = 10	Random variable for a stated income in an interval of $60,000 to $69,999
RANDOM (MIN=70000, MAX=79999)	INCDHH = 11	Random variable for a stated income in an interval of $70,000 to $79,999
RANDOM (MIN=80000, MAX=89999)	INCDHH = 12	Random variable for a stated income in an interval of $80,000 to $89,999
RANDOM (MIN=90000, MAX=99999)	INCDHH = 13	Random variable for a stated income in an interval of $90,000 to $99,999
133,457	INCDHH = 14 and GEO_PRV = 11	Imputed value from SLID if the province of residence is Prince Edward Island and income > 100,000$
139,659	INCDHH = 14 and GEO_PRV = 13	Imputed value from SLID if the province of residence is New Brunswick and income > 100,000$
142,580	INCDHH = 14 and GEO_PRV = 10	Imputed value from SLID if the province of residence is Newfoundland and Labrador and income > 100,000$
143,119	INCDHH = 14 and GEO_PRV = 24	Imputed value from SLID if the province of residence is Quebec and income > 100,000$
149,934	INCDHH = 14 and GEO_PRV = 46	Imputed value from SLID if the province of residence is Manitoba and income > 100,000$
145,987	INCDHH = 14 and GEO_PRV = 47	Imputed value from SLID if the province of residence is Saskatchewan and income > 100,000$
155,787	INCDHH = 14 and GEO_PRV = 59	Imputed value from SLID if the province of residence is British Columbia and income > 100,000$
145,050	INCDHH = 14 and GEO_PRV = 12	Imputed value from SLID if the province of residence is Nova Scotia and income > 100,000$
153,360	INCDHH = 14 and GEO_PRV = 35	Imputed value from SLID if the province of residence is Ontario and income > 100,000$
182,772	INCDHH = 14 and GEO_PRV = 48	Imputed value from SLID if the province of residence is Alberta and income > 100,000$

INCTLIC

14 914	DHHDHSZ = 1 and GEODPSZ = 1	Low income cut-offs when the number of persons in household = 1 and population size group = rural area

16 968	DHHDHSZ = 1 and GEODPSZ = 2	Low income cut-offs when the number of persons in household = 1 and population size group = urban area - less than 30,000 people
18 544	DHHDHSZ = 1 and GEODPSZ = 3	Low income cut-offs when the number of persons in household = 1 and population size group = urban area - 30,000 to 99,999 people
18 567	DHHDHSZ = 2 and GEODPSZ = 1	Low income cut-offs when the number of persons in household = 2 and population size group = rural area
18 659	DHHDHSZ = 1 and GEODPSZ = 4	Low income cut-offs when the number of persons in household = 1 and population size group = urban area - 100,000 to 499,999 people
21 123	DHHDHSZ = 2 and GEODPSZ = 2	Low income cut-offs when the number of persons in household = 2 and population size group = urban area - less than 30,000 people
21 666	DHHDHSZ = 1 and GEODPSZ = 5	Low income cut-offs when the number of persons in household = 1 and population size group = urban area - 500,000 people or more
22 826	DHHDHSZ = 3 and GEODPSZ = 1	Low income cut-offs when the number of persons in household = 3 and population size group = rural area
23 084	DHHDHSZ = 2 and GEODPSZ = 3	Low income cut-offs when the number of persons in household = 2 and population size group = urban area - 30,000 to 99,999 people
23 228	DHHDHSZ = 2 and GEODPSZ = 4	Low income cut-offs when the number of persons in household = 2 and population size group = urban area - 100,000 to 499,999 people
25 968	DHHDHSZ = 3 and GEODPSZ = 2	Low income cut-offs when the number of persons in household = 3 and population size group = urban area - less than 30,000 people
26 972	DHHDHSZ = 2 and GEODPSZ = 5	Low income cut-offs when the number of persons in household = 2 and population size group = urban area - 500,000 people or more
27 714	DHHDHSZ = 4 and GEODPSZ = 1	Low income cut-offs when the number of persons in household = 4 and population size group = rural area
28 379	DHHDHSZ = 3 and GEODPSZ = 3	Low income cut-offs when the number of persons in household = 3 and population size group = urban area - 30,000 to 99,999 people
28 556	DHHDHSZ = 3 and GEODPSZ = 4	Low income cut-offs when the number of persons in household = 3 and population size group = urban area - 100,000 to 499,999 people
31 432	DHHDHSZ = 5 and GEODPSZ = 1	Low income cut-offs when the number of persons in household = 5 and population size group = rural area
31 529	DHHDHSZ = 4 and GEODPSZ = 2	Low income cut-offs when the number of persons in household = 4 and population size group = urban area - less than 30,000 people
33 159	DHHDHSZ = 3 and GEODPSZ = 5	Low income cut-offs when the number of persons in household = 3 and population size group = urban area - 500,000 people or more
34 457	DHHDHSZ = 4 and GEODPSZ = 3	Low income cut-offs when the number of persons in household = 4 and population size group = urban area - 30,000 to 99,999 people
34 671	DHHDHSZ = 4 and GEODPSZ = 4	Low income cut-offs when the number of persons in household = 4 and population size group = urban area - 100,000 to 499,999 people
35 452	DHHDHSZ = 6 and GEODPSZ = 1	Low income cut-offs when the number of persons in household = 6 and population size group = rural area
35 760	DHHDHSZ = 5 and GEODPSZ = 2	Low income cut-offs when the number of persons in household = 5 and population size group = urban area - less than 30,000 people
39 081	DHHDHSZ = 5 and GEODPSZ = 3	Low income cut-offs when the number of persons in household = 5 and population size group = urban area - 30,000 to 99,999 people
39 322	DHHDHSZ = 5 and GEODPSZ = 4	Low income cut-offs when the number of persons in household = 5 and population size group = urban area - 100,000 to 499,999 people

39 470	DHHDHSZ >= 7 and GEODPSZ = 1	Low income cut-offs when the number of persons in household >= 7 and population size group = rural area	
40 259	DHHDHSZ = 4 and GEODPSZ = 5	Low income cut-offs when the number of persons in household = 4 and population size group = urban area - 500,000 people or more	
40 331	DHHDHSZ = 6 and GEODPSZ = 2	Low income cut-offs when the number of persons in household = 6 and population size group = urban area - less than 30,000 people	
44 077	DHHDHSZ = 6 and GEODPSZ = 3	Low income cut-offs when the number of persons in household = 6 and population size group = urban area - 30,000 to 99,999 people	
44 350	DHHDHSZ = 6 and GEODPSZ = 4	Low income cut-offs when the number of persons in household = 6 and population size group = urban area - 100,000 to 499,999 people	
44 903	DHHDHSZ >= 7 and GEODPSZ = 2	Low income cut-offs when the number of persons in household >= 7 and population size group = urban area - less than 30,000 people	
45 662	DHHDHSZ = 5 and GEODPSZ = 5	Low income cut-offs when the number of persons in household = 5 and population size group = urban area - 500,000 people or more	
49 073	DHHDHSZ >= 7 and GEODPSZ = 3	Low income cut-offs when the number of persons in household >= 7 and population size group = urban area - 30,000 to 99,999 people	
49 377	DHHDHSZ >= 7 and GEODPSZ = 4	Low income cut-offs when the number of persons in household => 7 and population size group = urban area - 100,000 to 499,999 people	
51 498	DHHDHSZ = 6 and GEODPSZ = 5	Low income cut-offs when the number of persons in household = 6 and population size group = urban area - 500,000 people or more	
57 336	DHHDHSZ >= 7 and GEODPSZ = 5	Low income cut-offs when the number of persons in household >= 7 and population size group = urban area - 500,000 people or more	
**INCTRAT**			
99.999999996	INCTINC = 999996	Residents of territories excluded	9 decimals
99.999999999	INCTINC = 999999	The ratio cannot be calculated because the household income was not stated	9 decimals
0-40	INCTINC / INCTLIC	Individual ratio of household income to the low income cut-off corresponding to the size of the household and the size of the community. The maximum ratio is based on the maximum household income accepted, which is $500,000	9 decimals

1) Total household income - main source - Grouped

Variable name: INCG2

Based on: INC_2

Description: This variable groups the main source of total household income into four categories.

Note: Derived variable speficications were updated in 2009 due to changes in INC_2 answer categories.

Specifications

Value	Condition(s)	Description	Notes
9	(INC_2 = DK, R, NS)	Required question was not answered (don't know, refusal, not stated)	NS
1	(INC_2 = 1, 2)	Wages/salaries or self-employment	
2	(INC_2 = 4, 5, 10)	Employment insurance or worker's compensation or social assistance/welfare	

3	(INC_2 = 6, 7, 8, 9)	Benefits from Canada or Quebec Pension Plan or job related retirement pensions, superannuation and annuities or RRSP/RRIF of Old Age Security and Guaranteed Income Supplement	
4	(INC_2 = 3, 11, 12, 13, 14, 15)	Dividends/interest or child tax benefit or child support or alimony or other or no income	

2) Main source of personal income - Grouped

Variable name: INCG7

Based on: INC_7

Description: This variable groups the main source of personal income into four categories.

Specifications

Value	Condition(s)	Description	Notes
9	(INC_7 = DK, R, NS)	Required question was not answered (don’t know, refusal, not stated)	NS
1	(INC_7 = 1, 2)	Wages/salaries or self-employment	
2	(INC_7 = 4, 5, 10)	Employment insurance or worker’s compensation or social assistance/welfare	
3	(INC_7 = 6, 7, 8, 9)	Benefits from Canada or Quebec Pension Plan or job related retirement pensions, superannuation and annuities or RRSP/RRIF of Old Age Security and Guaranteed Income Supplement	
4	(INC_7 = 3, 11, 12, 13, 14, 15)	Dividends/interest or child tax benefit or child support or alimony or other or no income	

3) Total Household Income - All Sources - Grouped

Variable name: INCGHH

Based on: INCDHH

Description: This variable groups the total household income from all sources.

Note: Derived variable speficications were updated in 2009 due to changes in INCDHH answer categories.

Specifications

Value	Condition(s)	Description	Notes
9	(INCDHH = DK, R, NS)	Required question was not answered (don’t know, refusal, not stated)	NS
1	INCDHH = (1, 2, 3, 4, or 5)	No income or less than $20,000	
2	INCDHH = 6 or 7	$20,000 to $39,999	
3	INCDHH = 8 or 9	$40,000 to $59,999	
4	INCDHH = 10 or 11	$60,000 to $79,999	
5	INCDHH = (12, 13, or 14)	$80,000 or more	

4) Personal Income - All Sources - Grouped

Variable name: INCGPER

Based on: INCDPER

Description: This variable indicates the respondent's personal income from all sources.

Specifications

Value	Condition(s)	Description	Notes
99	(INCDPER = = DK, R, NS)	Required question was not answered (don't know, refusal, not stated)	NS
96	INCDPER = 96	Population exclusions	NA
1	INCDPER = 1	No income	
2	INCDPER = (2, 3, 4, or 5)	Less than $20,000	
3	INCDPER = 6 or 7	$20,000 to $39,999	
4	INCDPER = 8 or 9	$40,000 to $59,999	
5	INCDPER = 10 or 11	$60,000 to $79,999	
6	INCDPER = (12, 13, or 14)	$80,000 or more	

5) Distribution of household income - National level

Variable name: INCDRCA

Based on: INCDADR

Description: This derived variable is a distribution of respondents in deciles (ten categories including approximately the same percentage of residents for each province) based on their value for INCDADR, ie. the adjusted ratio of their total household income to the low income cut-off corresponding to their household and community size. It provides, for each respondent, a relative measure of their household income to the household incomes of all other respondents.

Note: Deciles are generated using weighted data. Adjusted ratios are presented in increasing order, from smallest to largest, for all 10 provinces irrespective of household and community size. Derived variables are calculated only for valid responses (not stated, refusal and don't know are excluded). Boundaries are determined in order to derive deciles from the total weighted number of cases for which derived variables are calculated. The Territories are excluded from this derived variable.

Specifications

Value	Condition(s)	Description	Notes
96	Residents of Territories excluded	N/A	NA
99	INCDADR = 9.999999999	Not stated	NS
1	First 10% of respondents from the ascending list of adjusted ratios (INCDADR)	Decile 1	
2	Second 10% of respondents from the ascending list of adjusted ratios (INCDADR)	Decile 2	
3	Third 10% of respondents from the ascending list of adjusted ratios (INCDADR)	Decile 3	
4	Fourth 10% of respondents from the ascending list of adjusted ratios (INCDADR)	Decile 4	
5	Fifth 10% of respondents from the ascending list of adjusted ratios (INCDADR)	Decile 5	

6	Sixth 10% of respondents from the ascending list of adjusted ratios (INCDADR)	Decile 6	
7	Seventh 10% of respondents from the ascending list of adjusted ratios (INCDADR)	Decile 7	
8	Eighth 10% of respondents from the ascending list of adjusted ratios (INCDADR)	Decile 8	
9	Ninth 10% of respondents from the ascending list of adjusted ratios (INCDADR)	Decile 9	
10	Tenth 10% of respondents from the ascending list of adjusted ratios (INCDADR)	Decile 10	

6) Distribution of household income - Provincial level

Variable name: INCDRPR

Based on: INCDADR, GEO_PRV

Description: This derived variable is a distribution of residents of each province in deciles (ten categories including approximately the same percentage of residents for each province) based on their value for INCDADR, ie. the adjusted ratio of their total household income to the low income cut-off corresponding to their household and community size. It provides, for each respondent, a relative measure of their household income to the household incomes of all other respondents in the same province. The Territories are excluded from this derived variable.

Note: Deciles are generated using weighted data. Adjusted ratios are presented in increasing order, from smallest to largest, for each of the 10 provinces irrespective of household and community size. Derived variables are calculated only for valid responses (not stated, refusal, etc. are excluded). Boundaries are determined in order to derive deciles from the total weighted number of cases for which derived variables are calculated.

The INCDRPR values are based on a distribution of adjusted ratios for the residents of each of the 10 provinces. This variable should therefore be used in conjunction with the variable for the province of residence (GEO_PRV).

Specifications

Value	Condition(s)	Description	Notes
96	Residents of territories excluded	N/A	NA
99	INCDADR = 9.999999999	Not stated	NS
1	First 10% of respondents from the ascending list of adjusted ratios (INCDADR)	Decile 1	
2	Second 10% of respondents from the ascending list of adjusted ratios (INCDADR)	Decile 2	
3	Third 10% of respondents from the ascending list of adjusted ratios (INCDADR)	Decile 3	
4	Fourth 10% of respondents from the ascending list of adjusted ratios (INCDADR)	Decile 4	
5	Fifth 10% of respondents from the ascending list of adjusted ratios (INCDADR)	Decile 5	
6	Sixth 10% of respondents from the ascending list of adjusted ratios (INCDADR)	Decile 6	
7	Seventh 10% of respondents from the ascending list of adjusted ratios (INCDADR)	Decile 7	
8	Eighth 10% of respondents from the ascending list of adjusted ratios (INCDADR)	Decile 8	
9	Ninth 10% of respondents from the ascending list of adjusted ratios (INCDADR)	Decile 9	

10	Tenth 10% of respondents from the ascending list of adjusted ratios (INCDADR)	Decile 10	

7) Distribution of household income - Health region level

Variable name: INCDRRS

Based on: INCDADR, GEO_DHR4

Description: This derived variable is a distribution of residents of each health region in deciles (ten categories including approximately the same percentage of residents for each province) based on their value for INCDADR, ie. the adjusted ratio of their total household income to the low income cut-off corresponding to their household and community size. It provides, for each respondent, a relative measure of their household income to the household incomes of all other respondents in the same health region. The Territories are excluded from this derived variable.

Note: Deciles are generated using weighted data. Adjusted ratios are presented in increasing order, from smallest to largest, for each of the 117 health regions irrespective of household and community size. Derived variables are calculated only for valid responses (not stated, refusal, etc. are excluded). Boundaries are determined in order to derive deciles from the total weighted number of cases for which derived variables are calculated.

The INCDRRS values are based on a distribution of adjusted ratios for the residents of each of the 122 health regions. This variable should therefore be used in conjunction with the variable for the health region province of residence (GEO_DHR4).

Specifications

Value	Condition(s)	Description	Notes
96	Residents of Territories excluded	N/A	NA
99	INCDADR = 9.999999999	Not stated	NS
1	First 10% of respondents from the ascending list of adjusted ratios (INCDADR)	Decile 1	
2	Second 10% of respondents from the ascending list of adjusted ratios (INCDADR)	Decile 2	
3	Third 10% of respondents from the ascending list of adjusted ratios (INCDADR)	Decile 3	
4	Fourth 10% of respondents from the ascending list of adjusted ratios (INCDADR)	Decile 4	
5	Fifth 10% of respondents from the ascending list of adjusted ratios (INCDADR)	Decile 5	
6	Sixth 10% of respondents from the ascending list of adjusted ratios (INCDADR)	Decile 6	
7	Seventh 10% of respondents from the ascending list of adjusted ratios (INCDADR)	Decile 7	
8	Eighth 10% of respondents from the ascending list of adjusted ratios (INCDADR)	Decile 8	
9	Ninth 10% of respondents from the ascending list of adjusted ratios (INCDADR)	Decile 9	
10	Tenth 10% of respondents from the ascending list of adjusted ratios (INCDADR)	Decile 10	

Injuries (11 DVs)

Temporary Reformat

Value	Condition(s)	Description	Notes
**INWTSIC**			
LBSCSIC	INW_1 = 1 and<br>LBSCSIC not in (7,8,9)<br>else INWTSIC = INWCSIC		
**INWTSOC**			
	If INW_ = 1 then INWTSOC = LBSCSOC else INWTSOC = INWCSOC		Based on: INW_1, INWCSOC, LBSCSOC

1) Number of injuries in past 12 months

Variable name: INJG02

Based on: INJ_02

Description: The number of injuries in past 12 months. This is a regrouping of INJ_02.

Specifications

Value	Condition(s)	Description	Notes
1	INJ_02 = 1	Respondent had 1 injury in the past 12 months.	
2	INJ_02 = 2	Respondent had 2 injuries in the past 12 months.	
3	3=<INJ_02=<5	Respondent had 3 to 5 injuries in the past 12 months.	
4	INJ_02>=6	Respondent had 6 or more injuries in the past 12 months.	
6	96=<INJ_02=<98	Not stated	
9	INJ_02 = 99	Not applicable	

2) Most Serious Injury

Variable name: INJG05

Based on: INJ_05

Description: This variable groups the responses of most serious injury.

Specifications

Value	Condition(s)	Description	Notes
NA	INJ_05 = NA	Not applicable	
NS	INJ_05 = DK, R or NS	Respondent did not answer (don't know, refusal, not specified)	
1	INJ_05 = 1	Multiple injuries	
2	INJ_05 = 2	Broken/fractured bones	
3	INJ_05 = 3, 9	Burn/Scald/Chemical/ Poisoning	
4	INJ_05 = 4	Dislocation	
5	INJ_05 = 5	Sprain/strain	

6	INJ_05 = 6	Cut/puncture/bite	
7	INJ_05 = 7	Scrape/bruise/blister	
8	INJ_05 = 8, 10	Concussion/internal injury	
9	INJ_05 = 11	Other	

3) Most Serious Injury - body part affected - Grouped

Variable name: INJG06

Based on: INJ_06

Description: This variable groups the most serious injury by body part affected.

Specifications

Value	Condition(s)	Description	Notes
NA	INJ_06 = NA	Not applicable	
NS	INJ_06 = DK, R or NS	Respondent did not answer (don't know, refusal, not specified)	
1	INJ_06 = 1	Multiple sites	
2	INJ_06 = 2, 3, 4	Eyes/head/neck	
3	INJ_06 = 5	Shoulder/upper arm	
4	INJ_06 = 6	Elbow/lower arm	
5	(INJ_06 = 7, 8)	Wrist or hand	
6	(INJ_06 = 9, 10)	Hip/thigh	
7	INJ_06 = 11	Knee/lower leg	
8	INJ_06 = 12	Ankle/foot	
9	(INJ_06 = 13, 14)	Upper or lower back/upper or lower spine	
10	(INJ_06 = 15, 16)	Chest/abdomen/pelvis (excluding back and spine)	

4) Most Serious Injury - Place of occurrence - Grouped

Variable name: INJG08

Based on: INJ_08

Description: This variable groups the responses of most serious injury by place of occurrence.

Specifications

Value	Condition(s)	Description	Notes
96	INJ_01 = 2	Respondent did not suffer an injury	NA
99	(INJ_08 = DK, R, NS)	Required question was not answered (don’t know, refusal, not stated)	NS
1	INJ_08 = 1	In a home or its surrounding area	
2	(INJ_08 = 2, 3, 6)	Residential institution/school, college, university/other institution	

3	INJ_08 = 4	Sports or athletic area in school, college, university
4	INJ_08 = 5	Other sports or athletic area
5	INJ_08 = 7	Street, highway, sidewalk
6	INJ_08 = 8	Commercial area
7	(INJ_08 = 9, 10)	Industrial or construction area or farm
8	(INJ_08 = 11, 12)	Other (includes countryside, forest, lake, ocean, mountains, prairie, etc.)

5) Most Serious Injury - Activity when injured - Grouped

Variable name: INJG092

Based on: INJ_09

Description: This variable groups the responses of most serious injury by activity when injured.

Note: Due to new INJ_09 answer categories in 2009, groupings have been modified. INJG092 is similar to previous INJG09 but not identical.

Specifications

Value	Condition(s)	Description	Notes
96	INJ_01 = 2	Respondent did not suffer an injury	NA
99	INJ_09 in (97, 98, 99)	Required question was not answered (don't know, refusal, not stated)	NS
1	INJ_09 = 1	Sport or physical exercise	
2	INJ_09 = 2	Leisure or hobby	
3	INJ_09 = 3	Working at a job or business	
4	INJ_09 in (7,8)	Driver or passenger in/on road or off road motor vehicle	
5	INJ_09 = 4	Household chores, outdoor yard maintenance, home renovations or other unpaid work	
6	INJ_09 in (6,9)	Going up and down stairs or walking	
7	INJ_09 in (5,10)	Other, including sleeping, eating, personal care	

6) Most Serious Injury - How fell - Grouped

Variable name: INJG11A

Based on: INJ_11A

Description: This variable groups the responses of most serious injury by how the respondent fell.

Note: Similar to INJG11 produced previous to 2009.

Specifications

Value	Condition(s)	Description	Notes
6	INJ_01 = 2	Respondent did not suffer an injury	NA

6	INJ_10 = 2	Respondent did not suffer an injury as a result of a fall	NA
9	INJ_11A in (97, 98, 99)	Required question was not answered (don't know, refusal, not stated)	NS
1	INJ_11A = 1	While skating, skiing, or snowboarding	
6	INJ_11A = 2	While practicing other sports	
2	INJ_11A in (3, 6, 7)	Going up or down stairs/steps/from furniture/from elevated position	
3	INJ_11A = 4	Slip, trip, stumble or loss of balance on ice and snow	
4	INJ_11A = 5	Slip, trip, stumble or loss of balance on any other surface	
5	INJ_11A in (8, 9)	Due to health problems (e.g. faint, weakness, dizziness) / Other	

7) Most Serious Injury - Treated in clinic - Grouped

Variable name: INJG14C

Based on: INJ_14C, INJ_14L, INJ_14F

Description: This variable groups the responses according to whether the most serious injury was treated in a clinic.

Note: INJ_14 answer categories changed in 2009, therefore 'clinic' is not defined exactly as it was previous to 2009.

Specifications

Value	Condition(s)	Description	Notes
6	INJ_14C = 96	Respondent did not suffer an injury or did not receive medical attention within 48 hours.	NA
9	INJ_14C in (7, 8, 9) or<br>INJ_14L in (7, 8, 9) or<br>INJ_14F in (7, 8, 9)	At least one required question was not answered (don't know, refusal, not stated)	NS
1	INJ_14C = 1 or<br>INJ_14L = 1 or<br>INJ_14F = 1	Most serious injury treated in: a hospital outpatient clinic, community health centre or CLSC, or other clinic (walk-in clinic, appointment, sports)	
2	INJ_14C = 2 and<br>INJ_14L = 2 and<br>INJ_14F = 2	Most serious injury not treated in: a hospital outpatient clinic, community health centre or CLSC, or other clinic (walk-in clinic, appointment, sports)	

8) Most Serious Injury - treated at physio, mass therapist, chiro, other - Grouped

Variable name: INJG14J2

Based on: INJ_14M, INJ_14N, INJ_14K

Description: This variable groups the responses according to whether the most serious injury was treated at a physiotherapist or massage therapist's office, chiropractor's office, or in some other place

Note: This variable differs from INJG14J produced previous to 2009.

Specifications

Value	Condition(s)	Description	Notes
6	INJ_14M = 6	Respondent did not suffer an injury or did not	NA

		receive medical attention within 48 hours	
9	INJ_14M in (7, 8, 9) or<br>INJ_14N in (7, 8, 9) or<br>INJ_14K in (7, 8, 9)	At least one required question was not answered (don't know, refusal, not stated)	NS
1	INJ_14M = 1 or<br>INJ_14N = 1 or<br>INJ_14K = 1	Most serious injury treated at physiotherapist, massage therapist, chiropractor's office, or other place	
2	INJ_14M = 2 and<br>INJ_14N = 2 and<br>INJ_14K = 2	Most serious injury not treated at physiotherapist, massage therapist, chiropractor's office, or other place	

9) Other injuries - number (G)

Variable name: INJG17

Based on: INJ_17

Description: This variable groups the responses of number of other injuries.

Specifications

Value	Condition(s)	Description	Notes
6	INJ_17 = 96	Not Applicable	
7	INJ_17 = 97	Don't Know	
9	INJ_17 = 99	Not stated	
1	INJ_17 = 1	1 injury	
2	2=<INJ_17=<5	2 to 5 injuries	
3	6=<INJ_17	6 or more injuries	

10) Cause of Injury - Grouped

Variable name: INJGCAU

Based on: INJ_10, INJ_12

Description: This variable categorizes the respondent's cause of injury.

Note: Respondents who did not suffer any injuries in the past 12 months before the interview have been excluded from the population.

Specifications

Value	Condition(s)	Description	Notes
96	INJ_01= 2	Population exclusion	NA
99	(INJ_10 = 2, DK, R, NS) and<br>(INJ_12 = DK, R, NS)	At least one required question was not answered (don't know, refusal,<br>not stated)	NS
1	INJ_10 = 1	Fall (excluding transport)	
2	INJ_12 = 1	Transportation accident	
3	INJ_12 = 2	Accidentally bumped, pushed, bitten, etc. by person or animal	

4	INJ_12 = 3	Accidentally struck or crushed by object(s)
5	INJ_12 = 4	Accidental contact - sharp object, tool, machine
6	INJ_12 = 8	Overexertion or strenuous movement
7	INJ_12 = 5 or<br>INJ_12 = 6 or<br>INJ_12 = 7 or<br>INJ_12 = 9 or<br>INJ_12 = 10	Other, including:<br>- smoke, fire, flames<br>- accidental contact with hot object, liquid or gas<br>- extreme weather or natural disaster physical assault

11) Injury Status

Variable name: INJDSTT

Based on: INJ_01, INJ_16

Description: This variable indicates the injury status of the respondent.

Specifications

Value	Condition(s)	Description	Notes
6	DOINJ = 2	Module not selected	NA
9	(INJ_01=DK, R, NS) or<br>(INJ_16=DK, R, NS)	At least one required question was not answered (don't know, refusal, not stated)	NS
0	INJ_01=2 and<br>INJ_16=2	No injuries	
1	INJ_01=1 and<br>INJ_16=2	Activity-limiting injury only	
2	INJ_01=2 and<br>INJ_16=1	Treated (non-activity limiting) injury only	
3	INJ_01=1 and<br>INJ_16=1	Both activity-limiting and treated (non-activity limiting) injuries	

Workplace injury (1 DV)

Temporary Reformat

Value	Condition(s)	Description	Notes
**INWTSIC**			
LBSCSIC	INW_1 = 1	Job industry in which injury occurred. Occurred in current main job. Industry code taken from Labour Force Module (LBS).	
INWCSIC	INW_1 <> 1	Job industry in which injury occurred. Did not occur in current main job. Industry code derived from INW module.	
**INWTSOC**			
LBSCSOC	INW_1 = 1	Job occupation in which injury occurred. Occurred in current main job. Occupation code taken from Labour Force Module (LBS).	
INWCSOC	INW_1 <> 1	Job occupation in which injury occurred. Did not occur in current main job. Occupation code derived from INW module.	

1) Occupation group (SOC) where injury occurred - (G)

Variable name: INWGSOC

Based on: INWDOCG

Description: This variable groups the occupation classification of the respondent where the injury occurred.

Specifications

Value	Condition(s)	Description	Notes
6	INWDOCG = 96	Population exclusions	NA
9	INWDOCG in (95, 99)	Respondent refused, did not know, or did not state their occupation or their occupation was uncodable	NS
1	INWDOCG in (01, 03, 04, 05, 06)	Occupations relating to Management, Natural and Applied Sciences , Health, Social Sciences, Education, Religion, Art, Culture and Recreation	
2	INWDOCG = 02	Occupations relating to Business, Finance, Administration	
3	INWDOCG = 07	Occupations relating to Sales and Service	
4	INWDOCG = 08	Occupations relating to Trades, Transport and Equipment Operator	
5	INWDOCG in (09, 10)	Occupations Unique to Primary Industry, Processing, Manufacturing and Utilities	

Labour force (5 DVs)

1) Employment status - 12 months - (G)

Variable name: LBSG31

Based on: LBS_31

Description: This variable groups the employment status of the respondent.

Specifications

Value	Condition(s)	Description	Notes
6	LBS_31 = 6	Not Applicable	
9	LBS_31 = 9	Not Stated	
1	LBS_31 = 1	Employee	
2	2=<LBS_31=<3	Self-employed	

2) Total usual hours worked - current jobs - Grouped

Variable name: LBSGHPW

Based on: LBSDHPW

Description: This variable indicates the total number of hours the respondent worked per week.

Note: Respondents aged less than 15 or more than 75 years old or who did not work in the week prior to the interview have been excluded from the population.

Specifications

Value	Condition(s)	Description	Notes
996	LBSDHPW = 996	Population exclusions	NA
999	LBSDHPW = 999	At least one required question was not answered (don't know, refusal, not stated)	NS
LBSDHPW	LBSDHPW < 99	Number of hours worked per week	
99	LBSDHPW >= 99	99 hours or more	

3) Full-time/part-time working status (for total usual hours)

Variable name: LBSDPFT

Based on: LBSDHPW

Description: This variable indicates if the respondent works full-time or part-time.

Note: Respondents aged less than 15 or more than 75 years old or who did not work in the week prior to the interview have been excluded from the population.

Specifications

Value	Condition(s)	Description	Notes
6	LBSDHPW = NA	Population exclusion	NA

9	LBSDHPW = NS	At least one required question was not answered (don’t know, refusal, not stated)	NS
1	LBSDHPW >= 30	Full-time	
2	LBSDHPW < 30	Part-time	

4) Working status last week

Variable name: LBSDWSS

Based on: LBS_01, LBS_02

Description: This variable classifies the respondent based on his/her working status in the week prior to the interview.

Note: Respondents aged less than 15 or more than 75 years old have been excluded from the population.

Specifications

Value	Condition(s)	Description	Notes
6	DHH_AGE < 15 or<br>DHH_AGE > 75	Population exclusion	NA
1	LBS_01 = 1	Worked at a job or business	
2	LBS_02 = 1	Had a job but did not work (absent)	
3	LBS_02 = 2	Did not have a job	
4	LBS_01 = 3	Permanently unable to work	
9	(LBS_02 = DK, R, NS) or (LBS_01 = DK, R, NS)	At least one required question was not answered (don't know, refusal, not stated)	NS

5) Occupation group - (G)

Variable name: LBSGSOC

Based on: LBSDOCG

Description: This variable groups the occupation classification of the respondent.

Specifications

Value	Condition(s)	Description	Notes
6	LBSDOCG = 96	Respondent did not work at a job or business in the past year or age was out of range	
9	LBSDOCG = 95, 99	Respondent refused, did not know,<br>or did not state their occupation or their occupation was uncodable	
1	LBSDOCG = ‘01’, ‘03’, ‘04’, ‘05’, ‘06’	Occupations relating to Management, Natural and Applied Sciences , Health, Social Sciences, Education, Religion, Art, Culture and Recreation	
2	LBSDOCG = ‘02’	Occupations relating to Business, Finance, Administration	
3	LBSDOCG = ‘07’	Occupations relating to Sales and Service	
4	LBSDOCG = ‘08’	Occupations relating to Trades, Transport and Equipment Operator	

5	LBSDOCG = ‘09’, ‘10’	Occupations Unique to Primary Industry, Processing, Manufacturing and Utilities

Mastery (1 DV)

Temporary Reformat

Value	Condition(s)	Description	Notes
**MAST601**			
MAS_601	MAS_601 > 5	Carry through cases of RF, DK, NS	
(MAS_601 – 1)	MAS_601 <= 5	Rescale the answers for questions	
**MAST602**			
MAS_602	MAS_602 > 5	Carry through cases of RF, DK, NS	
(MAS_602 – 1)	MAS_602 <= 5	Rescale the answers for questions	
**MAST603**			
MAS_603	MAS_603 > 5	Carry through cases of RF, DK, NS	
(MAS_603 – 1)	MAS_603 <= 5	Rescale the answers for questions	
**MAST604**			
MAS_604	MAS_604 > 5	Carry through cases of RF, DK, NS	
(MAS_604 – 1)	MAS_604 <= 5	Rescale the answers for questions	
**MAST605**			
MAS_605	MAS_605 > 5	Carry through cases of RF, DK, NS	
(MAS_605 – 1)	MAS_605 <= 5	Rescale the answers for questions	
**MAST606**			
MAS_606	MAS_606 > 5	Carry through cases of RF, DK, NS	
(4 – MAST606)	MAST606 <= 4	Invert scale for rescaled questions	
(MAS_606 – 1)	MAS_606 <= 5	Rescale the answers for questions	
**MAST607**			
(4 – MAST607)	MAST607 <= 4	Invert scale for rescaled questions	
MAS_607	MAS_607 > 5	Carry through cases of RF, DK, NS	
(MAS_607 – 1)	MAS_607 <= 5	Rescale the answers for questions	

1) Derived Mastery Scale

Variable name: MASDM1

Based on: MAS_601, MAS_602, MAS_603, MAS_604, MAS_605, MAS_606, MAS_607

Description: This variable measures sense of mastery, that is, the extent to which individuals believe that their life-chances are under their control.

Note: Higher scores indicate superior mastery.

Internet site: www.jstor.org/

Specifications

Value	Condition(s)	Description	Notes
96	DOMAS = 2	Module not selected	NA
99	ADM_PRX = 1	Module not asked - proxy interview	NS

99	(MAST601 = DK, R, NS) or<br>(MAST602 = DK, R, NS) or<br>(MAST603 = DK, R, NS) or<br>(MAST604 = DK, R, NS) or<br>(MAST605 = DK, R, NS) or<br>(MAST606 = DK, R, NS) or<br>(MAST607 = DK, R, NS)	At least one required question was not answered (don’t know, refusal, not stated)	NS
MAST601 +<br>MAST602 +<br>MAST603 +<br>MAST604 +<br>MAST605 +<br>MAST606<br>+MAST607	(0 <= MAST601 <= 4) and<br>(0 <= MAST602 <= 4) and<br>(0 <= MAST603 <= 4) and<br>(0 <= MAST604 <= 4) and<br>(0 <= MAST605 <= 4) and<br>(0 <= MAST606 <= 4) and<br>(0 <= MAST607 <= 4)	Score obtained on the mastery scale	(min: 0; max: 28)

Reference: Pearlin, LI and Schooler, C, Journal of health and Social Behavior, "The Structure of Coping", 1981, vol.19, p.2-21.

Maternal experiences - Breastfeeding (3 DVs)

1) Length of exclusive breastfeeding

Variable name: MEXDEBF2

Based on: MEX_03, MEX_06, MEX_07

Description: This variable provides the length of time that the respondent exclusively breastfed her last baby.

Note: This variable is an update of MEXDEBF. It includes more categories, covers the 6 month period in a single category, and takes into account conflicting information provided in MEX_06 and MEX_07. Respondents who had not given birth in the past 5 years or who were less than 15 years old or more than 55 years old are excluded from the population. Since the variable is used to measure only the final duration of exclusive breastfeeding, mothers who are still breastfeeding and who had not yet added any other liquid or solid foods to the baby's feeds are also excluded.

Specifications

Value	Condition(s)	Description	Notes
96	DHH_SEX = 1 or<br>DHH_AGE < 15 or<br>DHH_AGE > 55 or<br>MEX_01 = 2 or<br>(MEX_05 = 1 AND<br>MEX_07 = 13)	Population exclusions	NA
99	ADM_PRX = 1	Module not asked - Proxy Interview	NS
99	(MEX_03 in (97, 98, 99)) or<br>(MEX_06 in (97, 98, 99)) or<br>(MEX_07 in (97, 98, 99))	At least one required question was not answered (don’t know, refusal, not stated)	NS
0	MEX_03 = 2	Has not breastfed her last baby	
1	(MEX_07 = 1 and<br>MEX_06 in (1,2,3,4,5,6,7,8,9,10,11,12)) or<br>(MEX_06 = 1 and<br>MEX_07 > MEX_06 and<br>MEX_07 < 13) or<br>(MEX_06 = 96 and<br>MEX_07 = 1) or<br>(MEX_07 = 13 and<br>MEX_06 = 1)	Less than 1 week	
2	(MEX_07 in (2,3) and<br>MEX_06 in (2,3,4,5,6,7,8,9,10,11,12)) or<br>(MEX_06 in (2,3) and<br>MEX_07 > MEX_06 and<br>MEX_07 < 13) or<br>(MEX_06 = 96 and<br>MEX_07 in (2,3)) or<br>(MEX_07 = 13 and<br>MEX_06 in (2,3))	1 week to less than 5 weeks	
3	(MEX_07 in (4,5) and<br>MEX_06 in (4,5,6,7,8,9,10,11,12)) or<br>(MEX_06 in (4,5) and<br>MEX_07 > MEX_06 and<br>MEX_07 < 13) or<br>(MEX_06 = 96 and<br>MEX_07 in (4,5)) or<br>(MEX_07 = 13 and<br>MEX_06 in (4,5))	5 weeks to less than 12 weeks	

4	(MEX_07 = 6 and<br>MEX_06 in (6,7,8,9,10,11,12)) or<br>(MEX_06 = 6 and<br>MEX_07 > MEX_06 and<br>MEX_07 < 13) or<br>(MEX_06 = 96 and<br>MEX_07 = 6) or<br>(MEX_07 = 13 and<br>MEX_06 = 6)	12 weeks to less than 16 weeks (3 months)	
5	(MEX_07 = 7 and<br>MEX_06 in (7,8,9,10,11,12)) or<br>(MEX_06 = 7 and<br>MEX_07 > MEX_06 and<br>MEX_07 < 13) or<br>(MEX_06 = 96 and<br>MEX_07 = 7) or<br>(MEX_07 = 13 and<br>MEX_06 = 7)	16 weeks to less than 20 weeks (4 months)	
6	(MEX_07 = 8 and<br>MEX_06 in (8,9,10,11,12)) or<br>(MEX_06 = 8 and<br>MEX_07 > MEX_06 and<br>MEX_07 < 13) or<br>(MEX_06 = 96 and<br>MEX_07 = 8) or<br>(MEX_07 = 13 and<br>MEX_06 = 8)	20 weeks to less than 24 weeks (5 months)	
7	(MEX_07 = 9 and<br>MEX_06 in (9,10,11,12)) or<br>(MEX_06 = 9 and<br>MEX_07 > MEX_06 and<br>MEX_07 < 13) or<br>(MEX_06 = 96 and<br>MEX_07 = 9) or<br>(MEX_07 = 13 and<br>MEX_06 = 9)	24 weeks to less than 28 weeks (6 months)	
8	(MEX_07 in (10,11,12) and<br>MEX_06 in (10,11,12)) or<br>(MEX_06 IN (10,11,12) and<br>MEX_07 > MEX_06 and<br>MEX_07 < 13) or<br>(MEX_06 = 96 and<br>MEX_07 in (10,11,12)) or<br>(MEX_07 = 13 and<br>MEX_06 in (10,11,12))	7 months or more	

2) Exclusively Breastfed for 6 months (or more)

Variable name: MEXFEB6

Based on: MEX_03, MEX_06, MEX_07

Description: This variable indicates whether the respondent exclusively breastfed her last baby for at least 6 months.

Note: Health Canada recommends exclusive breastfeeding for a period of up to 6 months. This variable indicates the number of mothers who followed this recommendation. Respondents who had not given birth in the past 5 years or who were less than 15 years old or more than 55 years old are excluded from the population. Since the variable is used to measure only the final duration of exclusive breastfeeding, mothers who still breastfed and who had not yet added any other liquid or solid foods to the baby's feeds are also excluded.

Specifications

Value	Condition(s)	Description	Notes
6	DHH_SEX = 1 or	Population exclusions	NA

	DHH_AGE < 15 or<br>DHH_AGE > 55 or<br>MEX_01 = 2 or<br>(MEX_05 = 1 and<br>MEX_07 = 13)		
9	ADM_PRX = 1	Module not asked - proxy interview	NS
9	(MEX_03 in (7,8,9)) or<br>(MEX_06 in (7,8,9)) or<br>(MEX_07 in (7,8,9))	At least one required question was not answered (don't know, refusal, not stated)	NS
1	(mex_07 in (9:12) and<br>mex_06 in (9:12)) or<br>(mex_06 = 96 and<br>mex_07 in (9:12)) or<br>(mex_07 = 13 and<br>mex_06 in (9:12))	Had exclusively breastfed her last baby for at least 6 months	
2	(mex_03=2) or<br>(mex_06 <9) or<br>(mex_07 <9)	Had not exclusively breastfed her last baby for at least 6 months	

Maternal experiences - Smoking during pregnancy (4 DVs)

1) No. of cigarettes daily - last pregnancy (daily smoker)

Variable name: MXSG02

Based on: MXS_02

Description: This variable groups the number of cigarettes the respondent smoked during her last pregnancy.

Specifications

Value	Condition(s)	Description	Notes
96	MXS_02 = 96	Not applicable	
99	MXS_02 = 97, 98, 99	Not stated	
1	MXS_02 = 1	Respondent smoked one cigarette daily during her last pregnancy.	
2	MXS_02 = 2	Respondent smoked 2 cigarettes daily during her last pregnancy.	
3	MXS_02 = 3	Respondent smoked 3 cigarettes daily during her last pregnancy.	
4	MXS_02 = 4	Respondent smoked 4 cigarettes daily during her last pregnancy.	
5	MXS_02 = 5	Respondent smoked 5 cigarettes daily during her last pregnancy.	
6	6=<MXS_02 =<10	Respondent smoked 6 to 10 cigarettes daily during her last pregnancy.	
7	11=<MXS_02 =<15	Respondent smoked 11 to 15 cigarettes daily during her last pregnancy.	
8	16=<MXS_02	Respondent smoked 16 or more cigarettes daily during her last pregnancy.	

2) No. of cigarettes daily - last pregnancy (occasional smoker)

Variable name: MXSG03

Based on: MXS_03

Description: This variable groups the number of cigarettes the respondent smoked during her last pregnancy.

Specifications

Value	Condition(s)	Description	Notes
96	MXS_03 = 96	Not applicable	
99	MXS_03 = 97, 98, 99	Not stated	
1	MXS_03 = 1	Respondent smoked one cigarette daily during her last pregnancy.	
2	MXS_03 = 2	Respondent smoked 2 cigarettes daily during her last pregnancy.	
3	MXS_03 = 3	Respondent smoked 3 cigarettes daily during her last pregnancy.	

4	MXS_03 = 4	Respondent smoked 4 cigarettes daily during her last pregnancy.	
5	MXS_03 = 5	Respondent smoked 5 cigarettes daily during her last pregnancy.	
6	6=<MXS_03	Respondent smoked at least 6 cigarettes daily during her last pregnancy.	

3) No. of cigarettes daily - while breastfeeding (daily smoker)

Variable name: MXSG05

Based on: MXS_05

Description: This variable groups the number of cigarettes the respondent smoked while while breastfeeding her last baby.

Specifications

Value	Condition(s)	Description	Notes
96	MXS_05 = 96	Not applicable	
99	MXS_05 = 97, 98, 99	Not stated	
1	MXS_05 = 1	Respondent smoked one cigarette daily while breastfeeding her last baby.	
2	MXS_05 = 2	Respondent smoked 2 cigarettes daily while breastfeeding her last baby.	
3	MXS_05 = 3	Respondent smoked 3 cigarettes daily while breastfeeding her last baby.	
4	MXS_05 = 4	Respondent smoked 4 cigarettes daily while breastfeeding her last baby.	
5	MXS_05 = 5	Respondent smoked 5 cigarettes daily while breastfeeding her last baby.	
6	6=<MXS_05=<10	Respondent smoked 6 to 10 cigarettes daily while breastfeeding her last baby.	
7	11=<MXS_05=<15	Respondent smoked 11 to 15 cigarettes daily while breastfeeding her last baby.	
8	16=<MXS_05	Respondent smoked at least 16 cigarettes daily while breastfeeding her last baby.	

4) No. of cigarettes daily - while breastfeeding (occasional smoker)

Variable name: MXSG06

Based on: MXS_06

Description: This variable groups the number of cigarettes the respondent smoked while while breastfeeding her last baby.

Specifications

Value	Condition(s)	Description	Notes
96	MXS_06 = 96	Not applicable	
99	MXS_06 = 97, 98, 99	Not stated	

1	MXS_06 = 1	Respondent smoked one cigarette daily while breastfeeding her last baby.
2	MXS_06 = 2	Respondent smoked 2 cigarettes daily while breastfeeding her last baby.
3	MXS_06 = 3	Respondent smoked 3 cigarettes daily while breastfeeding her last baby.
4	MXS_06 = 4	Respondent smoked 4 cigarettes daily while breastfeeding her last baby.
5	MXS_06 = 5	Respondent smoked 5 cigarettes daily while breastfeeding her last baby.
6	6=<MXS_06	Respondent smoked at least 6 cigarettes daily while breastfeeding her last baby.

Oral health 2 (2 DVs)

1) Social Limitation Due to Oral Health Status

Variable name: OH2FLIM

Based on: OH2_23, OH2_24

Description: This variable indicates whether the respondent's oral health status impacts on social functioning as measured by avoiding conversation or contact with others, or by avoiding laughing or smiling.

Specifications

Value	Condition(s)	Description	Notes
6	DOOH2 = 2	Module not selected	NA
9	ADM_PRX = 1	Module not asked - proxy interview	NS
2	(OH2_23 = 3, 4) and<br>(OH2_24 = 3, 4)	No social limitation due to oral condition	
1	(OH2_23 = 1, 2) or<br>(OH2_24 = 1, 2)	Social limitation experienced due to oral condition	
9	(OH2_23 = DK, R, NS) or<br>(OH2_24 = DK, R, NS)	At least one required question was not answered (don't know, refusal, not stated)	NS

2) Oral and Facial Pain and Discomfort

Variable name: OH2FOFP

Based on: OH2_25A, OH2_25B, OH2_25C, OH2_25D, OH2_25E, OH2_25F, OH2_25G

Description: This variable indicates the presence of oral and facial pain in the past month.

Specifications

Value	Condition(s)	Description	Notes
6	DOOH2 = 2	Module not selected	NA
9	ADM_PRX = 1	Module not asked - proxy interview	NS
2	OH2_25A = 2 and<br>OH2_25B = 2 and<br>OH2_25C = 2 and<br>OH2_25D = 2 and<br>OH2_25E = 2 and<br>OH2_25F = 2 and<br>OH2_25G = 2	Has not experienced any oral or facial pain or discomfort in the past month	
1	OH2_25A = 1 or<br>OH2_25B = 1 or<br>OH2_25C = 1 or<br>OH2_25D = 1 or<br>OH2_25E = 1 or<br>OH2_25F = 1 or<br>OH2_25G = 1	Has experienced some oral or facial pain or discomfort in the past month	

9	(OH2_25A = DK, R, NS) or<br>(OH2_25B = DK, R, NS) or<br>(OH2_25C = DK, R, NS) or<br>(OH2_25D = DK, R, NS) or<br>(OH2_25E = DK, R, NS) or<br>(OH2_25F = DK, R, NS) or<br>(OH2_25G = DK, R, NS)	At least one required question was not answered (don’t know, refusal, not stated)	NS

Physical activities (10 DVs)

1) Number of times / 3 months - ice hockey - (G)

Variable name: PACG2G

Based on: PAC_2G

Description: This variable indicates the number of times the respondent praticed ice hockey in the last 3 months.

Note: PAC module questions refer to leisure time activities.

Specifications

Value	Condition(s)	Description	Notes
996	PAC_2G = 996	Population exclusions	NA
999	PAC_2G in (997, 998, 999)	At least one required question was not answered (don't know, refusal, not stated)	NS
50	dhh_age >= 25 and PAC_3G = 4 and PAC_2G > 50	Maximum value of number of times / 3 months - ice hockey for respondents aged 25 or older who played for more than one hour.	
PAC_2G	PAC_2G	Number of times / 3 months - ice hockey	

2) Daily Energy Expenditure in Leisure Time Physical Activities

Variable name: PACDEE

Based on: PAC_1V, PAC_2A, PAC_2B, PAC_2C, PAC_2D, PAC_2E, PAC_2F, PAC_2G, PAC_2H, PAC_2I, PAC_2J, PAC_2K, PAC_2L, PAC_2M, PAC_2N, PAC_2O, PAC_2P, PAC_2Q, PAC_2R, PAC_2S, PAC_2T, PAC_2U, PAC_2W, PAC_2X, PAC_2Z, PAC_3A, PAC_3B, PAC_3C, PAC_3D, PAC_3E, PAC_3F, PAC_3G, PAC_3H, PAC_3I, PAC_3J, PAC_3K, PAC_3L, PAC_3M, PAC_3N, PAC_3O, PAC_3P, PAC_3Q, PAC_3R, PAC_3S, PAC_3T, PAC_3U, PAC_3W, PAC_3X, PAC_3Z

Description: This variable is a measure of the average daily energy expended during leisure time activities by the respondent in the past three months.

Note: Energy Expenditure (EE) is calculated using the frequency and duration per session of the physical activity as well as the MET value of the activity. The MET is a value of metabolic energy cost expressed as a multiple of the resting metabolic rate. For example, an activity of 4 METS requires four times the amount of energy as compared to when the body is at rest.

EE (Energy Expenditure for each activity) = (N X D X METvalue) / 365
Where:
N = the number of times a respondent engaged in an activity over a 12 month period
D = the average duration in hours of the activity
MET value = the energy cost of the activity expressed as kilocalories expended per kilogram of body weight per hour of activity (kcal/kg per hour)/365 (to convert yearly data into daily data)

MET values tend to be expressed in three intensity levels (i.e. low, medium, high). The CCHS questions did not ask the respondent to specify the intensity level of their activities. Therefore the MET values adopted correspond to the low intensity value of each activity. This approach is adopted from the Canadian Fitness and Lifestyle Research Institute because individuals tend to overestimate the intensity, frequency and duration of their activities.

Variable Name	Activity	MET Value (kcal/kg/hr)
PACDEEA	WALKING FOR EXERCISE	3
PACDEEB	GARDENING OR YARD WORK	3
PACDEEC	SWIMMING	3
PACDEED	BICYCLING	4
PACDEEE	POPULAR OR SOCIAL DANCE	3

PACDEEF	HOME EXERCISES	3
PACDEEG	ICE HOCKEY	6
PACDEEH	ICE SKATING	4
PACDEEI	IN-LINE SKATING OR ROLLERBLADING	5
PACDEEJ	JOGGING OR RUNNING*	9.5
PACDEEK	GOLFING	4
PACDEEL	EXERCISE CLASS OR AEROBICS	4
PACDEEM	DOWNHILL SKIING OR SNOWBOARDING	4
PACDEEN	BOWLING	2
PACDEEO	BASEBALL OR SOFTBALL	3
PACDEEP	TENNIS	4
PACDEEQ	WEIGHT-TRAINING	3
PACDEER	FISHING	3
PACDEES	VOLLEYBALL	5
PACDEET	BASKETBALL	6
PACDEEZ	SOCCER	5
PACDEEU	OTHER (U)*	4
PACDEEW	OTHER (W)*	4
PACDEEX	OTHER (X)*	4

* Jogging (MET value 7) and running (MET value 12) fall under one category. Therefore, the MET value for the combined activity is the average of their MET values (9.5). Since it is difficult to assign a MET value to the category "Other Activities", the MET value used is the average of the listed activities except for the average value of jogging and running. Here, the average value of jogging and running is replaced by the value for jogging only. Some activities have MET values lower than the average, however, this approach is consistent with other studies, such as the Campbell's Survey and the Ontario Health Survey (OHS).

* Times were assigned an average duration value for the calculation, as with NPHS:
(13 minutes or .2167 hour, 23 minutes or .3833 hour, 45 minutes or .75 hour, 60 minutes or 1 hour)

Beginning in CCHS cycle 2.1, the list of activities (PAC_1n) changed slightly from previous CCHS cycles: The activity "Soccer" was asked explicitly in Cycle 2.1. For Cycle 1.1, this activity was part of the "Other" activities.

Temporary Reformat

Value	Condition(s)	Description	Notes
**PACDEEA**			
0	PAC_3A = NA	Did not participate in activity	WALKING FOR EXERCISE
0	(PAC_3A = DK, R, NS)	Required question was not answered (don't know, refusal, not stated)	WALKING FOR EXERCISE
(PAC_2A × 4 × .2167 × 3) / 365	PAC_3A = 1	Calculate EE for < 15 min*	WALKING FOR EXERCISE
(PAC_2A × 4 × .3833 × 3) / 365	PAC_3A = 2	Calculate EE for 16 to 30 min*	WALKING FOR EXERCISE
(PAC_2A × 4 × .75 × 3) / 365	PAC_3A = 3	Calculate EE for 31 to 60 min*	WALKING FOR EXERCISE
(PAC_2A × 4 × 1 × 3) / 365	PAC_3A = 4	Calculate EE for > 60 min*	WALKING FOR EXERCISE
**PACDEEB**			
0	PAC_3B = NA	Did not participate in activity	GARDENING OR YARD WORK
0	(PAC_3B = DK, R, NS)	Required question was not answered (don't know, refusal, not stated)	GARDENING OR YARD WORK
(PAC_2B × 4 × .2167 × 3) / 365	PAC_3B = 1	Calculate EE for < 15 min*	GARDENING OR YARD WORK
(PAC_2B × 4 × .3833 × 3) / 365	PAC_3B = 2	Calculate EE for 16 to 30 min*	GARDENING OR YARD WORK
(PAC_2B × 4 × .75 × 3) / 365	PAC_3B = 3	Calculate EE for 31 to 60 min*	GARDENING OR YARD WORK
(PAC_2B × 4 × 1 × 3) / 365	PAC_3B = 4	Calculate EE for > 60 min*	GARDENING OR YARD WORK

**PACDEEC**			
0	PAC_3C = NA	Did not participate in activity	SWIMMING
0	(PAC_3C = DK, R, NS)	Required question was not answered (don't know, refusal, not stated)	SWIMMING
(PAC_2C × 4 × .2167 × 3) / 365	PAC_3C = 1	Calculate EE for < 15 min*	SWIMMING
(PAC_2C × 4 × .3833 × 3) / 365	PAC_3C = 2	Calculate EE for 16 to 30 min*	SWIMMING
(PAC_2C × 4 × .75 × 3) / 365	PAC_3C = 3	Calculate EE for 31 to 60 min*	SWIMMING
(PAC_2C × 4 × 1 × 3) / 365	PAC_3C = 4	Calculate EE for > 60 min*	SWIMMING
**PACDEED**			
0	PAC_3D = NA	Did not participate in activity	BICYCLING
0	(PAC_3D = DK, R, NS)	Required question was not answered (don't know, refusal, not stated)	BICYCLING
(PAC_2D × 4 × .2167 × 4) / 365	PAC_3D = 1	Calculate EE for < 15 min*	BICYCLING
(PAC_2D × 4 × .3833 × 4) / 365	PAC_3D = 2	Calculate EE for 16 to 30 min*	BICYCLING
(PAC_2D × 4 × .75 × 4) / 365	PAC_3D = 3	Calculate EE for 31 to 60 min*	BICYCLING
(PAC_2D × 4 × 1 × 4) / 365	PAC_3D = 4	Calculate EE for > 60 min*	BICYCLING
**PACDEEE**			
0	PAC_3E = NA	Did not participate in activity	POPULAR OR SOCIAL DANCE
0	(PAC_3E = DK, R, NS)	Required question was not answered (don't know, refusal, not stated)	POPULAR OR SOCIAL DANCE
(PAC_2E × 4 × .2167 × 3) / 365	PAC_3E = 1	Calculate EE for < 15 min*	POPULAR OR SOCIAL DANCE
(PAC_2E × 4 × .3833 × 3) / 365	PAC_3E = 2	Calculate EE for 16 to 30 min*	POPULAR OR SOCIAL DANCE
(PAC_2E × 4 × .75 × 3) / 365	PAC_3E = 3	Calculate EE for 31 to 60 min*	POPULAR OR SOCIAL DANCE
(PAC_2E × 4 × 1 × 3) / 365	PAC_3E = 4	Calculate EE for > 60 min*	POPULAR OR SOCIAL DANCE
**PACDEEF**			
0	PAC_3F = NA	Did not participate in activity	HOME EXERCISES
0	(PAC_3F = DK, R, NS)	Required question was not answered (don't know, refusal, not stated)	HOME EXERCISES
(PAC_2F × 4 × .2167 × 3) / 365	PAC_3F = 1	Calculate EE for < 15 min*	HOME EXERCISES
(PAC_2F × 4 × .3833 × 3) / 365	PAC_3F = 2	Calculate EE for 16 to 30 min*	HOME EXERCISES
(PAC_2F × 4 × .75 × 3) / 365	PAC_3F = 3	Calculate EE for 31 to 60 min*	HOME EXERCISES
(PAC_2F × 4 × 1 × 3) / 365	PAC_3F = 4	Calculate EE for > 60 min*	HOME EXERCISES
**PACDEEG**			

0	PAC_3G = NA	Did not participate in activity	ICE HOCKEY
0	(PAC_3G = DK, R, NS)	Required question was not answered (don't know, refusal, not stated)	ICE HOCKEY
(PAC_2G × 4 × .2167 × 6) / 365	PAC_3G = 1	Calculate EE for < 15 min*	ICE HOCKEY
(PAC_2G × 4 × .3833 × 6) / 365	PAC_3G = 2	Calculate EE for 16 to 30 min*	ICE HOCKEY
(PAC_2G × 4 × .75 × 6) / 365	PAC_3G = 3	Calculate EE for 31 to 60 min*	ICE HOCKEY
(PAC_2G × 4 × 1 × 6) / 365	PAC_3G = 4	Calculate EE for > 60 min*	ICE HOCKEY
**PACDEEH**			
0	PAC_3H = NA	Did not participate in activity	ICE SKATING
0	(PAC_3H = DK, R, NS)	Required question was not answered (don't know, refusal, not stated)	ICE SKATING
(PAC_2H × 4 × .2167 × 4) / 365	PAC_3H = 1	Calculate EE for < 15 min*	ICE SKATING
(PAC_2H × 4 × .3833 × 4) / 365	PAC_3H = 2	Calculate EE for 16 to 30 min*	ICE SKATING
(PAC_2H × 4 × .75 × 4) / 365	PAC_3H = 3	Calculate EE for 31 to 60 min*	ICE SKATING
(PAC_2H × 4 × 1 × 4) / 365	PAC_3H = 4	Calculate EE for > 60 min*	ICE SKATING
**PACDEEI**			
0	PAC_3I = NA	Did not participate in activity	IN-LINE SKATING OR ROLLERBLADING
0	(PAC_3I = DK, R, NS)	Required question was not answered (don't know, refusal, not stated)	IN-LINE SKATING OR ROLLERBLADING
(PAC_2I × 4 × .2167 × 5) / 365	PAC_3I = 1	Calculate EE for < 15 min*	IN-LINE SKATING OR ROLLERBLADING
(PAC_2I × 4 × .3833 × 5) / 365	PAC_3I = 2	Calculate EE for 16 to 30 min*	IN-LINE SKATING OR ROLLERBLADING
(PAC_2I × 4 × .75 × 5) / 365	PAC_3I = 3	Calculate EE for 31 to 60 min*	IN-LINE SKATING OR ROLLERBLADING
(PAC_2I × 4 × 1 × 5) / 365	PAC_3I = 4	Calculate EE for > 60 min*	IN-LINE SKATING OR ROLLERBLADING
**PACDEEJ**			
0	PAC_3J = NA	Did not participate in activity	JOGGING OR RUNNING
0	(PAC_3J = DK, R, NS)	Required question was not answered (don't know, refusal, not stated)	JOGGING OR RUNNING
(PAC_2J × 4 × .2167 × 9.5) / 365	PAC_3J = 1	Calculate EE for < 15 min*	JOGGING OR RUNNING
(PAC_2J × 4 × .3833 × 9.5) / 365	PAC_3J = 2	Calculate EE for 16 to 30 min*	JOGGING OR RUNNING
(PAC_2J × 4 × .75 × 9.5) / 365	PAC_3J = 3	Calculate EE for 31 to 60 min*	JOGGING OR RUNNING

(PAC_2J × 4 × 1 × 9.5) / 365	PAC_3J = 4	Calculate EE for > 60 min*	JOGGING OR RUNNING
**PACDEEK**			
0	PAC_3K = NA	Did not participate in activity	GOLFING
0	(PAC_3K = DK, R, NS)	Required question was not answered (don't know, refusal, not stated)	GOLFING
(PAC_2K × 4 × .2167 × 4) / 365	PAC_3K = 1	Calculate EE for < 15 min*	GOLFING
(PAC_2K × 4 × .3833 × 4) / 365	PAC_3K = 2	Calculate EE for 16 to 30 min*	GOLFING
(PAC_2K × 4 × .75 × 4) / 365	PAC_3K = 3	Calculate EE for 31 to 60 min*	GOLFING
(PAC_2K × 4 × 1 × 4) / 365	PAC_3K = 4	Calculate EE for > 60 min*	GOLFING
**PACDEEL**			
0	PAC_3L = NA	Did not participate in activity	EXERCISE CLASS OR AEROBICS
0	(PAC_3L = DK, R, NS)	Required question was not answered (don't know, refusal, not stated)	EXERCISE CLASS OR AEROBICS
(PAC_2L × 4 × .2167 × 4) / 365	PAC_3L = 1	Calculate EE for < 15 min*	EXERCISE CLASS OR AEROBICS
(PAC_2L × 4 × .3833 × 4) / 365	PAC_3L = 2	Calculate EE for 16 to 30 min*	EXERCISE CLASS OR AEROBICS
(PAC_2L × 4 × .75 × 4) / 365	PAC_3L = 3	Calculate EE for 31 to 60 min*	EXERCISE CLASS OR AEROBICS
(PAC_2L × 4 × 1 × 4) / 365	PAC_3L = 4	Calculate EE for > 60 min*	EXERCISE CLASS OR AEROBICS
**PACDEEM**			
0	PAC_3M = NA	Did not participate in activity	DOWNHILL SKIING OR SNOWBOARDING
0	(PAC_3M = DK, R, NS)	Required question was not answered (don't know, refusal, not stated)	DOWNHILL SKIING OR SNOWBOARDING
(PAC_2M × 4 × .2167 × 4) / 365	PAC_3M = 1	Calculate EE for < 15 min*	DOWNHILL SKIING OR SNOWBOARDING
(PAC_2M × 4 × .3833 × 4) / 365	PAC_3M = 2	Calculate EE for 16 to 30 min*	DOWNHILL SKIING OR SNOWBOARDING
(PAC_2M × 4 × .75 × 4) / 365	PAC_3M = 3	Calculate EE for 31 to 60 min*	DOWNHILL SKIING OR SNOWBOARDING
(PAC_2M × 4 × 1 × 4) / 365	PAC_3M = 4	Calculate EE for > 60 min*	DOWNHILL SKIING OR SNOWBOARDING
**PACDEEN**			
0	PAC_3N = NA	Did not participate in activity	BOWLING
0	(PAC_3N = DK, R, NS)	Required question was not answered (don't know, refusal, not stated)	BOWLING
(PAC_2N × 4 × .2167 × 2) / 365	PAC_3N = 1	Calculate EE for < 15 min*	BOWLING

(PAC_2N × 4 × .3833 × 2) / 365	PAC_3N = 2	Calculate EE for 16 to 30 min*	BOWLING
(PAC_2N × 4 × .75 × 2) / 365	PAC_3N = 3	Calculate EE for 31 to 60 min*	BOWLING
(PAC_2N × 4 × 1 × 2) / 365	PAC_3N = 4	Calculate EE for > 60 min*	BOWLING
**PACDEEO**			
0	PAC_3O = NA	Did not participate in activity	BASEBALL OR SOFTBALL
0	(PAC_3O = DK, R, NS)	Required question was not answered (don't know, refusal, not stated)	BASEBALL OR SOFTBALL
(PAC_2O × 4 × .2167 × 3) / 365	PAC_3O = 1	Calculate EE for < 15 min*	BASEBALL OR SOFTBALL
(PAC_2O × 4 × .3833 × 3) / 365	PAC_3O = 2	Calculate EE for 16 to 30 min*	BASEBALL OR SOFTBALL
(PAC_2O × 4 × .75 × 3) / 365	PAC_3O = 3	Calculate EE for 31 to 60 min*	BASEBALL OR SOFTBALL
(PAC_2O × 4 × 1 × 3) / 365	PAC_3O = 4	Calculate EE for > 60 min*	BASEBALL OR SOFTBALL
**PACDEEP**			
0	PAC_3P = NA	Did not participate in activity	TENNIS
0	(PAC_3P = DK, R, NS)	Required question was not answered (don't know, refusal, not stated)	TENNIS
(PAC_2P × 4 × .2167 × 4) / 365	PAC_3P = 1	Calculate EE for < 15 min*	TENNIS
(PAC_2P × 4 × .3833 × 4) / 365	PAC_3P = 2	Calculate EE for 16 to 30 min*	TENNIS
(PAC_2P × 4 × .75 × 4) / 365	PAC_3P = 3	Calculate EE for 31 to 60 min*	TENNIS
(PAC_2P × 4 × 1 × 4) / 365	PAC_3P = 4	Calculate EE for > 60 min*	TENNIS
**PACDEEQ**			
0	PAC_3Q = NA	Did not participate in activity	WEIGHT-TRAINING
0	(PAC_3Q = DK, R, NS)	Required question was not answered (don't know, refusal, not stated)	WEIGHT-TRAINING
(PAC_2Q × 4 × .2167 × 3) / 365	PAC_3Q = 1	Calculate EE for < 15 min*	WEIGHT-TRAINING
(PAC_2Q × 4 × .3833 × 3) / 365	PAC_3Q = 2	Calculate EE for 16 to 30 min*	WEIGHT-TRAINING
(PAC_2Q × 4 × .75 × 3) / 365	PAC_3Q = 3	Calculate EE for 31 to 60 min*	WEIGHT-TRAINING
(PAC_2Q × 4 × 1 × 3) / 365	PAC_3Q = 4	Calculate EE for > 60 min*	WEIGHT-TRAINING
**PACDEER**			
0	PAC_3R = NA	Did not participate in activity	FISHING
0	(PAC_3R = DK, R, NS)	Required question was not answered (don't know, refusal, not stated)	FISHING
(PAC_2R × 4 × .2167 × 3) / 365	PAC_3R = 1	Calculate EE for < 15 min*	FISHING

(PAC_2R × 4 × .3833 × 3) / 365	PAC_3R = 2	Calculate EE for 16 to 30 min*	FISHING
(PAC_2R × 4 × .75 × 3) / 365	PAC_3R = 3	Calculate EE for 31 to 60 min*	FISHING
(PAC_2R × 4 × 1 × 3) / 365	PAC_3R = 4	Calculate EE for > 60 min*	FISHING
**PACDEES**			
0	PAC_3S = NA	Did not participate in activity	VOLLEYBALL
0	(PAC_3S = DK, R, NS)	Required question was not answered (don't know, refusal, not stated)	VOLLEYBALL
(PAC_2S × 4 × .2167 × 5) / 365	PAC_3S = 1	Calculate EE for < 15 min*	VOLLEYBALL
(PAC_2S × 4 × .3833 × 5) / 365	PAC_3S = 2	Calculate EE for 16 to 30 min*	VOLLEYBALL
(PAC_2S × 4 × .75 × 5) / 365	PAC_3S = 3	Calculate EE for 31 to 60 min*	VOLLEYBALL
(PAC_2S × 4 × 1 × 5) / 365	PAC_3S = 4	Calculate EE for > 60 min*	VOLLEYBALL
**PACDEET**			
0	PAC_3T = NA	Did not participate in activity	BASKETBALL
0	(PAC_3T = DK, R, NS)	Required question was not answered (don't know, refusal, not stated)	BASKETBALL
(PAC_2T × 4 × .2167 × 6) / 365	PAC_3T = 1	Calculate EE for < 15 min*	BASKETBALL
(PAC_2T × 4 × .3833 × 6) / 365	PAC_3T = 2	Calculate EE for 16 to 30 min*	BASKETBALL
(PAC_2T × 4 × .75 × 6) / 365	PAC_3T = 3	Calculate EE for 31 to 60 min*	BASKETBALL
(PAC_2T × 4 × 1 × 6) / 365	PAC_3T = 4	Calculate EE for > 60 min*	BASKETBALL
**PACDEEU**			
0	PAC_3U = NA	Did not participate in activity	OTHER (U)
0	(PAC_3U = DK, R, NS)	Required question was not answered (don't know, refusal, not stated)	OTHER (U)
(PAC_2U × 4 × .2167 × 4) / 365	PAC_3U = 1	Calculate EE for < 15 min*	OTHER (U)
(PAC_2U × 4 × .3833 × 4) / 365	PAC_3U = 2	Calculate EE for 16 to 30 min*	OTHER (U)
(PAC_2U × 4 × .75 × 4) / 365	PAC_3U = 3	Calculate EE for 31 to 60 min*	OTHER (U)
(PAC_2U × 4 × 1 × 4) / 365	PAC_3U = 4	Calculate EE for > 60 min*	OTHER (U)
**PACDEEW**			
0	PAC_3W = NA	Did not participate in activity	OTHER (W)
0	(PAC_3W = DK, R, NS)	Required question was not answered (don't know, refusal, not stated)	OTHER (W)
(PAC_2W × 4 × .2167 × 4) / 365	PAC_3W = 1	Calculate EE for < 15 min*	OTHER (W)
(PAC_2W × 4 × .3833 × 4) / 365	PAC_3W = 2	Calculate EE for 16 to 30 min*	OTHER (W)

(PAC_2W × 4 × .75 × 4) / 365	PAC_3W = 3	Calculate EE for 31 to 60 min*	OTHER (W)
(PAC_2W × 4 × 1 × 4) / 365	PAC_3W = 4	Calculate EE for > 60 min*	OTHER (W)
**PACDEEX**			
0	PAC_3X = NA	Did not participate in activity	OTHER (X)
0	(PAC_3X = DK, R, NS)	Required question was not answered (don’t know, refusal, not stated)	OTHER (X)
(PAC_2X × 4 × .2167 × 4) / 365	PAC_3X = 1	Calculate EE for < 15 min*	OTHER (X)
(PAC_2X × 4 × .3833 × 4) / 365	PAC_3X = 2	Calculate EE for 16 to 30 min*	OTHER (X)
(PAC_2X × 4 × .75 × 4) / 365	PAC_3X = 3	Calculate EE for 31 to 60 min*	OTHER (X)
(PAC_2X × 4 × 1 × 4) / 365	PAC_3X = 4	Calculate EE for > 60 min*	OTHER (X)
**PACDEEZ**			
0	PAC_3Z = NA	Did not participate in activity	SOCCER
0	(PAC_3Z = DK, R, NS)	Required question was not answered (don’t know, refusal, not stated)	SOCCER
(PAC_2Z × 4 × .2167 × 5) / 365	PAC_3Z = 1	Calculate EE for < 15 min*	SOCCER
(PAC_2Z × 4 × .3833 × 5) / 365	PAC_3Z = 2	Calculate EE for 16 to 30 min*	SOCCER
(PAC_2Z × 4 × .75 × 5) / 365	PAC_3Z = 3	Calculate EE for 31 to 60 min*	SOCCER
(PAC_2Z × 4 × 1 × 5) / 365	PAC_3Z = 4	Calculate EE for > 60 min*	SOCCER

Specifications

Value	Condition(s)	Description	Notes
99.9	ADM_PRX = 1	Module not asked - proxy interview	NS
99.9	(PAC_1V = DK, R, NS)	Required question was not answered (don't know, refusal, not stated)	NS
0	PAC_1V = 1	No leisure time physical activity	

PACDEEA + PACDEEB + PACDEEC + PACDEED + PACDEEE + PACDEEF + PACDEEG + PACDEEH + PACDEEI + PACDEEJ + PACDEEK + PACDEEL + PACDEEM + PACDEEN + PACDEEO + PACDEEP + PACDEEQ + PACDEER + PACDEES + PACDEET + PACDEEZ + PACDEEU + PACDEEW + PACDEEX	(0 <= PACDEEA < NA) and (0 <= PACDEEB < NA) and (0 <= PACDEEC < NA) and (0 <= PACDEED < NA) and (0 <= PACDEEE < NA) and (0 <= PACDEEF < NA) and (0 <= PACDEEG < NA) and (0 <= PACDEEH < NA) and (0 <= PACDEEI < NA) and (0 <= PACDEEJ < NA) and (0 <= PACDEEK < NA) and (0 <= PACDEEL < NA) and (0 <= PACDEEM < NA) and (0 <= PACDEEN < NA) and (0 <= PACDEEO < NA) and (0 <= PACDEEP < NA) and (0 <= PACDEEQ < NA) and (0 <= PACDEER < NA) and (0 <= PACDEES < NA) and (0 <= PACDEET < NA) and (0 <= PACDEEZ < NA) and (0 <= PACDEEU < NA) and (0 <= PACDEEW < NA) and (0 <= PACDEEX < NA)	Total daily energy expenditure (kcal/kg/day)	(rounded to one decimal place)<br><br>(min: 0.0; max: 99.5)

3) Participant In Leisure Time Physical Activity

Variable name: PACFLEI

Based on: PAC_1V

Description: This variable indicates whether the respondent participated in any leisure time physical activities in the three months prior to the interview.

Source: Ontario Health Survey

Internet site: www.chass.utoronto.ca/datalib/codebooks/utm/ohs/ohs90.htm

Specifications

Value	Condition(s)	Description	Notes
9	ADM_PRX = 1	Module not asked - proxy interview	NS
2	PAC_1V = 1	Does not participate in leisure time physical activity	
1	PAC_1V = 2	Participates in leisure time physical activity	
9	(PAC_1V = DK, R, NS)	Required question was not answered (don't know, refusal, not stated)	NS

4) Average Monthly Frequency of Leisure Time Physical Activity Lasting Over 15 Minutes

Variable name: PACDFM

Based on: PAC_1V, PAC_2A, PAC_2B, PAC_2C, PAC_2D, PAC_2E, PAC_2F, PAC_2G, PAC_2H, PAC_2I, PAC_2J, PAC_2K, PAC_2L, PAC_2M, PAC_2N, PAC_2O, PAC_2P, PAC_2Q, PAC_2R, PAC_2S, PAC_2T, PAC_2Z, PAC_2U, PAC_2W, PAC_2X, PAC_3A, PAC_3B, PAC_3C, PAC_3D, PAC_3E, PAC_3F, PAC_3G, PAC_3H, PAC_3I, PAC_3J, PAC_3K, PAC_3L, PAC_3M, PAC_3N, PAC_3O, PAC_3P, PAC_3Q, PAC_3R, PAC_3S, PAC_3T, PAC_3Z, PAC_3U, PAC_3W, PAC_3X

Description: This variable measures the total number of times per month that respondents took part in leisure time physical activity(ies) lasting more than 15 minutes.

Note: The survey questions refer to "the past three months". This variable calculates a one-month average by dividing the total reported frequency by three.

Source: Ontario Health Survey

Internet site: www.chass.utoronto.ca/datalib/codebooks/utm/ohs/ohs90.htm

Temporary Reformat

Value	Condition(s)	Description	Notes
**PACT2A**			
0	(PAC_3A = 1, NA, DK, R, NS)	Set all values for PAC_2A (number of times/3months respondents took part in physical activity) to 0 if PAC_3A is 1 (1 to 15 minutes), NA (did not participate in activity), or DK, R, NS (did not answer question)	
**PACT2B**			
0	(PAC_3B = 1, NA, DK, R, NS)	Set all values for PAC_2B (number of times/3months respondents took part in physical activity) to 0 if PAC_3B is 1 (1 to 15 minutes), NA (did not participate in activity), or DK, R, NS (did not answer question)	
**PACT2C**			
0	(PAC_3C = 1, NA, DK, R, NS)	Set all values for PAC_2C (number of times/3months respondents took part in physical activity) to 0 if PAC_3C is 1 (1 to 15 minutes), NA (did not participate in activity), or DK, R, NS (did not answer question)	
**PACT2D**			
0	(PAC_3D = 1, NA, DK, R, NS)	Set all values for PAC_2D (number of times/3months respondents took part in physical activity) to 0 if PAC_3D is 1 (1 to 15 minutes), NA (did not participate in activity), or DK, R, NS (did not answer question)	
**PACT2E**			
0	(PAC_3E = 1, NA, DK, R, NS)	Set all values for PAC_2E (number of times/3months respondents took part in physical activity) to 0 if PAC_3E is 1 (1 to 15 minutes), NA (did not participate in activity), or DK, R, NS (did not answer question)	
**PACT2F**			
0	(PAC_3F = 1, NA, DK, R, NS)	Set all values for PAC_2F (number of times/3months respondents took part in physical activity) to 0 if PAC_3F is 1 (1 to 15 minutes), NA (did not participate in activity), or DK, R, NS (did not answer question)	
**PACT2G**			
0	(PAC_3G = 1, NA, DK, R, NS)	Set all values for PAC_2G (number of times/3months respondents took part in physical activity) to 0 if PAC_3G is 1 (1 to 15 minutes), NA (did not participate in activity), or DK, R, NS (did not answer question)	
**PACT2H**			
0	(PAC_3H = 1, NA, DK, R, NS)	Set all values for PAC_2H (number of times/3months respondents took part in physical activity) to 0 if PAC_3H is 1 (1 to 15 minutes), NA (did not participate in activity), or DK, R, NS (did not answer question)	
**PACT2I**			

0	(PAC_3I = 1, NA, DK, R, NS)	Set all values for PAC_2I (number of times/3months respondents took part in physical activity) to 0 if PAC_3I is 1 (1 to 15 minutes), NA (did not participate in activity), or DK, R, NS (did not answer question)
**PACT2J**		
0	(PAC_3J = 1, NA, DK, R, NS)	Set all values for PAC_2J (number of times/3months respondents took part in physical activity) to 0 if PAC_3J is 1 (1 to 15 minutes), NA (did not participate in activity), or DK, R, NS (did not answer question)
**PACT2K**		
0	(PAC_3K = 1, NA, DK, R, NS)	Set all values for PAC_2K (number of times/3months respondents took part in physical activity) to 0 if PAC_3K is 1 (1 to 15 minutes), NA (did not participate in activity), or DK, R, NS (did not answer question)
**PACT2L**		
0	(PAC_3L = 1, NA, DK, R, NS)	Set all values for PAC_2L (number of times/3months respondents took part in physical activity) to 0 if PAC_3L is 1 (1 to 15 minutes), NA (did not participate in activity), or DK, R, NS (did not answer question)
**PACT2M**		
0	(PAC_3M = 1, NA, DK, R, NS)	Set all values for PAC_2M (number of times/3months respondents took part in physical activity) to 0 if PAC_3M is 1 (1 to 15 minutes), NA (did not participate in activity), or DK, R, NS (did not answer question)
**PACT2N**		
0	(PAC_3N = 1, NA, DK, R, NS)	Set all values for PAC_2N (number of times/3months respondents took part in physical activity) to 0 if PAC_3N is 1 (1 to 15 minutes), NA (did not participate in activity), or DK, R, NS (did not answer question)
**PACT2O**		
0	(PAC_3O = 1, NA, DK, R, NS)	Set all values for PAC_2O (number of times/3months respondents took part in physical activity) to 0 if PAC_3O is 1 (1 to 15 minutes), NA (did not participate in activity), or DK, R, NS (did not answer question)
**PACT2P**		
0	(PAC_3P = 1, NA, DK, R, NS)	Set all values for PAC_2P (number of times/3months respondents took part in physical activity) to 0 if PAC_3P is 1 (1 to 15 minutes), NA (did not participate in activity), or DK, R, NS (did not answer question)
**PACT2Q**		
0	(PAC_3Q = 1, NA, DK, R, NS)	Set all values for PAC_2Q (number of times/3months respondents took part in physical activity) to 0 if PAC_3Q is 1 (1 to 15 minutes), NA (did not participate in activity), or DK, R, NS (did not answer question)
**PACT2R**		
0	(PAC_3R = 1, NA, DK, R, NS)	Set all values for PAC_2R (number of times/3months respondents took part in physical activity) to 0 if PAC_3R is 1 (1 to 15 minutes), NA (did not participate in activity), or DK, R, NS (did not answer question)
**PACT2S**		

0	(PAC_3S = 1, NA, DK, R, NS)	Set all values for PAC_2S (number of times/3months respondents took part in physical activity) to 0 if PAC_3S is 1 (1 to 15 minutes), NA (did not participate in activity), or DK, R, NS (did not answer question)
**PACT2T**		
0	(PAC_3T = 1, NA, DK, R, NS)	Set all values for PAC_2T (number of times/3months respondents took part in physical activity) to 0 if PAC_3T is 1 (1 to 15 minutes), NA (did not participate in activity), or DK, R, NS (did not answer question)
**PACT2U**		
0	(PAC_3U = 1, NA, DK, R, NS)	Set all values for PAC_2U (number of times/3months respondents took part in physical activity) to 0 if PAC_3U is 1 (1 to 15 minutes), NA (did not participate in activity), or DK, R, NS (did not answer question)
**PACT2W**		
0	(PAC_3W = 1, NA, DK, R, NS)	Set all values for PAC_2W (number of times/3months respondents took part in physical activity) to 0 if PAC_3W is 1 (1 to 15 minutes), NA (did not participate in activity), or DK, R, NS (did not answer question)
**PACT2X**		
0	(PAC_3X = 1, NA, DK, R, NS)	Set all values for PAC_2X (number of times/3months respondents took part in physical activity) to 0 if PAC_3X is 1 (1 to 15 minutes), NA (did not participate in activity), or DK, R, NS (did not answer question)
**PACT2Z**		
0	(PAC_3Z = 1, NA, DK, R, NS)	Set all values for PAC_2Z (number of times/3months respondents took part in physical activity) to 0 if PAC_3Z is 1 (1 to 15 minutes), NA (did not participate in activity), or DK, R, NS (did not answer question)

Specifications

Value	Condition(s)	Description	Notes
999	ADM_PRX = 1	Module not asked - proxy interview	NS
999	(PAC_1V = DK, R, NS)	Required question was not answered (don’t know, refusal, not stated)	NS
0	PAC_1V=1	No leisure time physical activity	

(PACT2A +<br>PACT2B +<br>PACT2C +<br>PACT2D +<br>PACT2E +<br>PACT2F +<br>PACT2G +<br>PACT2H +<br>PACT2I +<br>PACT2J +<br>PACT2K +<br>PACT2L +<br>PACT2M +<br>PACT2N +<br>PACT2O +<br>PACT2P +<br>PACT2Q +<br>PACT2R +<br>PACT2S +<br>PACT2T +<br>PACT2Z +<br>PACT2U +<br>PACT2W +<br>PACT2X) / 3	(0 <= PACT2A < NA) and<br>(0 <= PACT2B < NA) and<br>(0 <= PACT2C < NA) and<br>(0 <= PACT2D < NA) and<br>(0 <= PACT2E < NA) and<br>(0 <= PACT2F < NA) and<br>(0 <= PACT2G < NA) and<br>(0 <= PACT2H < NA) and<br>(0 <= PACT2I < NA) and<br>(0 <= PACT2J < NA) and<br>(0 <= PACT2K < NA) and<br>(0 <= PACT2L < NA) and<br>(0 <= PACT2M < NA) and<br>(0 <= PACT2N < NA) and<br>(0 <= PACT2O < NA) and<br>(0 <= PACT2P < NA) and<br>(0 <= PACT2Q < NA) and<br>(0 <= PACT2R <NA) and<br>(0 <= PACT2S < NA) and<br>(0 <= PACT2T < NA) and<br>(0 <= PACT2Z < NA) and<br>(0 <= PACT2U < NA) and<br>(0 <= PACT2W < NA) and<br>(0 <= PACT2X < NA)	Monthly frequency of all leisure time physical activity lasting over 15 minutes	(Rounded to nearest integer)<br>(min: 0; max: 995)

5) Frequency of All Leisure Time Physical Activity Lasting Over 15 Minutes

Variable name: PACDFR

Based on: PACDFM

Description: This variable classifies respondents according to their pattern, or regularity of leisure time physical activity lasting more than 15 minutes.

Note: This variable uses values for the derived variable Monthly Frequency of Physical Activity (PACDFM). The values for PACDFM reflect a one-month average based on data reported for a three-month period.

Specifications

Value	Condition(s)	Description	Notes
9	ADM_PRX = 1	Module not asked - proxy interview	NS
9	PACDFM = NS	Required question was not answered (don’t know, refusal, not stated)	NS
1	(12 <= PACDFM < NA)	Regular practice of leisure time activities	
2	(4 <= PACDFM < 12)	Occasional practice of leisure time activities	
3	PACDFM < 4	Infrequent practice of leisure time activities	

6) Participant In Daily Leisure Time Physical Activity Lasting Over 15 Minutes

Variable name: PACFD

Based on: PACDFM

Description: This variable indicates whether the respondent participated daily in leisure time physical activity lasting over 15 minutes.

Note: This variable is based on values for Monthly Frequency of Physical Activity (PACDFM). Values for PACDFM reflect a one-month average based on data reported for a three-month period.

Specifications

Value	Condition(s)	Description	Notes
9	ADM_PRX = 1	Module not asked - proxy interview	NS
9	PACDFM = NS	At least one required question was not answered (don't know, refusal, not stated)	NS
1	(30 <= PACDFM < NA)	Participates in daily physical activity	
2	PACDFM < 30	Does not participate in daily physical activity	

7) Leisure Time Physical Activity Index

Variable name: PACDPAI

Based on: PACDEE

Description: This variable categorizes respondents as being "active", "moderately active", or "inactive" in their leisure time based on the total daily Energy Expenditure values (kcal/kg/day) calculated for PACDEE.

Note: The Physical Activity Index follows the same criteria used to categorize individuals in the Ontario Health Survey (OHS) and in the Campbell's Survey on Well Being.

Internet site: Campbell Survey on Well-Being in Canada: http://www.cflri.ca//pdf/e/88wkp.pdf

Specifications

Value	Condition(s)	Description	Notes
9	ADM_PRX = 1	Module not asked - proxy interview	NS
9	PACDEE = NS	At least one required question was not answered (don't know, refusal, not stated)	NS
1	(3 <= PACDEE < NA)	Active	
2	(1.5 <= PACDEE < 3.0)	Moderately active	
3	(0 <= PACDEE < 1.5)	Inactive	

8) Transportation and Leisure Time Physical Activity Index

Variable name: PACDLTI

Based on: PACDTLE

Description: This variable categorizes respondents as being "active", "moderately active", or "inactive" in their transportation and leisure time based on the total daily Energy Expenditure values (kcal/kg/day) calculated for PACDTLE.

Note: Transportation and Leisure Time Physical Activity Index follows the same criteria used in PACDPAI (Leisure Time Physical Activity Index).

Tansportation physical activity is not collected exclusively in CCHS. For this reason, collected information cannot be presented separately from the leisure time physical activities.

Specifications

Value	Condition(s)	Description	Notes
9	ADM_PRX = 1	Module not asked - proxy interview	NS
9	PACDTLE = NS	Required question was not answered (not stated)	NS
1	(3 <= PACDTLE < NA)	Active	
2	(1.5 <= PACDTLE < 3.0)	Moderately active	

3	(0 <= PACDTLE < 1.5)	Inactive

9) Daily Energy Expenditure in Transportation and Leisure Time Physical Activities

Variable name: PACDTLE

Based on: PACDEE, PAC_Q7, PAC_Q7A, PAC_Q7B, PAC_Q8, PAC_Q8A, PAC_Q8B

Description: This variable is a measure of the average daily energy expended during transportation and leisure time physical activities by the respondent in the past three months.

Note: For more information on how this derived variable is calculated, see note in PACDEE (Daily Energy Expenditure in Leisure Time Physical Activities).

Temporary Reformat

Value	Condition(s)	Description	Notes
**PACDTEA**			
0	PAC_7B = NA	Did not participate in transportation or leisure time physical activity	TRANSPORTATION - WALKING
0	(PAC_7B = DK, R, NS)	At least one required question was not answered (don’t know, refusal, not stated)	TRANSPORTATION - WALKING
(PAC_7A × 4 × .2167 × 3) / 365	PAC_7B = 1	Calculate EE for < 15 min*	TRANSPORTATION - WALKING
(PAC_7A × 4 × .3833 × 3) / 365	PAC_7B = 2	Calculate EE for 16 to 30 min*	TRANSPORTATION - WALKING
(PAC_7A × 4 × .75 × 3) / 365	PAC_7B = 3	Calculate EE for 31 to 60 min*	TRANSPORTATION - WALKING
(PAC_7A × 4 × 1 × 3) / 365	PAC_7B = 4	Calculate EE for > 60 min*	TRANSPORTATION - WALKING
**PACDTED**			
0	PAC_8B = NA	Did not participate in transportation or leisure time physical activity	TRANSPORTATION - BICYCLING
0	(PAC_8B = DK, R, NS)	At least one required question was not answered (don’t know, refusal, not stated)	TRANSPORTATION - BICYCLING
(PAC_8A × 4 × .2167 × 4) / 365	PAC_8B = 1	Calculate EE for < 15 min*	TRANSPORTATION - BICYCLING
(PAC_8A × 4 × .3833 × 4) / 365	PAC_8B = 2	Calculate EE for 16 to 30 min*	TRANSPORTATION - BICYCLING
(PAC_8A × 4 × .75 × 4) / 365	PAC_8B = 3	Calculate EE for 31 to 60 min*	TRANSPORTATION - BICYCLING
(PAC_8A × 4 × 1 × 4) / 365	PAC_8B = 4	Calculate EE for > 60 min*	TRANSPORTATION - BICYCLING

Specifications

Value	Condition(s)	Description	Notes
99.9	ADM_PRX = 1	Module not asked - proxy interview	NS
99.9	(PACDEE = DK, R, NS) or (PAC_7B = DK, R, NS) or (PAC_8B = DK, R, NS)	At least one required question was not answered (don't know, refusal, not stated)	NS
0	(PACDEE = 0) and (PAC_7 = 2, 3) and (PAC_8 = 2, 3)	No transportation or leisure time physical activity	

PACDEE + PACDTEA + PACDTED	(0 <= PACDEE < NA) and (0 <= PACDTEA < NA) and (0 <= PACDTED < NA)	Total daily energy expenditure (kcal/kg/day)	(rounded to one decimal place) (min: 0.0; max: 99.5)

10) Participant In Transportation or Leisure Time Physical Activity

Variable name: PACFLTI

Based on: PAC_1V, PAC_7, PAC_8

Description: This variable indicates whether the respondent participated in any transportation or leisure time physical activities in the three months prior to the interview.

Note: In 2010, the programming of the response categories for this derived variable were changed. Respondents who provided a mix of valid answer and non response to PAC_1V, PAC_7, or PAC_8 have been coded to category 1 or 2 in PACFLTI. Previously, if they provided a non response to either PAC_1V, PAC_7, or PAC_8 they were coded as non response in PACFLTI.

Specifications

Value	Condition(s)	Description	Notes
9	ADM_PRX = 1	Module not asked - proxy interview	NS
1	PAC_1V = 2 or PAC_7 = 1 or PAC_8 = 1	Participates in transportation or leisure time physical activity	
2	(PAC_1V = 1) and (PAC_7 = 2, 3) and (PAC_8 = 2, 3)	Does not participate in transportation or leisure time physical activity	
9	(PAC_1V = DK, R, NS) or (PAC_7 = DK, R, NS) or (PAC_8 = DK, R, NS)	Required question was not answered (don't know, refusal, not stated)	NS

Physical activities - Facilities at work (1 DV)

1) Access to Physical Activity Facilities at Work

Variable name: PAFFACC

Based on: PAF_01, PAF_02, PAF_03, PAF_04, PAF_05, PAF_06, PAF_07, PAF_08

Description: This variable identifies whether respondents have access to physical activity facilities at or near their place of work.

Specifications

Value	Condition(s)	Description	Notes
6	DHH_AGE < 15 or DHH_AGE > 75 or (LBS_01 = 2 and LBS_02 = 2) or LBS_01 = 3	Population exclusion	NA
6	DOPAF = 2	Module not selected	NA
1	(PAF_02 = 1) or (PAF_03 = 1) or (PAF_04 = 1) or (PAF_05 = 1) or (PAF_06 = 1) or (PAF_07 = 1) or (PAF_08 = 1)	Has access to physical activity facilities at or near place of work	
2	[(PAF_02 = 2) and (PAF_03 = 2) and (PAF_04 = 2) and (PAF_05 = 2) and (PAF_06 = 2) and (PAF_07 = 2) and (PAF_08 = 2)] or [(PAF_01 = 1) and (PAF_02 = 2) and (PAF_03 = 2) and (PAF_04 = 2) and (PAF_05 = 2)]	No access to physical activity facilities at or near place of work	
9	(LBS_01 = DK, R, NS) or (LBS_02 = DK, R, NS) or (PAF_02 = DK, R, NS) or (PAF_03 = DK, R, NS) or (PAF_04 = DK, R, NS) or (PAF_05 = DK, R, NS) or (PAF_06 = DK, R, NS) or (PAF_07 = DK, R, NS) or (PAF_08 = DK, R, NS)	At least one required question was not answered (don't know, refusal, not stated)	NS

Psychological well-being (1 DV)

1) Psychological Well-Being Manifestation Scale (WBMMS)

Variable name: PWBDPWB

Based on: PWB_01, PWB_02, PWB_03, PWB_04, PWB_05, PWB_06, PWB_07, PWB_08, PWB_09, PWB_10, PWB_11, PWB_12, PWB_13, PWB_14, PWB_15, PWB_16, PWB_17, PWB_18, PWB_19, PWB_20, PWB_21, PWB_22, PWB_23, PWB_24, PWB_25

Description: This variable assesses the level of psychological well-being of the respondent.

Note: 1) The scale is base on questions proposed by Raymond Massé (Université Laval). The scale is discussed in the reference presented below.
2) Higher scores indicate greater well-being.

Temporary Reformat

Value	Condition(s)	Description	Notes
**PWBT01**			
(5 - PWB_01)	PWB_01 <= 5	Reverse code all responses so that higher scores reflect higher levels of well-being.<br><br>Change the scale from 1-5 to 0-4 so the summed scale will range from 0 to 100.	
**PWBT02**			
(5 - PWB_02)	PWB_02 <= 5	Reverse code all responses so that higher scores reflect higher levels of well-being.<br><br>Change the scale from 1-5 to 0-4 so the summed scale will range from 0 to 100.	
**PWBT03**			
(5 - PWB_03)	PWB_03 <= 5	Reverse code all responses so that higher scores reflect higher levels of well-being.<br><br>Change the scale from 1-5 to 0-4 so the summed scale will range from 0 to 100.	
**PWBT04**			
(5 - PWB_04)	PWB_04 <= 5	Reverse code all responses so that higher scores reflect higher levels of well-being.<br><br>Change the scale from 1-5 to 0-4 so the summed scale will range from 0 to 100.	
**PWBT05**			
(5 - PWB_05)	PWB_05 <= 5	Reverse code all responses so that higher scores reflect higher levels of well-being.<br><br>Change the scale from 1-5 to 0-4 so the summed scale will range from 0 to 100.	
**PWBT06**			
(5 - PWB_06)	PWB_06 <= 5	Reverse code all responses so that higher scores reflect higher levels of well-being.<br><br>Change the scale from 1-5 to 0-4 so the summed scale will range from 0 to 100.	
**PWBT07**			

(5 - PWB_07)	PWB_07 <= 5	Reverse code all responses so that higher scores reflect higher levels of well-being.<br><br>Change the scale from 1-5 to 0-4 so the summed scale will range from 0 to 100.
**PWBT08**		
(5 - PWB_08)	PWB_08 <= 5	Reverse code all responses so that higher scores reflect higher levels of well-being.<br><br>Change the scale from 1-5 to 0-4 so the summed scale will range from 0 to 100.
**PWBT09**		
(5 - PWB_09)	PWB_09 <= 5	Reverse code all responses so that higher scores reflect higher levels of well-being.<br><br>Change the scale from 1-5 to 0-4 so the summed scale will range from 0 to 100.
**PWBT10**		
(5 - PWB_10)	PWB_10 <= 5	Reverse code all responses so that higher scores reflect higher levels of well-being.<br><br>Change the scale from 1-5 to 0-4 so the summed scale will range from 0 to 100.
**PWBT11**		
(5 - PWB_11)	PWB_11 <= 5	Reverse code all responses so that higher scores reflect higher levels of well-being.<br><br>Change the scale from 1-5 to 0-4 so the summed scale will range from 0 to 100.
**PWBT12**		
(5 - PWB_12)	PWB_12 <= 5	Reverse code all responses so that higher scores reflect higher levels of well-being.<br><br>Change the scale from 1-5 to 0-4 so the summed scale will range from 0 to 100.
**PWBT13**		
(5 - PWB_13)	PWB_13 <= 5	Reverse code all responses so that higher scores reflect higher levels of well-being.<br><br>Change the scale from 1-5 to 0-4 so the summed scale will range from 0 to 100.
**PWBT14**		
(5 - PWB_14)	PWB_14 <= 5	Reverse code all responses so that higher scores reflect higher levels of well-being.<br><br>Change the scale from 1-5 to 0-4 so the summed scale will range from 0 to 100.
**PWBT15**		
(5 - PWB_15)	PWB_15 <= 5	Reverse code all responses so that higher scores reflect higher levels of well-being.<br><br>Change the scale from 1-5 to 0-4 so the summed scale will range from 0 to 100.
**PWBT16**		
(5 - PWB_16)	PWB_16 <= 5	Reverse code all responses so that higher scores reflect higher levels of well-being.<br><br>Change the scale from 1-5 to 0-4 so the summed scale will range from 0 to 100.
**PWBT17**		

(5 - PWB_17)	PWB_17 <= 5	Reverse code all responses so that higher scores reflect higher levels of well-being.<br><br>Change the scale from 1-5 to 0-4 so the summed scale will range from 0 to 100.	
**PWBT18**			
(5 - PWB_18)	PWB_18 <= 5	Reverse code all responses so that higher scores reflect higher levels of well-being.<br><br>Change the scale from 1-5 to 0-4 so the summed scale will range from 0 to 100.	
**PWBT19**			
(5 - PWB_19)	PWB_19 <= 5	Reverse code all responses so that higher scores reflect higher levels of well-being.<br><br>Change the scale from 1-5 to 0-4 so the summed scale will range from 0 to 100.	
**PWBT20**			
(5 - PWB_20)	PWB_20 <= 5	Reverse code all responses so that higher scores reflect higher levels of well-being.<br><br>Change the scale from 1-5 to 0-4 so the summed scale will range from 0 to 100.	
**PWBT21**			
(5 - PWB_21)	PWB_21 <= 5	Reverse code all responses so that higher scores reflect higher levels of well-being.<br><br>Change the scale from 1-5 to 0-4 so the summed scale will range from 0 to 100.	
**PWBT22**			
(5 - PWB_22)	PWB_22 <= 5	Reverse code all responses so that higher scores reflect higher levels of well-being.<br><br>Change the scale from 1-5 to 0-4 so the summed scale will range from 0 to 100.	
**PWBT23**			
(5 - PWB_23)	PWB_23 <= 5	Reverse code all responses so that higher scores reflect higher levels of well-being.<br><br>Change the scale from 1-5 to 0-4 so the summed scale will range from 0 to 100.	
**PWBT24**			
(5 - PWB_24)	PWB_24 <= 5	Reverse code all responses so that higher scores reflect higher levels of well-being.<br><br>Change the scale from 1-5 to 0-4 so the summed scale will range from 0 to 100.	
**PWBT25**			
(5 - PWB_25)	PWB_25 <= 5	Reverse code all responses so that higher scores reflect higher levels of well-being.<br><br>Change the scale from 1-5 to 0-4 so the summed scale will range from 0 to 100.	

Specifications

Value	Condition(s)	Description	Notes
996	DOPWB = 2	Module not selected	NA
999	ADM_PRX = 1	Module not asked - proxy interview	NS

999	(PWB_01 = DK, R, NS) or<br>(PWB_02 = DK, R, NS) or<br>(PWB_03 = DK, R, NS) or<br>(PWB_04 = DK, R, NS) or<br>(PWB_05 = DK, R, NS) or<br>(PWB_06 = DK, R, NS) or<br>(PWB_07 = DK, R, NS) or<br>(PWB_08 = DK, R, NS) or<br>(PWB_09 = DK, R, NS) or<br>(PWB_10 = DK, R, NS) or<br>(PWB_11 = DK, R, NS) or<br>(PWB_12 = DK, R, NS) or<br>(PWB_13 = DK, R, NS) or<br>(PWB_14 = DK, R, NS) or<br>(PWB_15 = DK, R, NS) or<br>(PWB_16 = DK, R, NS) or<br>(PWB_17 = DK, R, NS) or<br>(PWB_18 = DK, R, NS) or<br>(PWB_19 = DK, R, NS) or<br>(PWB_20 = DK, R, NS) or<br>(PWB_21 = DK, R, NS) or<br>(PWB_22 = DK, R, NS) or<br>(PWB_23 = DK, R, NS) or<br>(PWB_24 = DK, R, NS) or<br>(PWB_25 = DK, R, NS)	At least one required question was not answered (don’t know, refusal, not stated)	NS
PWBT01 +<br>PWBT02 +<br>PWBT03 +<br>PWBT04 +<br>PWBT05 +<br>PWBT06 +<br>PWBT07 +<br>PWBT08 +<br>PWBT09 +<br>PWBT10 +<br>PWBT11 +<br>PWBT12 +<br>PWBT13 +<br>PWBT14 +<br>PWBT15 +<br>PWBT16 +<br>PWBT17 +<br>PWBT18 +<br>PWBT19 +<br>PWBT20 +<br>PWBT21 +<br>PWBT22 +<br>PWBT23 +<br>PWBT24 +<br>PWBT25	PWB_01 <= 5 and<br>PWB_02 <= 5 and<br>PWB_03 <= 5 and<br>PWB_04 <= 5 and<br>PWB_05 <= 5 and<br>PWB_06 <= 5 and<br>PWB_07 <= 5 and<br>PWB_08 <= 5 and<br>PWB_09 <= 5 and<br>PWB_10 <= 5 and<br>PWB_11 <= 5 and<br>PWB_12 <= 5 and<br>PWB_13 <= 5 and<br>PWB_14 <= 5 and<br>PWB_15 <= 5 and<br>PWB_16 <= 5 and<br>PWB_17 <= 5 and<br>PWB_18 <= 5 and<br>PWB_19 <= 5 and<br>PWB_20 <= 5 and<br>PWB_21 <= 5 and<br>PWB_22 <= 5 and<br>PWB_23 <= 5 and<br>PWB_24 <= 5 and<br>PWB_25 <= 5	Score obtained on the psychological well-being scale	(min: 0; max: 100)

Reference: "Élaboration et validation d’un outil de mesure du bien-être psychologique: L’ÉMMBEP" R. Massé, C. Poulin, C. Dassa, J. Lambert, S. Bélair, M.A. Battaglini. Revue Canadienne de Santé Publique, Vol. 89. No. 5, pp. 352-357.

Restriction of activities (3 DVs)

1) Cause of health problem

Variable name: RACG5

Based on: RAC_5

Description: This variable indicates the cause of the respondent's health problem. It is a regrouping of RAC_5.

Specifications

Value	Condition(s)	Description	Notes
NA	RAC_5 = NA	Not applicable	
NS	RAC_5 = DK, R or NS	Not stated	
1	1 <= RAC_5 <= 4	Injury (includes injury at home, sports or recreation, motor vehicle, work related)	
2	RAC_5 = 7	Disease or illness	
3	RAC_5 = 8	Aging	
4	RAC_5 = 5	Existed at birth or genetic	
5	RAC_5 = 6	Work conditions	
6	RAC_5 = 9 or<br>RAC_5 = 10 or<br>RAC_5 = 11	Other(psychological/physical abuse, use of alcohol or drugs, other)	

2) Impact of Health Problems

Variable name: RACDIMP

Based on: RAC_2A, RAC_2B1, RAC_2B2, RAC_2C

Description: This variable is a crude measure of the impact of long-term physical conditions, mental conditions and health problems on the principal domains of life: home, work, school, and other activities.

Note: This variable should not be used to describe the rate of disability or activity limitation in the population. The questions used to derive this variable, plus RAC_1, were asked in the 2006 Census of Population to identify a sample for the 2006 post-censal Participation and Activity Limitation Survey (PALS).

Specifications

Value	Condition(s)	Description	Notes
2	RAC_2A = 2 or<br>RAC_2B1 = 2 or<br>RAC_2B2 = 2 or<br>RAC_2C = 2	Often	
1	RAC_2A = 1 or<br>RAC_2B1 = 1 or<br>RAC_2B2 = 1 or<br>RAC_2C = 1	Sometimes	
3	RAC_2A = 3 and<br>(RAC_2B1 = 3, 4) and<br>(RAC_2B2 = 3, 4) and<br>RAC_2C = 3	Never	

9	(RAC_2A = DK, R, NS) or<br>(RAC_2B1 = DK, R, NS) or<br>(RAC_2B2 = DK, R, NS) or<br>(RAC_2C = DK, R, NS)	At least one required question was not answered (don't know, refusal, not stated)	NS

3) Participation and Activity Limitation

Variable name: RACDPAL

Based on: RAC_1, RAC_2A, RAC_2B1, RAC_2B2, RAC_2C

Description: This variable classifies respondents according to the frequency with which they experience activity limitations imposed on them by a condition(s) or by long-term physical and/or mental health problems that has lasted or is expected to last 6 months or more.

Note: This variable is the same as RACDIMP with the exception that RAC_1 is used in the calculation. This variable is a modification of the Participation and Activity Limitation Survey (PALS) derived variables. Whereas PALS treats non-response (DK, R) as a negative response (set to "Never"), CCHS treats them as non-response and the derived variable is set to not-stated.

Specifications

Value	Condition(s)	Description	Notes
9	(RAC_2A = DK, R, NS) or<br>(RAC_2B1 = DK, R, NS) or<br>(RAC_2B2 = DK, R, NS) or<br>(RAC_2C = DK, R, NS) or<br>(RAC_1 = DK, R, NS)	At least one required question was not answered (don't know, refusal, not stated)	NS
2	RAC_2A = 2 or<br>RAC_2B1 = 2 or<br>RAC_2B2 = 2 or<br>RAC_2C = 2 or<br>RAC_1 = 2	Often	
1	RAC_2A = 1 or<br>RAC_2B1 = 1 or<br>RAC_2B2 = 1 or<br>RAC_2C = 1 or<br>RAC_1 = 1	Sometimes	
3	RAC_2A = 3 and<br>(RAC_2B1 = 3, 4) and<br>(RAC_2B2 = 3, 4) and<br>RAC_2C = 3 and<br>RAC_1 = 3	Never	

Repetitive strain injuries (1 DV)

1) Repetitive strain injury

Variable name: REPG3

Based on: REP_3

Description: This variable indicates the body part affected by the repetitive strain injury. It is a regrouping of REP_3.

Specifications

Value	Condition(s)	Description	Notes
NA	REP_3 = NA	Not applicable	
NS	REP_3 = NS	Respondent did not answer (don't know, refusal, not specified)	
1	REP_3 = 2	Neck	
2	REP_3 = 3	Shoulder/upper arm	
3	REP_3 = 4	Elbow/lower arm	
4	REP_3 = 5	Wrist/hand	
5	REP_3 = 8	Knee/lower leg	
6	REP_3 = 9	Ankle/foot	
7	REP_3 = 10	Upper back/upper spine	
8	REP_3 = 11	Lower back/lower spine	
9	REP_3 = 1 or REP_3 = 6 or REP_3 = 7 or REP_3 = 12, REP_3 = 13	Other(includes head, hip, thigh, chest, abdomen or pelvis)	

Sedentary activities (6 DVs)

1) Number of hours - on a computer - past 3 mo - (G)

Variable name: SACG1

Based on: SAC_1

Description: This variables indicates how much time the respondent, in a typical week in the past 3 months, spends on a computer, including playing computer games and using the Internet.

Note: Includes only leisure time activities. This grouped variable is very similar to the SAC_1 variable produced previous to 2009 (where the number of hours was given using answer categories instead of precise value).

Specifications

Value	Condition(s)	Description	Notes
96	SAC_1 = 96	Population exclusions	NA
99	SAC_1 in (97, 98, 99)	At least one required question was not answered (don’t know, refusal, not stated)	NS
1	SAC_1 = 0	None or less than 1 hour	
2	SAC_1 in (1, 2)	From 1 ot 2 hours	
3	SAC_1 in (3, 4, 5)	From 3 to 5 hours	
4	SAC_1 in (6, 7, 8, 9, 10)	From 6 to 10 hours	
5	SAC_1 in (11, 12, 13, 14)	From 11 to 14 hours	
6	SAC_1 in (15, 16, 17, 18, 19, 20)	From 15 to 20 hours	
7	SAC_1 >= 21	More than 20 hours	

2) Number of hours - playing video games - past 3 mo - (G)

Variable name: SACG2

Based on: SAC_2

Description: This variables indicates how much time the respondent, in a typical week in the past 3 months, spends playing video games, such as XBOX, Nintendo and Playstation.

Note: Includes only leisure time activities. This grouped variable is very similar to the SAC_2 variable produced previous to 2009 (where the number of hours was given using answer categories instead of precise value).

Specifications

Value	Condition(s)	Description	Notes
96	SAC_2 = 96	Population exclusions	NA
99	SAC_2 in (97, 98, 99)	At least one required question was not answered (don’t know, refusal, not stated)	NS
1	SAC_2 = 0	None or less than 1 hour	
2	SAC_2 in (1, 2)	From 1 ot 2 hours	
3	SAC_2 in (3, 4, 5)	From 3 to 5 hours	
4	SAC_2 in (6, 7, 8, 9, 10)	From 6 to 10 hours	
5	SAC_2 in (11, 12, 13, 14)	From 11 to 14 hours	

6	SAC_2 in (15, 16, 17, 18, 19, 20)	From 15 to 20 hours
7	SAC_2 >= 21	More than 20 hours

3) Number of hours - watching tv/videos - past 3 mo - (G)

Variable name: SACG3

Based on: SAC_3

Description: This variables indicates how much time the respondent, in a typical week in the past 3 months, spends watching television or videos.

Note: Includes only leisure time activities. This grouped variable is very similar to the SAC_3 variable produced previous to 2009 (where the number of hours was given using answer categories instead of precise value).

Specifications

Value	Condition(s)	Description	Notes
96	SAC_3 = 96	Population exclusions	NA
99	SAC_3 in (97, 98, 99)	At least one required question was not answered (don't know, refusal, not stated)	NS
1	SAC_3 = 0	None or less than 1 hour	
2	SAC_3 in (1, 2)	From 1 ot 2 hours	
3	SAC_3 in (3, 4, 5)	From 3 to 5 hours	
4	SAC_3 in (6, 7, 8, 9, 10)	From 6 to 10 hours	
5	SAC_3 in (11, 12, 13, 14)	From 11 to 14 hours	
6	SAC_3 in (15, 16, 17, 18, 19, 20)	From 15 to 20 hours	
7	SAC_3 in (21, 22, 23, 24, 25)	From 21 to 25 hours	
8	SAC_3 in (26, 27, 28, 29, 30)	From 26 to 30 hours	
9	SAC_3 >= 31	More than 30 hours	

4) Number of hours - reading - past 3 mo - (G)

Variable name: SACG4

Based on: SAC_4

Description: This variables indicates how much time the respondent, in a typical week in the past 3 months, spends reading, not counting at work or at school.

Note: Includes only leisure time activities. This grouped variable is very similar to the SAC_4 variable produced previous to 2009 (where the number of hours was given using answer categories instead of precise value).

Specifications

Value	Condition(s)	Description	Notes
96	SAC_4 = 96	Population exclusions	NA
99	SAC_4 in (97, 98, 99)	At least one required question was not answered (don't know, refusal, not stated)	NS
1	SAC_4 = 0	None or less than 1 hour	
2	SAC_4 in (1, 2)	From 1 ot 2 hours	

3	SAC_4 in (3, 4, 5)	From 3 to 5 hours
4	SAC_4 in (6, 7, 8, 9, 10)	From 6 to 10 hours
5	SAC_4 in (11, 12, 13, 14)	From 11 to 14 hours
6	SAC_4 in (15, 16, 17, 18, 19, 20)	From 15 to 20 hours
7	SAC_4 >= 21	More than 20 hours

5) Total Number of Hours Per Week Spent In Sedentary Activities

Variable name: SACDTOT

Based on: SAC_1, SAC_2, SAC_3, SAC_4

Description: This variable estimates the total number of hours the respondent spent in a typical week in the past three months in sedentary activities including using a computer (including playing computer games), using the Internet, playing video games (e.g. Nintendo, PlayStation) (for respondents aged 25 or less), watching television or videos and reading. For all activities, the time spent at school or work is excluded.

Temporary Reformat

Value	Condition(s)	Description	Notes
**SAC**			
996	SAC_1 = NA	Population exclusion	NA
999	ADM_PRX = 1	Module not asked - proxy interview	NS
999	(SAC_1 = DK, R, NS) or (SAC_2 = DK, R, NS) or (SAC_3 = DK, R, NS) or (SAC_4 = DK, R, NS)	At least one required question was not answered (don't know, refusal, not stated)	NS
SAC_1+SAC_2+ SAC_3+SAC_4	(0 <= SAC_1 <= 70) and (0 <= SAC_2 <= 70) and (0 <= SAC_3 <= 70) and (0 <= SAC_4 <= 70)	Total number of hours spent in sedentary activities where the respondent is aged <= 25	
SAC_1+SAC_3+ SAC_4	(0 <= SAC_1 <= 70) and SAC_2 = NA and (0 <= SAC_3 <= 70) and (0 <= SAC_4 <= 70)	Total number of hours spent in sedentary activities where respondent is aged >25	

Specifications

Value	Condition(s)	Description	Notes
96	SAC = NA	Module not selected	NA
99	SAC = NS	Module not asked - proxy interview	NS
99	SAC = NS	At least one required question was not answered (don't know, refusal, not stated)	NS
1	(0 <= SAC < 5)	Less than 5 hours	
2	(5 <= SAC < 10)	From 5 to 9 hours	
3	(10 <= SAC < 15)	From 10 to 14 hours	
4	(15 <= SAC < 20)	From 15 to 19 hours	
5	(20 <= SAC < 25)	From 20 to 24 hours	
6	(25 <= SAC < 30)	From 25 to 29 hours	
7	(30 <= SAC < 35)	From 30 to 34 hours	

8	(35 <= SAC < 40)	From 35 to 39 hours
9	(40 <= SAC < 45)	From 40 to 44 hours
10	(45 <= SAC < NA)	More than 45 hours

6) Total number of hours per week spent in sedentary activities (excluding reading)

Variable name: SACDTER

Based on: SAC_1, SAC_2, SAC_3

Description: This variable estimates the total number of hours the respondent spent in a typical week in the past three months in sedentary activities including using a computer (including playing computer games), using the Internet, playing video games (e.g. Nintendo, PlayStation)(for respondents aged less than 25), and watching television or videos. For all activities, the time spent at school or work is excluded. Time spent in reading is not included.

Temporary Reformat

Value	Condition(s)	Description	Notes
**SACTTER**			
996	SAC_1 = NA	Population exclusions	NA
999	ADM_PRX = 1	Module not asked - proxy interview	NS
999	(SAC_1 = DK, R, NS) or (SAC_2 = DK, R, NS) or (SAC_3 = DK, R, NS)	At least one required question was not answered (don't know, refusal, not stated)	NS
SAC_1 + SAC_2 + SAC_3	(0 <= SAC_1 <= 70) and (0 <= SAC_2 <= 70) and (0 <= SAC_3 <= 70)	Total number of hours per week spent in sedentary activities (excluding reading) where the respondent is aged <= 25	
SAC_1 + SAC_3	(0 <= SAC_1 <= 70) and (0 <= SAC_3 <= 70)	Total number of hours per week spent in sedentary activities (excluding reading) where the respondent is aged > 25	

Specifications

Value	Condition(s)	Description	Notes
96	SACTTER = NA	Population exclusion	NA
99	SACTTER = NS	Module not asked - proxy interview or at least one required question was not answered (don't know, refusal, not stated)	NS
1	(0 <= SACTTER < 5)	Less than 5 hours	
2	(5 <= SACTTER < 10)	From 5 to 9 hours	
3	(10 <= SACTTER < 15)	From 10 to 14 hours	
4	(15 <= SACTTER < 20)	From 15 to 19 hours	
5	(20 <= SACTTER < 25)	From 20 to 24 hours	
6	(25 <= SACTTER < 30)	From 25 to 29 hours	
7	(30 <= SACTTER < 35)	From 30 to 34 hours	
8	(35 <= SACTTER < 40)	From 35 to 39 hours	
9	(40 <= SACTTER < 45)	From 40 to 44 hours	
10	(45 <= SACTTER < NA)	45 hours or more	

Smoking - Stages of change (1 DV)

The stages of change model defines five stages of change in the process of smoking cessation:

1) Precontemplation - The person has no intention of changing behaviour in the foreseeable future (for example, quitting smoking).
2) Contemplation - The person is aware of the problem and is seriously thinking about changing the behaviour but has not yet made a commitment to take action or is not confident of being able to sustain the behavioural change (that is, seriously thinking of quitting in the next 30 days but did not try to quit for at least 24 hours in the past 12 months, or seriously thinking of quitting smoking in the next 6 months but not in the next 30 days).
3) Preparation - The person is seriously planning to take action in the next month and is confident of success (that is, seriously thinking of quitting smoking in the next 30 days and has already stopped smoking at least once during the past 12 months).
4) Action - The person has successfully modified the behaviour within the past 6 months (that is, has quit smoking less than six months ago).
5) Maintenance - The person has maintained the behaviour change for at least six months (that is, has quit smoking at least six months ago).

1) Smoking Stages of Change (Current and Former Smokers)

Variable name: SCHDSTG

Based on: SMK_202, SMK_06A, SMK_06B, SMK_09A, SMK_09B, SMK_10, SMK_10A, SMK_10B, SCH_1, SCH_2, SCH_3, SCH_4, ADM_MOI

Description: This variable classifies current and former smokers into categories based on the stages of change model.

Specifications

Value	Condition(s)	Description	Notes
6	DOSCH= 2	Module not selected	NA
6	SMK_202 = 3 and<br>SMK_01A = 2	Population exclusion	NA
9	ADM_PRX = 1	Module not asked - proxy interview	NS
1	(SMK_202 = 1, 2) and<br>SCH_1 = 2	Precontemplation stage<br>(Current daily or occasional smokers)	
2	(SMK_202 = 1, 2) and<br>[(SCH_1 = 1 and<br>SCH_2 = 2) or<br>(SCH_2 = 1 and<br>SCH_3 = 2)]	Contemplation stage<br>(Current daily or occasional smokers)	
3	(SMK_202 = 1, 2) and<br>SCH_2 = 1 and<br>(1 <= SCH_4 <= 95)	Preparation stage<br>(Current daily or occasional smokers)	
4	SMK_202 = 3 and<br>(SMK_06B < 6 months from ADM_MOI)<br>or<br>SMK_202 = 3 and SMK_10 = 1 and<br>(SMK_09B < 6 months from ADM_MOI)<br>or<br>SMK_202 = 3 and<br>(SMK_10B < 6 months from ADM_MOI)	Action stage<br>(Former smoker)<br><br>Assesses whether respondent has stopped smoking within 6 months prior to completing survey	
5	SMK_202 = 3 and<br>[(SMK_06A = 2, 3, 4) or<br>(SMK_06B >= 6 months from ADM_MOI)]<br>or<br>SMK_202 = 3 and<br>SMK_10 = 1 and<br>[(SMK_9A = 2, 3, 4) or<br>(SMK_09B >= 6 months from ADM_MOI)]<br>or<br>SMK_202 = 3 and<br>[(SMK_10A = 2, 3, 4) or<br>(SMK_10B >= 6 months from ADM_MOI)]	Maintenance stage<br>(Former smoker)<br><br>Assesses whether respondent stopped smoking 6 months or more prior to completing survey	

9	(SMK_202 = DK, R, NS) or<br>(SMK_06B = DK, R, NS) or<br>(SMK_09B = DK, R, NS) or<br>(SMK_10 = DK, R, NS) or<br>(SMK_10B = DK, R, NS) or<br>(SCH_1 = DK, R, NS) or<br>(SCH_2 = DK, R, NS) or<br>(SCH_3 = DK, R, NS) or<br>(SCH_4 = DK, R, NS)	At least one required question was not answered (don't know, refusal, not stated)	NS

Reference: DiClemente, C.C., Prochaska, J.O., Fairhurst, S., Velicer, W.F., Rossi J.S., & Velasquez, M. (1991). The process of smoking cessation: An analysis of precontemplation, contemplation and contemplation/action. Journal of Consulting and Clinical Psychology, 59, 295-304.

Socio-demographic characteristics (6 DVs)

1) Country of Birth - Grouped

Variable name: SDCGCBG

Based on: SDCCCB

Description: This variable classifies the respondent based on his/her country of birth.

Specifications

Value	Condition(s)	Description	Notes
9	(SDCCCB = 000, DK, R, NS)	Required question was not answered (don't know, refusal, not stated)	NS
1	(0 < SDCCCB < 14)	Canada	
2	(100 <= SDCCCB < 900)	Other	

2) Language(s) spoken at home - (D, G) - Grouped

Variable name: SDCGLHM

Based on: SDC_5AA, SDC_5AB, SDC_5AC, SDC_5AD, SDC_5AE, SDC_5AF, SDC_5AG, SDC_5AH, SDC_5AI, SDC_5AJ, SDC_5AK, SDC_5AL, SDC_5AM, SDC_5AN, SDC_5AO, SDC_5AP, SDC_5AQ, SDC_5AR, SDC_5AS, SDC_5AT, SDC_5AU, SDC_5AV, SDC_5AW

Description: This variable indicates the language(s) in which the respondent converses at home.

Specifications

Value	Condition(s)	Description	Notes
99	(SDC_5AA = DK, R, NS)	Required question was not answered (don't know, refusal, not stated)	NS
1	(SDC_5AA = 1 and SDC_5AB > 1)	English (with or without language other than French)	
2	(SDC_5AA > 1 and SDC_5AB = 1)	French (with or without language other than English)	
3	(SDC_5AA = 1 and SDC_5AB = 1)	English & French (with or without other language)	

4	(SDC_5AA > 1 and SDC_5AB > 1) and (SDC_5AC = 1 or SDC_5AD = 1 or SDC_5AE = 1 or SDC_5AF = 1 or SDC_5AG = 1 or SDC_5AH = 1 or SDC_5AI = 1 or SDC_5AJ = 1 or SDC_5AK = 1 or SDC_5AL = 1 or SDC_5AM = 1 or SDC_5AN = 1 or SDC_5AO = 1 or SDC_5AP = 1 or SDC_5AQ = 1 or SDC_5AR = 1 or SDC_5AS = 1 or SDC_5AT = 1 or SDC_5AU = 1 or SDC_5AV = 1 or SDC_5AW = 1)	Neither English nor French (Other)	

3) Immigration flag

Variable name: SDCFIMM

Based on: SDC_3

Description: This variable indicates if the respondent is an immigrant.

Specifications

Value	Condition(s)	Description	Notes
9	(SDC_3 = DK, R, NS)	Required question was not answered (don’t know, refusal, not stated)	NS
1	SDC_3 < NA	Immigrant	
2	SDC_3 = NA	Not an immigrant	

4) Length of time in Canada since immigration - Grouped

Variable name: SDCGRES

Based on: SDC_3, ADM_YOI

Description: This variable indicates the length of time the respondent’s been in Canada since his/her immigration.

Note: Non immigrants were excluded from the population.
ADM_MOI = Month of Interview (unpublished)

Specifications

Value	Condition(s)	Description	Notes
996	SDC_3 = NA	Population exclusions	NA
999	(SDC_3 = DK, R, NS)	Required question was not answered (don’t know, refusal, not stated)	NS

1	ADM_YOI (current year) - SDC_3 (SDC_3 < NA)	Length of time in Canada since immigration 0 - 9 years are grouped together	(min: 0; max: 9)
2	ADM_YOI (current year) - SDC_3 (SDC_3 < NA)	Length of time in Canada since immigration 10 - 130 years are grouped together	(min: 10; max: 130)

5) Language(s) in which respondent can converse - Grouped

Variable name: SDCGLNG

Based on: SDC_5A, SDC_5B, SDC_5C, SDC_5D, SDC_5E, SDC_5F, SDC_5G, SDC_5H, SDC_5I, SDC_5J, SDC_5K, SDC_5L, SDC_5M, SDC_5N, SDC_5O, SDC_5P, SDC_5Q, SDC_5R, SDC_5S, SDC_5T, SDC_5U, SDC_5V, SDC_5W

Description: This variable indicates the language(s) in which the respondent can converse.

Specifications

Value	Condition(s)	Description	Notes
99	(SDC_5A = DK, R, NS)	Required question was not answered (don't know, refusal, not stated)	NS
1	(SDC_5A = 1 and SDC_5B > 1)	English (with or without language other than French)	
2	(SDC_5A > 1 and SDC_5B = 1)	French (with or without language other than English)	
3	(SDC_5A = 1 and SDC_5B = 1)	English & French (with or without other language)	
4	(SDC_5A > 1 and SDC_5B > 1) and (SDC_5C = 1 or SDC_5D = 1 or SDC_5E = 1 or SDC_5F = 1 or SDC_5G = 1 or SDC_5H = 1 or SDC_5I = 1 or SDC_5J = 1 or SDC_5K = 1 or SDC_5L = 1 or SDC_5M = 1 or SDC_5N = 1 or SDC_5O = 1 or SDC_5P = 1 or SDC_5Q = 1 or SDC_5R = 1 or SDC_5S = 1 or SDC_5T = 1 or SDC_5U = 1 or SDC_5V = 1 or SDC_5W = 1)	Neither English nor French (Other)	

6) Culture / Race Flag - Grouped

Variable name: SDCGCGT

Based on: SDCDCGT, SDC_41

Description: This variable indicates the cultural or racial origin of the respondent.

Specifications

Value	Condition(s)	Description	Notes
9	(SDC_41 = DK, R, NS)	Required question was not answered (don't know,	NS

	(SDCDCGT = DK, R, NS)	refusal, not stated)
1	SDCDCGT= 1	White
2	SDCDCGT= 2 or<br>SDCDCGT= 3 or<br>SDCDCGT= 4 or<br>SDCDCGT= 5 or<br>SDCDCGT= 6 or<br>SDCDCGT= 7 or<br>SDCDCGT= 8 or<br>SDCDCGT= 9 or<br>SDCDCGT= 10 or<br>SDCDCGT= 11 or<br>SDCDCGT= 12 or<br>SDCDCGT= 13 or<br>SDC_41 = 1	Non-white (Aboriginal or Other Visible Minority)

Self-esteem (1 DV)

Temporary Reformat

Value	Condition(s)	Description	Notes
**SFET501**			
SFE_501	SFE_501 > 5	Carry through cases of RF, DK, NS	
(5 - SFE_501)	SFE_501 <= 5	Invert and rescale the question answers from 1 - 5 to 4 - 0	
**SFET502**			
SFE_502	SFE_502 > 5	Carry through cases of RF, DK, NS	
(5 - SFE_502)	SFE_502 <= 5	Invert and rescale the question answers from 1 - 5 to 4 - 0	
**SFET503**			
SFE_503	SFE_503 > 5	Carry through cases of RF, DK, NS	
(5 - SFE_503)	SFE_503 <= 5	Invert and rescale the question answers from 1 - 5 to 4 - 0	
**SFET504**			
SFE_504	SFE_504 > 5	Carry through cases of RF, DK, NS	
(5 - SFE_504)	SFE_504 <= 5	Invert and rescale the question answers from 1 - 5 to 4 - 0	
**SFET505**			
SFE_505	SFE_505 > 5	Carry through cases of RF, DK, NS	
(5 - SFE_505)	SFE_505 <= 5	Invert and rescale the question answers from 1 - 5 to 4 - 0	
**SFET506**			
SFE_506	SFE_506 > 5	Carry through cases of RF, DK, NS	
(SFE_506 - 1)	SFE_506 <= 5	Rescale the question answers	

1) Derived Self-Esteem Scale

Variable name: SFEDE1

Based on: SFE_501, SFE_502, SFE_503, SFE_504, SFE_505, SFE_506

Description: This variable assesses the level of self-esteem (positive feeling) an individual has.

Note: Scores on the index are based on a subset of items from the self-esteem Rosenberg scale (1969). The six items have been factored into one dimension in the factor analysis done by Pearlin and Schooler (1978).
Higher scores indicate greater self-esteem.

Specifications

Value	Condition(s)	Description	Notes
96	DOSFE = 2	Module not selected	NA
99	ADM_PRX = 1	Module not asked - proxy interview	NS
99	(SFET501 = DK, R, NS) or<br>(SFET502 = DK, R, NS) or<br>(SFET503 = DK, R, NS) or<br>(SFET504 = DK, R, NS) or<br>(SFET505 = DK, R, NS) or<br>(SFET506 = DK, R, NS)	At least one required question was not answered (don’t know, refusal, not stated)	NS

SFET501 +<br>SFET502 +<br>SFET503 +<br>SFET504 +<br>SFET505 +<br>SFET506	(0 <= SFET501 <= 4) and<br>(0 <= SFET502 <= 4) and<br>(0 <= SFET503 <= 4) and<br>(0 <= SFET504 <= 4) and<br>(0 <= SFET505 <= 4) and<br>(0 <= SFET506 <= 4)	Score obtained on the self-esteem scale	(min: 0; max: 24)

Reference: Rosenberg, Morris, Conceiving the self, appendix A, 1979, pp. 291-295.

Health status (SF-36) (10 DVs)

The 36-item short form (SF-36) of the Medical Outcomes Study questionnaire was designed as a generic indicator of health status for use in population surveys and evaluative studies of health policy. The SF-36 was developed by John E. Ware Jr., Institute for the Improvement of Medical Care and Health, New England Medical Center Hospitals. The items in the SF-36 were drawn from the original 245-item Medical Outcomes Study (MOS). The SF-36 includes multi-item scales to measure the following three major health attributes and eight health concepts:

Functional Status
- Physical Functioning
- Social Functioning
- Role Limitations attributed to Physical Problems
- Role Limitations attributed to Emotional Problems

Well-Being
- Mental Health
- Energy (vitality)
- Bodily Pain

Overall Evaluation of Health
- General Health Perception

A scale is calculated for each of the eight health concepts. All scales are scored so that a high score is consistent with a positive health status. For example, a "functioning" scale is scored so that a higher score reflects increased function.

In order to facilitate comparisons across the SF-36 scales, the raw scores for each scale are linearly transformed to a 0-to-100 scale using the formula:

Transformed scale = [(Actual score - Lowest possible score) / Possible score range] X 100

The transformed score reflects a relative position of the respondent on a continuum of lowest to highest possible scale scores.

Two summary measures of physical and mental health are also constructed from the eight scales.

Temporary Reformat

Value	Condition(s)	Description	Notes
**SFRT01**			
1	GEN_01 = 5	Rescale responses required to create the eight health concept scales	
2.0	GEN_01 = 4	Rescale responses required to create the eight health concept scales	
3.4	GEN_01 = 3	Rescale responses required to create the eight health concept scales	
4.4	GEN_01 = 2	Rescale responses required to create the eight health concept scales	
5	GEN_01 = 1	Rescale responses required to create the eight health concept scales	
**SFRT20**			
(6 – SFR_20)	All	Rescale responses required to create the eight health concept scales	
**SFRT21**			
1	SFR_21 = 6	Rescale responses required to create the eight health concept scales	
2.2	SFR_21 = 5	Rescale responses required to create the eight health concept scales	
3.1	SFR_21 = 4	Rescale responses required to create the eight health concept scales	
4.2	SFR_21 = 3	Rescale responses required to create the eight health concept scales	
5.4	SFR_21 = 2	Rescale responses required to create the eight health concept scales	
6	SFR_21 = 1	Rescale responses required to create the eight health concept scales	
**SFRT22**			
1	SFR_22 = 5 and (1 <= SFR_21 <= 6)	Rescale responses required to create the eight health concept scales	

2	SFR_22 = 4 and<br>(1 <= SFR_21 <= 6)	Rescale responses required to create the eight health concept scales
3	SFR_22 = 3 and<br>(1 <= SFR_21 <= 6)	Rescale responses required to create the eight health concept scales
4	SFR_22 = 2 and<br>(1 <= SFR_21 <= 6)	Rescale responses required to create the eight health concept scales
5	SFR_22 = 1 and<br>(2 <= SFR_21 <= 6)	Rescale responses required to create the eight health concept scales
6	SFR_22 = 1 and<br>SFR_21 = 1	Rescale responses required to create the eight health concept scales
**SFRT23**		
(7 – SFR_23)	All	Rescale responses required to create the eight health concept scales
**SFRT26**		
(7 – SFR_26)	All	Rescale responses required to create the eight health concept scales
**SFRT27**		
(7 – SFR_27)	All	Rescale responses required to create the eight health concept scales
**SFRT30**		
(7 – SFR_30)	All	Rescale responses required to create the eight health concept scales
**SFRT34**		
(6 – SFR_34)	All	Rescale responses required to create the eight health concept scales
**SFRT36**		
(6 – SFR_36)	All	Rescale responses required to create the eight health concept scales

1) Physical Functioning Scale

Variable name: SFRDPFS

Based on: SFR_03, SFR_04, SFR_05, SFR_06, SFR_07, SFR_08, SFR_09, SFR_10, SFR_11, SFR_12

Description: This variable measures the level of physical functioning of the respondent relative to the general population.

Note: A high score reflects increased physical function.

Specifications

Value	Condition(s)	Description	Notes
996	DOSFR = 2	Module not selected	NA
999	(SFR_03 = DK, R, NS) or<br>(SFR_04 = DK, R, NS) or<br>(SFR_05 = DK, R, NS) or<br>(SFR_06 = DK, R, NS) or<br>(SFR_07 = DK, R, NS) or<br>(SFR_08 = DK, R, NS) or<br>(SFR_09 = DK, R, NS) or<br>(SFR_10 = DK, R, NS) or<br>(SFR_11 = DK, R, NS) or<br>(SFR_12 = DK, R, NS)	At least one required question was not answered (don’t know, refusal, not stated)	NS

100*[(SFR_03 + SFR_04 + SFR_05 + SFR_06 + SFR_07 + SFR_08 + SFR_09 + SFR_10 + SFR_11 + SFR_12) - 10] / 20	(1 <= SFR_03 <= 3) and (1 <= SFR_04 <= 3) and (1 <= SFR_05 <= 3) and (1 <= SFR_06 <= 3) and (1 <= SFR_07 <= 3) and (1 <= SFR_08 <= 3) and (1 <= SFR_09 <= 3) and (1 <= SFR_10 <= 3) and (1 <= SFR_11 <= 3) and (1 <= SFR_12 <= 3)	Score obtained on the physical functioning scale	(min: 0; max: 100)

2) Social Functioning Scale

Variable name: SFRDSFS

Based on: SFR_20, SFR_32

Description: This variable measures the level of social functioning of the respondent relative to the general population.

Note: A high score reflects increased social functioning.

Specifications

Value	Condition(s)	Description	Notes
996	DOSFR = 2	Module not selected	NA
999	(SFR_20 = DK, R, NS) or (SFR_32 = DK, R, NS)	At least one required question was not answered (don’t know, refusal, not stated)	NS
100*[(SFRT20 + SFR_32) - 2] / 9	(1 <= SFRT20 <= 5) and (1 <= SFR_32 <= 6)	Score obtained on the social functioning scale	(min: 0; max: 100)

3) Role Functioning (Physical) Scale

Variable name: SFRDPRF

Based on: SFR_13, SFR_14, SFR_15, SFR_16

Description: This variable measures the role limitations due to physical health problems for the respondent relative to the general population.

Note: A high score reflects increased physical function (ie., less limitation).

Specifications

Value	Condition(s)	Description	Notes
996	DOSFR = 2	Module not selected	NA
999	(SFR_13 = DK, R, NS) or (SFR_14 = DK, R, NS) or (SFR_15 = DK, R, NS) or (SFR_16 = DK, R, NS)	At least one required question was not answered (don’t know, refusal, not stated)	NS
100*[(SFR_13 + SFR_14 + SFR_15 + SFR_16) - 4] / 4	(1 <= SFR_13 <= 2) and (1 <= SFR_14 <= 2) and (1 <= SFR_15 <= 2) and (1 <= SFR_16 <= 2)	Score obtained on the role functioning (physical) scale	(min: 0; max: 100)

4) Role Functioning (Mental) Scale

Variable name: SFRDMRF

Based on: SFR_17, SFR_18, SFR_19

Description: This variable measures the mental role functioning of the respondent relative to the general population.

Note: A high score is consistent with a positive mental health status.

Specifications

Value	Condition(s)	Description	Notes
996	DOSFR = 2	Module not selected	NA
999	(SFR_17 = DK, R, NS) or<br>(SFR_18 = DK, R, NS) or<br>(SFR_19 = DK, R, NS)	At least one required question was not answered (don’t know, refusal, not stated)	NS
100*[(SFR_17 + SFR_18 + SFR_19) - 3] / 3	(1 <= SFR_17 <= 2) and<br>(1 <= SFR_18 <= 2) and<br>(1 <= SFR_19 <= 2)	Score obtained on the role functioning (mental) scale	(min: 0; max: 100)

5) General Mental Health Scale

Variable name: SFRDGMH

Based on: SFR_24, SFR_25, SFR_26, SFR_28, SFR_30

Description: This variable indicates the general mental health of people in the general population.

Note: The scale is transformed to facilitate comparisons across scales and reflect a relative position. A high score is consistent with a positive general mental health status.

Specifications

Value	Condition(s)	Description	Notes
996	DOSFR = 2	Module not selected	NA
999	(SFR_24 = DK, R, NS) or<br>(SFR_25 = DK, R, NS) or<br>(SFR_26 = DK, R, NS) or<br>(SFR_28 = DK, R, NS) or<br>(SFR_30 = DK, R, NS)	At least one required question was not answered (don’t know, refusal, not stated)	NS
100*[(SFR_24 + SFR_25 + SFRT26 + SFR_28 + SFRT30) - 5] / 25	(1 <= SFR_24 <= 6) and<br>(1 <= SFR_25 <= 6) and<br>(1 <= SFRT26 <= 6) and<br>(1 <= SFR_28 <= 6) and<br>(1 <= SFRT30 <= 6)	Score obtained on the general mental health scale	(min: 0; max: 100)

6) Vitality Scale

Variable name: SFRDVTS

Based on: SFR_23, SFR_27, SFR_29, SFR_31

Description: This variable indicates a measure of energy (vitality) of the respondent relative to the general population.

Note: A high score is consistent with a positive level of energy.

Specifications			
**Value**	**Condition(s)**	**Description**	**Notes**
996	DOSFR = 2	Module not selected	NA
999	(SFR_23 = DK, R, NS) or (SFR_27 = DK, R, NS) or (SFR_29 = DK, R, NS) or (SFR_31 = DK, R, NS)	At least one required question was not answered (don't know, refusal, not stated)	NS
100*[(SFRT23 + SFRT27 + SFR_29 + SFR_31) - 4] / 20	(1 <= SFRT23 <= 6) and (1 <= SFRT27 <= 6) and (1 <= SFR_29 <= 6) and (1 <= SFR_31 <= 6)	Score obtained on the vitality scale	(min: 0; max: 100)

7) Bodily Pain Scale

Variable name: SFRDBPS

Based on: SFR_21, SFR_22

Description: This variable indicates a measure of bodily pain experienced by the respondent relative to the general population.

Note: A high score is consistent with a decreased level of pain.

Specifications			
**Value**	**Condition(s)**	**Description**	**Notes**
996	DOSFR = 2	Module not selected	NA
999	(SFRT21 = DK, R, NS) or (SFRT22 = DK, R, NS)	At least one required question was not answered (don't know, refusal, not stated)	NS
100*((SFRT21 + SFRT22) - 2) / 10	(1 <= SFRT21 <= 6) and (1 <= SFRT22 <= 6)	Score obtained on the bodily pain scale	(min: 0; max: 100)

8) General Health Perceptions Scale

Variable name: SFRDGHP

Based on: SFR_01, SFR_33, SFR_34, SFR_35, SFR_36

Description: This variable indicates the general health perceptions of the respondent relative to the general population.

Note: A high score is consistent with a positive perception of one's general health status.

Temporary Reformat			
**Value**	**Condition(s)**	**Description**	**Notes**
**SFRDBPST**			
(SFRDBPS - 75.49196) / 23.55879		Reformat the eight health concept scales to calculate two summary measures of physical and mental health	
**SFRDGHPT**			

(SFRDGHP - 72.21316) / 20.16964	Reformat the eight health concept scales to calculate two summary measures of physical and mental health
**SFRDGMHT**	
(SFRDGMH - 74.84212) / 18.01189	Reformat the eight health concept scales to calculate two summary measures of physical and mental health
**SFRDMRFT**	
(SFRDMRF - 81.29467) / 33.02717	Reformat the eight health concept scales to calculate two summary measures of physical and mental health
**SFRDPFST**	
(SFRDPFS - 84.52404) / 22.89490	Reformat the eight health concept scales to calculate two summary measures of physical and mental health
**SFRDPRFT**	
(SFRDPRF - 81.19907) / 33.79729	Reformat the eight health concept scales to calculate two summary measures of physical and mental health
**SFRDSFST**	
(SFRDSFS - 83.59753) / 22.37642	Reformat the eight health concept scales to calculate two summary measures of physical and mental health
**SFRDVTST**	
(SFRDVTS - 61.05453) / 20.86942	Reformat the eight health concept scales to calculate two summary measures of physical and mental health

Specifications

Value	Condition(s)	Description	Notes
996	DOSFR = 2	Module not selected	NA
999	(SFRT01 = DK, R, NS) or (SFR_33 = DK, R, NS) or (SFR_34 = DK, R, NS) or (SFR_35 = DK, R, NS) or (SFR_36 = DK, R, NS)	At least one required question was not answered (don’t know, refusal, not stated)	NS
100*[(SFRT01 + SFR_33 + SFRT34 + SFR_35 + SFRT36) - 5] / 20	(1 <= SFRT01 <= 5) and (1 <= SFR_33 <= 5) and (1 <= SFRT34 <= 5) and (1 <= SFR_35 <= 5) and (1 <= SFRT36 <= 5)	Score obtained on the general health perception scale	(min: 0; max: 100)

9) Summary Measure of Physical Health

Variable name: SFRDPCS

Based on: SFRDPFS, SFRDSFS, SFRDPRF, SFRDMRF, SFRDGMH, SFRDVTS, SFRDBPS, SFRDGHP

Description: This variable is a summary measure of physical health that is constructed from the eight health concept scales (physical functioning, social functioning, role limitation-physical, role limitation-mental, general mental health, vitality, bodily pain, general health perceptions).

Specifications

Value	Condition(s)	Description	Notes
96	DOSFR = 2	Module not selected	NA
99	SFRDPFS = NS or<br>SFRDSFS = NS or<br>SFRDPRF = NS or<br>SFRDMRF = NS or<br>SFRDGMH = NS or<br>SFRDVTS = NS or<br>SFRDBPS = NS or<br>SFRDGHP = NS	At least one required question was not answered (don't know, refusal, not stated)	NS
[((SFRDPFST * .42402) + (SFRDSFST * -.00753) + (SFRDPRFT * .35119) + (SFRDMRFT * -.19206) + (SFRDGMHT * -.22069) + (SFRDVTST * .02877) + (SFRDBPST * .31754) + (SFRDGHPT * .24954)) * 10] + 50	SFRDPFS <> NS and<br>SFRDSFS <> NS and<br>SFRDPRF <> NS and<br>SFRDMRF <> NS and<br>SFRDGMH <> NS and<br>SFRDVTS <> NS and<br>SFRDBPS <> NS and<br>SFRDGHP <> NS	Summary measure of physical health	(min: 8; max 68)

10) Summary Measure of Mental Health

Variable name: SFRDMCS

Based on: SFRDPFS, SFRDSFS, SFRDPRF, SFRDMRF, SFRDGMH, SFRDVTS, SFRDBPS, SFRDGHP

Description: This variable is a summary measure of mental health that is constructed from the eight health concept scales (physical functioning, social functioning, role limitation-physical, role limitation-mental, general mental health, vitality, bodily pain, general health perceptions).

Specifications

Value	Condition(s)	Description	Notes
96	DOSFR = 2	Module not selected	NA
99	SFRDPFS = NS or<br>SFRDSFS = NS or<br>SFRDPRF = NS or<br>SFRDMRF = NS or<br>SFRDGMH = NS or<br>SFRDVTS = NS or<br>SFRDBPS = NS or<br>SFRDGHP = NS	At least one required question was not answered (don't know, refusal, not stated)	NS

[((SFRDPFST * -.22999) + (SFRDSFST * .26876) + (SFRDPRFT * -.12329) + (SFRDMRFT * .43407) + (SFRDGMHT * .48581) + (SFRDVTST * .23534) + (SFRDBPST * -.09731) + (SFRDGHPT * -.01571)) * 10] + 50	SFRDPFS <> NS and SFRDSFS <> NS and SFRDPRF <> NS and SFRDMRF <> NS and SFRDGMH <> NS and SFRDVTS <> NS and SFRDBPS <> NS and SFRDGHP <> NS	Summary measure of mental health	(min: 3; max: 74)

Smoking (3 DVs)

1) Type of Smoker

Variable name: SMKDSTY

Based on: SMK_01A, SMK_01B, SMK_202, SMK_05D

Description: This variable indicates the type of smoker the respondent is, based on his/her smoking habits.

Note: This variable includes lifetime cigarette consumption.

Specifications

Value	Condition(s)	Description	Notes
1	SMK_202 = 1	Daily smoker	
2	SMK_202 = 2 and SMK_05D = 1	Occasional smoker (former daily smoker)	
3	SMK_202 = 2 and (SMK_05D = 2, NA)	Occasional smoker (never a daily smoker or has smoked less than 100 cigarettes lifetime)	
4	SMK_202 = 3 and SMK_05D = 1	Former daily smoker (non-smoker now)	
5	SMK_202 = 3 and [[SMK_05D = 2 or SMK_05D = 6] and [SMK_01A = 1 or SMK_01B = 1]]	Former occasional smoker (at least 1 whole cigarette, non-smoker now)	
6	SMK_202 = 3 and SMK_01A = 2 and SMK_01B = 2	Never smoked (a whole cigarette)	
99	(SMK_01A = DK, R, NS) or (SMK_01B = DK, R, NS) or (SMK_202 = DK, R, NS) or (SMK_05D = DK, R, NS)	At least one required question was not answered (don't know, refusal, not stated)	NS

Reference:
In 2010, the programming of the response categories for this derived variable was changed. Respondents who stated that they were non-smokers, did not smoke more than 100 cigarettes, but have smoked a whole cigarette (SMK_202=3, SMK_05D=5, SMK_01A=2, and SMK_01B=1) were being classified as not stated (SMKDSTY=99) and should have been classified former occasional smokers (at least 1 whole cigarette, non-smoker now)(SMKDSTY=5). Programming was adjusted to ensure that the category was being assigned correctly to all cases.

2) Number of Years Since Stopping Smoking Completely - Grouped

Variable name: SMKGSTP

Based on: SMK_06A, SMK_06C, SMK_09A, SMK_09C, SMK_10, SMK_10A, SMK_10C, SMKDSTY

Description: This variable indicates the approximate number of years since former smokers completely quit smoking.

Note: Current smokers and respondents who have never smoked a whole cigarette and respondents who did not smoked a total of 100 cigarettes or more lifetime were excluded from the population.

Specifications

Value	Condition(s)	Description	Notes
6	(SMKDSTY = 1, 2, 3, 6) or (SMK_202 = 3 and SMK_01A = 2 and	Population exclusions	NA

	SMK_01B = 1)		
9	SMKDSTY = NS or<br>(SMK_10 = DK, R, NS) or<br>(SMK_06A = DK, R, NS) or<br>(SMK_06C = DK, R, NS) or<br>(SMK_09A = DK, R, NS) or<br>(SMK_09C = DK, R, NS) or<br>(SMK_10A = DK, R, NS) or<br>(SMK_10C = DK, R, NS)	At least one required question was not answered (don't know, refusal, not stated)	NS
1	SMK_06A = 1 or<br>(SMK_10 = 1 and<br>SMK_09A = 1) or<br>SMK_10A = 1	Less than 1 year	
2	[SMK_06A = 2 or<br>(SMK_10 = 1 and<br>SMK_09A = 2) or<br>SMK_10A = 2]<br>or<br>[SMK_06A = 3 or<br>(SMK_10 = 1 and<br>SMK_09A = 3) or<br>SMK_10A = 3]	1 to 2 years	
3	[SMK_06A = 4 and<br>(3 =< SMK_06C < 6)]<br>or<br>[SMK_09A = 4 and<br>SMK_10 = 1 and<br>(3 =< SMK_09C < 6)]<br>or<br>[SMK_10A = 4 and<br>(3 =< SMK_10C < 6)]	3 to 5 years	
4	[SMK_06A = 4 and<br>(6 =< SMK_06C < 11)]<br>or<br>[SMK_09A = 4 and<br>SMK_10 = 1 and<br>(6 =< SMK_09C < 11)]<br>or<br>[SMK_10A=4 and<br>(6 =< SMK_10C < 11)]	6 to 10 years	
5	[SMK_06A = 4 and<br>(11 =< SMK_06C < 126)]<br>or<br>[SMK_09A = 4 and<br>SMK_10 = 1 and<br>(11 =< SMK_09C < 126)]<br>or<br>[SMK_10A = 4 and<br>(11 =< SMK_10C < 126)]	11 or more years	

3) Number of Years Smoked Daily (Current Daily Smokers Only)

Variable name: SMKDYCS

Based on: SMK_202, SMK_203, DHH_AGE

Description: This variable indicates the number of years the respondent has smoked daily.

Note: Respondents who are not daily smokers have been excluded from the population. The NPHS variables includes non-smokers and occasional smokers who previously smoked daily.

Specifications

Value	Condition(s)	Description	Notes
996	(SMK_202 = 2, 3)	Population exclusion	NA
999	(SMK_202 = DK, R, NS) or<br>(SMK_203 = DK, R, NS)	At least one required question was not answered (don’t know, refusal, not stated)	NS
DHH_AGE - SMK_203	SMK_202 = 1	Number of years smoked daily	(min: 0; max: 125)

Social support - Availability (4 DVs)

The Medical Outcomes Study (MOS) Social Support Survey provides indicators of four categories of Social Support. An initial pool of 50 items was reduced to 19 functional support items that were hypothesized to cover five dimensions:

- Emotional support - the expression of positive affect, empathetic understanding, and the encouragement of expressions of feelings.
- Informational support - the offering of advice, information, guidance or feedback.
- Tangible support - the provision of material aid or behavioural assistance.
- Positive social interaction - the availability of other persons to do fun things with you.
- Affection - involving expressions of love and affection.

Empirical analysis indicated that emotional and informational support items should be scored together, so 4 subscales are derived:

- Tangible social support (questions 2, 5, 12, 15)
- Affection (questions 6, 10, 20)
- Positive social interaction (questions 7, 11, 14, 18)
- Emotional or informational support (question 3, 4, 8, 9, 13, 16, 17, 19)

Temporary Reformat

Value	Condition(s)	Description	Notes
**SSAT02**			
SSA_02	SSA_02 > 5	Carry through cases of RF, DK, NS	
(SSA_02 - 1)	SSA_02 <= 5	Rescale the answers from 1 to 5 to 0 to 4<br>Where 0 is “never” and 4 is “always”	
**SSAT03**			
(SSA_03 - 1)	SSA_03 <= 5	Rescale the answers from 1 to 5 to 0 to 4<br>Where 0 is “never” and 4 is “always”	
SSA_03	SSA_03 > 5	Carry through cases of RF, DK, NS	
**SSAT04**			
(SSA_04 - 1)	SSA_04 <= 5	Rescale the answers from 1 to 5 to 0 to 4<br>Where 0 is “never” and 4 is “always”	
SSA_04	SSA_04 > 5	Carry through cases of RF, DK, NS	
**SSAT05**			
SSA_05	SSA_05 > 5	Carry through cases of RF, DK, NS	
(SSA_05 - 1)	SSA_05 <= 5	Rescale the answers from 1 to 5 to 0 to 4<br>Where 0 is “never” and 4 is “always”	
**SSAT06**			
SSA_06	SSA_06 > 5	Carry through cases of RF, DK, NS	
(SSA_06 - 1)	SSA_06 <= 5	Rescale the answers from 1 to 5 to 0 to 4<br>Where 0 is “never” and 4 is “always”	
**SSAT07**			
SSA_07	SSA_07 > 5	Carry through cases of RF, DK, NS	
(SSA_07 - 1)	SSA_07 <= 5	Rescale the answers from 1 to 5 to 0 to 4<br>Where 0 is “never” and 4 is “always”	
**SSAT08**			
SSA_08	SSA_08 > 5	Carry through cases of RF, DK, NS	
(SSA_08 - 1)	SSA_08 <= 5	Rescale the answers from 1 to 5 to 0 to 4<br>Where 0 is “never” and 4 is “always”	
**SSAT09**			
SSA_09	SSA_09 > 5	Carry through cases of RF, DK, NS	
(SSA_09 - 1)	SSA_09 <= 5	Rescale the answers from 1 to 5 to 0 to 4<br>Where 0 is “never” and 4 is “always”	

**SSAT10**		
SSA_10	SSA_10 > 5	Carry through cases of RF, DK, NS
(SSA_10 - 1)	SSA_10 <= 5	Rescale the answers from 1 to 5 to 0 to 4<br>Where 0 is “never” and 4 is “always”
**SSAT11**		
SSA_11	SSA_11 > 5	Carry through cases of RF, DK, NS
(SSA_11 - 1)	SSA_11 <= 5	Rescale the answers from 1 to 5 to 0 to 4<br>Where 0 is “never” and 4 is “always”
**SSAT12**		
SSA_12	SSA_12 > 5	Carry through cases of RF, DK, NS
(SSA_12 - 1)	SSA_12 <= 5	Rescale the answers from 1 to 5 to 0 to 4<br>Where 0 is “never” and 4 is “always”
**SSAT13**		
(SSA_13 - 1)	SSA_13 <= 5	Rescale the answers from 1 to 5 to 0 to 4<br>Where 0 is “never” and 4 is “always”
SSA_13	SSA_13 > 5	Carry through cases of RF, DK, NS
**SSAT14**		
(SSA_14 - 1)	SSA_14 <= 5	Rescale the answers from 1 to 5 to 0 to 4<br>Where 0 is “never” and 4 is “always”
SSA_14	SSA_14 > 5	Carry through cases of RF, DK, NS
**SSAT15**		
(SSA_15 - 1)	SSA_15 <= 5	Rescale the answers from 1 to 5 to 0 to 4<br>Where 0 is “never” and 4 is “always”
SSA_15	SSA_15 > 5	Carry through cases of RF, DK, NS
**SSAT16**		
(SSA_16 - 1)	SSA_16 <= 5	Rescale the answers from 1 to 5 to 0 to 4<br>Where 0 is “never” and 4 is “always”
SSA_16	SSA_16 > 5	Carry through cases of RF, DK, NS
**SSAT17**		
(SSA_17 - 1)	SSA_17 <= 5	Rescale the answers from 1 to 5 to 0 to 4<br>Where 0 is “never” and 4 is “always”
SSA_17	SSA_17 > 5	Carry through cases of RF, DK, NS
**SSAT18**		
(SSA_18 - 1)	SSA_18 <= 5	Rescale the answers from 1 to 5 to 0 to 4<br>Where 0 is “never” and 4 is “always”
SSA_18	SSA_18 > 5	Carry through cases of RF, DK, NS
**SSAT19**		
(SSA_19 - 1)	SSA_19 <= 5	Rescale the answers from 1 to 5 to 0 to 4<br>Where 0 is “never” and 4 is “always”
SSA_19	SSA_19 > 5	Carry through cases of RF, DK, NS
**SSAT20**		
SSA_20	SSA_20 > 5	Carry through cases of RF, DK, NS
(SSA_20 - 1)	SSA_20 <= 5	Rescale the answers from 1 to 5 to 0 to 4<br>Where 0 is “never” and 4 is “always”

1) Tangible Social Support - MOS Subscale

Variable name: SSADTNG

Based on: SSA_02, SSA_05, SSA_12, SSA_15

Description: This variable measures the level of tangible support that is available to the respondent. Questions about whether or not the respondent had someone to help if confined to bed, someone to take him/her to the doctor, someone to prepare meals or someone to do daily chores are included.

Note: Higher scores indicate higher levels of tangible support.

Specifications

Value	Condition(s)	Description	Notes
96	DOSSA = 2	Module not selected	NA
99	ADM_PRX = 1	Module not asked - proxy interview	NS
99	(SSAT02 = DK, R, NS) or<br>(SSAT05 = DK, R, NS) or<br>(SSAT12 = DK, R, NS) or<br>(SSAT15 = DK, R, NS)	At least one required question was not answered (don't know, refusal, not stated)	NS
SSAT02 +<br>SSAT05 +<br>SSAT12 +<br>SSAT15	(0 <= SSAT02 <= 4) and<br>(0 <= SSAT05 <= 4) and<br>(0 <= SSAT12 <= 4) and<br>(0 <= SSAT15 <= 4)	Score obtained on the tangible support subscale	(min: 0; max: 16)

Reference: Sherbourne, C.D. and A.L. Stewart, "The MOS Support Survey" (Medical Outcomes Study Social Support Survey), Social Sciences & Medicine; 32: 705 - 714

2) Affection - MOS Subscale

Variable name: SSADAFF

Based on: SSA_06, SSA_10, SSA_20

Description: This variable measures the level of affection the respondent received. Questions about whether or not the respondent has someone that shows him/her love, someone to hug or someone to love and someone to make him/her feel wanted are included.

Note: Higher scores indicate higher level of affection support.

Specifications

Value	Condition(s)	Description	Notes
96	DOSSA = 2	Module not selected	NA
99	ADM_PRX = 1	Module not asked - proxy interview	NS
99	(SSAT06 = DK, R, NS) or<br>(SSAT10 = DK, R, NS) or<br>(SSAT20 = DK, R, NS)	At least one required question was not answered (don't know, refusal, not stated)	NS
SSAT06 +<br>SSAT10 +<br>SSAT20	(0 <= SSAT06 <= 4) and<br>(0 <= SSAT10 <= 4) and<br>(0 <= SSAT20 <= 4)	Score obtained on the affection support subscale	(min: 0; max: 12)

Reference: Sherbourne, C.D. and A.L. Stewart, "The MOS Support Survey" (Medical Outcomes Study Social Support Survey), Social Sciences & Medicine; 32: 705 - 714

3) Positive Social Interaction - MOS Subscale

Variable name: SSADSOC

Based on: SSA_07, SSA_11, SSA_14, SSA_18

Description: This variable measures the level of positive social interaction the respondent is involved in. Questions about whether the respondent has someone to have a good time with, get together with for relaxation, do things with to get his/her mind off things, or someone to do something enjoyable with are included.

Note: Higher scores indicate higher level of positive social interaction.

Specifications

Value	Condition(s)	Description	Notes
96	DOSSA = 2	Module not selected	NA
99	ADM_PRX = 1	Module not asked - proxy interview	NS
99	(SSAT07 = DK, R, NS) or<br>(SSAT11 = DK, R, NS) or<br>(SSAT14 = DK, R, NS) or<br>(SSAT18 = DK, R, NS)	At least one required question was not answered (don't know, refusal, not stated)	NS
SSAT07 +<br>SSAT11 +<br>SSAT14 +<br>SSAT18	(0 <= SSAT07 <= 4) and<br>(0 <= SSAT11 <= 4) and<br>(0 <= SSAT14 <= 4) and<br>(0 <= SSAT18 <= 4)	Score obtained on the positive social interaction subscale	(min: 0; max: 16)

Reference: Sherbourne, C.D. and A.L. Stewart, "The MOS Support Survey" (Medical Outcomes Study Social Support Survey), Social Sciences & Medicine; 32: 705 - 714

4) Emotional or Informational Support - MOS Subscale

Variable name: SSADEMO

Based on: SSA_03, SSA_04, SSA_08, SSA_09, SSA_13, SSA_16, SSA_17, SSA_19

Description: This variable measures the level of emotional or informational support received by the respondent. Questions about whether the respondent has someone to listen and to advise in a crisis, someone to give information and confide in and talk to, or someone to understand problems are included.

Note: Higher values indicate more emotional or informational support.

Specifications

Value	Condition(s)	Description	Notes
96	DOSSA = 2	Module not selected	NA
99	ADM_PRX = 1	Module not asked - proxy interview	NS
99	(SSAT03 = DK, R, NS) or<br>(SSAT04 = DK, R, NS) or<br>(SSAT08 = DK, R, NS) or<br>(SSAT09 = DK, R, NS) or<br>(SSAT13 = DK, R, NS) or<br>(SSAT16 = DK, R, NS) or<br>(SSAT17 = DK, R, NS) or<br>(SSAT19 = DK, R, NS)	At least one required question was not answered (don't know, refusal, not stated)	NS

SSAT03 +<br>SSAT04 +<br>SSAT08 +<br>SSAT09 +<br>SSAT13 +<br>SSAT16 +<br>SSAT17 +<br>SSAT19	(0 <= SSAT03 <= 4) and<br>(0 <= SSAT04 <= 4) and<br>(0 <= SSAT08 <= 4) and<br>(0 <= SSAT09 <= 4) and<br>(0 <= SSAT13 <= 4) and<br>(0 <= SSAT16 <= 4) and<br>(0 <= SSAT17 <= 4) and<br>(0 <= SSAT19 <= 4)	Score obtained on the emotional / informal support subscale	(min: 0; max: 32)

Reference: Sherbourne, C.D. and A.L. Stewart, "The MOS Support Survey" (Medical Outcomes Study Social Support Survey), Social Sciences & Medicine; 32: 705 - 714

Note finale : Sherbourne, C.D. and A.L. Stewart, "The MOS Support Survey" (Medical Outcomes Study Social Support Survey), Social Sciences & Medicine; 32: 705 - 714

Use of protective equipment (3 DVs)

1) Wears Protective Equipment when In-Line Skating

Variable name: UPEFILS

Based on: UPE_02, UPE_02A, UPE_02B, UPE_02C, UPE_02D

Description: This variable indicates whether the respondent wears a helmet, wrist guards or elbow pads always or most of the time when in-line skating.

Note: Respondents that do not in-line skate were excluded from the population.

Specifications

Value	Condition(s)	Description	Notes
6	DOUPE = 2	Module not selected	NA
9	ADM_PRX = 1	Module not asked - proxy interview	NS
6	UPE_02 = 2	Population exclusions	NA
1	(UPE_02A = 1, 2) and<br>(UPE_02B = 1, 2) and<br>(UPE_02C = 1, 2) and (UPE_02D = 1, 2)	Wears a helmet, wrist guards, elbow pads and knee pads always or most of the time	
2	(UPE_02A = 3, 4) or<br>(UPE_02B = 3, 4) or<br>(UPE_02C = 3, 4) or<br>(UPE_02D = 3, 4)	Does not wear a helmet, wrist guards, elbow pads or knee pads always or most of the time	
9	(UPE_02A = DK, R, NS) or<br>(UPE_02B = DK, R, NS) or<br>(UPE_02C = DK, R, NS) or<br>(UPE_02D = DK, R, NS) or<br>(PAC_1I = DK, R, NS)	At least one required question was not answered (don't know, refusal, not stated)	NS

2) Wears Protective Equipment when Skateboarding

Variable name: UPEFSKB

Based on: UPE_06A, UPE_06B, UPE_06C

Description: This variable indicates whether respondents aged 12 to 19 years old wear a helmet, wrist guards or elbow pads always or most of the time when skateboarding.

Note: Respondents more than 19 years old and respondents that have not skateboarded in the past 12 months were excluded from the population.

Specifications

Value	Condition(s)	Description	Notes
6	DOUPE = 2	Module not selected	NA
9	ADM_PRX = 1 and 12 <= DHH_AGE <= 19	Module not asked - proxy interview	NS
6	DHH_AGE > 19 or UPE_06 = 2	Population exclusions	NA
1	(UPE_06A = 1, 2) and<br>(UPE_06B = 1, 2) and<br>(UPE_06C = 1, 2)	Wears a helmet, wrist guards and elbow pads always or most of the time	

Value	Condition(s)	Description	Notes
2	(UPE_06A = 3, 4) or<br>(UPE_06B = 3, 4) or<br>(UPE_06C = 3, 4)	Does not wear a helmet, wrist guards or elbow pads always or most of the time	
9	(UPE_06A = DK, R, NS) or<br>(UPE_06B = DK, R, NS) or<br>(UPE_06C = DK, R, NS)	At least one required question was not answered (don't know, refusal, not stated)	NS

3) Wears Protective Equipment when Snowboarding

Variable name: UPEFSNB

Based on: UPE_05A, UPE_05B

Description: This variable indicates whether the respondent wears a helmet or wrist guards always or most of the time when snowboarding.

Note: Respondents that have not snowboarded in past 12 months were excluded from the population.

Specifications

Value	Condition(s)	Description	Notes
6	DOUPE = 2	Module not selected	NA
9	ADM_PRX = 1	Module not asked - proxy interview	NS
6	(UPE_03A = 1) or<br>(UPE_03B = 1, 4)	Population exclusions	NA
1	(UPE_05A = 1, 2) and<br>(UPE_05B = 1, 2)	Wears a helmet and wrist guards always or most of the time	
2	(UPE_05A = 3, 4) or<br>(UPE_05B = 3, 4)	Does not wear a helmet or wrist guards always or most of the time	
9	(UPE_05A = DK, R, NS) or<br>(UPE_05B = DK, R, NS)	At least one required question was not answered (don't know, refusal, not stated)	NS

Waiting times (9 DVs)

1) Number of Waiting Days to See a Medical Specialist - Seen Specialist

Variable name: WTMDSO

Based on: WTM_07A, WTM_07B

Description: This variable indicates the number of days that passed between the moment the respondent and his or her doctor decided that the respondent should see a medical specialist and when the actual visit with the specialist took place.

Note: For this variable, the number of waiting days has only been considered for respondents 15 years and older who consulted a medical specialist due to a new health related problem during the past 12 months.

Specifications

Value	Condition(s)	Description	Notes
9996	DOWTM= 2	Module not selected	NA
9996	DHH_AGE < 15 or<br>ACC_10 = 2 or<br>WTM_01 = 2 or<br>WTM_04 = 2	Population exclusions	NA
9999	ADM_PRX = 1	Module not asked - proxy interview	NS
9999	(WTM_07A = DK, R, NS)	Required question was not answered (don’t know, refusal, not stated)	NS
WTM_07A	WTM_07B = 1	Number of waiting days	
WTM_07A * 7	WTM_07B = 2	Number of waiting days	
WTM_07A * 30	WTM_07B = 3	Number of waiting days	

2) Number of Waiting Days to See a Medical Specialist - Not Seen Specialist

Variable name: WTMDSN

Based on: WTM_08A, WTM_08B

Description: This variable indicates the number of days that passed between the moment the respondent and his or her doctor decided the respondent should see a specialist and when the interview took place.

Note: For this variable, the number of waiting days has only been considered for respondents 15 years and older who were referred to a specialist due to a new health related problem during the past 12 months, but who did not see the specialist with whom they had an appointment.

Specifications

Value	Condition(s)	Description	Notes
9996	DOWTM= 2	Module not selected	NA
9996	DHH_AGE < 15 or<br>ACC_10 = 2 or<br>WTM_01 = 2 or<br>WTM_04 = 1	Population exclusions	NA
9999	ADM_PRX = 1	Module not asked - proxy interview	NS
9999	(WTM_08A = DK, R, NS)	Required question was not answered (don’t know, refusal, not stated)	NS
WTM_08A	WTM_08B = 1	Number of waiting days	

WTM_08A * 7	WTM_08B = 2	Number of waiting days	
WTM_08A * 30	WTM_08B = 3	Number of waiting days	

3) Number of Acceptable Waiting Days to See a Medical Specialist

Variable name: WTMDSA

Based on: WTM_07A, WTM_08A, WTM_10, WTM_11A, WTM_11B, WTMDSO, WTMDSN

Description: This variable indicates the number of days, in the respondent's view, he or she can wait to see a medical specialist and still find it acceptable.

Note: The number of acceptable waiting days has only been considered for respondents 15 years and older who were referred to a medical specialist due to a new health related problem during the past 12 months, whether or not they saw the specialist at the time of the interview.

Specifications

Value	Condition(s)	Description	Notes
9996	DOWTM= 2	Module not selected	NA
9996	DHH_AGE < 15 or<br>ACC_10 = 2 or<br>WTM_01 = 2	Population exclusions	NA
9999	ADM_PRX = 1	Module not asked - proxy interview	NS
9999	([WTM_07A = DK, R, NS] and<br>WTM_10 = 1) or<br>([WTM_08A = DK, R, NS] and<br>WTM_10 = 1) or<br>(WTM_11A = DK, R, NS)	At least one required question was not answered (don't know, refusal, not stated)	NS
WTMDSO	WTM_07A < 996 and<br>WTM_10 = 1	Number of acceptable waiting days	
WTMDSN	WTM_08A < 996 and<br>WTM_10 = 1	Number of acceptable waiting days	
WTM_11A	WTM_11B = 1	Number of acceptable waiting days	
WTM_11A * 7	WTM_11B = 2	Number of acceptable waiting days	
WTM_11A * 30	WTM_11B = 3	Number of acceptable waiting days	

4) Number of Waiting Days to Receive Non-Emergency Surgery - Surgery Done

Variable name: WTMDCO

Based on: WTM_21A, WTM_21B

Description: This variable indicates the number of days that passed between the moment the respondent and his or her doctor decided the respondent should receive non-emergency surgery and when the surgery actually took place.

Note: For this variable, the number of waiting days was only considered for respondents 15 years and older who received non-emergency surgery during the past 12 months.

Specifications

Value	Condition(s)	Description	Notes
9996	DOWTM= 2	Module not selected	NA

9996	DHH_AGE < 15 or<br>ACC_20 = 2 or<br>WTM_17 = 2	Population exclusions	NA
9999	ADM_PRX = 1	Module not asked - proxy interview	NS
9999	(WTM_21A = DK, R, NS)	Required question was not answered (don’t know, refusal, not stated)	NS
WTM_21A	WTM_21B = 1	Number of waiting days	
WTM_21A * 7	WTM_21B = 2	Number of waiting days	
WTM_21A * 30	WTM_21B = 3	Number of waiting days	

5) Number of Waiting Days to Receive Non-Emergency Surgery - Surgery Not Done

Variable name: WTMDCN

Based on: WTM_23A, WTM_23B

Description: This variable indicates the number of days that passed between the moment the respondent and his or her doctor decided the respondent should receive non-emergency surgery and when the interview took place.

Note: For this variable, the number of waiting days was only considered for respondents 15 years and older who were referred for non-emergency surgery during the past 12 months, but who did not receive the needed surgery at the time of the interview.

Specifications

Value	Condition(s)	Description	Notes
9996	DOWTM= 2	Module not selected	NA
9996	DHH_AGE < 15 or<br>ACC_20 = 2 or<br>WTM_17 = 1	Population exclusions	NA
9999	ADM_PRX = 1	Module not asked - proxy interview	NS
9999	(WTM_23A = DK, R, NS)	Required question was not answered (don't know, refusal, not stated)	NS
WTM_23A	WTM_23B = 1	Number of waiting days	
WTM_23A * 7	WTM_23B = 2	Number of waiting days	
WTM_23A * 30	WTM_23B = 3	Number of waiting days	

6) Number of Acceptable Waiting Days to Receive Non-Emergency Surgery

Variable name: WTMDCA

Based on: WTM_21A, WTM_23A, WTM_24, WTM_25A, WTM_25B, WTMDCO, WTMDCN

Description: This variable indicates the number of days, in the respondent’s view, he or she can wait to receive a non-emergency surgery and still find it acceptable.

Note: The number of acceptable waiting days was only considered for respondents 15 years and older who were referred to receive non-emergency surgery during the past 12 months, whether the respondent received his surgery or not at the time of the interview.

Specifications

Value	Condition(s)	Description	Notes
9996	DOWTM= 2	Module not selected	NA

9996	DHH_AGE < 15 or<br>ACC_20 = 2	Population exclusions	NA
9999	ADM_PRX = 1	Module not asked - proxy interview	NS
9999	([WTM_21A = DK, R, NS] and<br>WTM_24 = 1) or<br>([WTM_23A = DK, R, NS] and<br>WTM_24 = 1) or<br>(WTM_25A = DK, R, NS) or (WTM_25A < 996 and<br>WTM_25B = 9)	At least one required question was not answered (don't know, refusal, not stated)	NS
WTMDCO	WTM_21A < 996 and<br>WTM_24 = 1	Number of acceptable waiting days	
WTMDCN	WTM_23A < 996 and<br>WTM_24 = 1	Number of acceptable waiting days	
WTM_25A	WTM_25B = 1	Number of acceptable waiting days	
WTM_25A * 7	WTM_25B = 2	Number of acceptable waiting days	
WTM_25A * 30	WTM_25B = 3	Number of acceptable waiting days	

7) Number of Waiting Days for Diagnostic Test - Test Done

Variable name: WTMDTO

Based on: WTM_38A, WTM_38B

Description: This variable indicates the number of days that passed between the moment the respondent and his or her doctor decided the respondent should receive a magnetic resonance imaging test (MRI), a Computed Tomography exam (CT-SCAN) or a non-emergency angiography (heart test) and when the test was actually received.

Note: For this variable, the number of waiting days was only considered for respondents of 15 years and older who received a MRI or a CT-SCAN exam, or a non-emergency heart test during the past 12 months.

Specifications

Value	Condition(s)	Description	Notes
9996	DOWTM= 2	Module not selected	NA
9996	DHH_AGE < 15 or<br>ACC_30 = 2 or<br>WTM_32 = 2	Population exclusions	NA
9999	ADM_PRX = 1	Module not asked - proxy interview	NS
9999	(WTM_38A = DK, R, NS)	Required question was not answered (don't know, refusal, not stated)	NS
WTM_38A	WTM_38B = 1	Number of waiting days	
WTM_38A * 7	WTM_38B = 2	Number of waiting days	
WTM_38A * 30	WTM_38B = 3	Number of waiting days	

8) Number of Waiting Days for Diagnostic Test - Test Not Done

Variable name: WTMDTN

Based on: WTM_39A, WTM_39B

Description: This variable indicates the number of days that passed between the moment the respondent and his or her doctor decided the respondent should receive a magnetic resonance imaging test (MRI), a Computed Tomography exam (CT-SCAN) or a non-

emergency angiography (heart test) and when the interview took place.

Note: For this variable, the number of waiting days was only considered for respondents 15 years and older who were referred to receive a MRI or a CT-SCAN exam, or a non-emergency heart test during the past 12 months, but who had not received the test at the time of the interview.

Specifications

Value	Condition(s)	Description	Notes
9996	DOWTM= 2	Module not selected	NA
9996	DHH_AGE < 15 or<br>ACC_30 = 2 or<br>WTM_32 = 1	Population exclusions	NA
9999	ADM_PRX = 1	Module not asked - proxy interview	NS
9999	(WTM_39A = DK, R, NS)	Required question was not answered (don't know, refusal, not stated)	NS
WTM_39A	WTM_39B = 1	Number of waiting days	
WTM_39A * 7	WTM_39B = 2	Number of waiting days	
WTM_39A * 30	WTM_39B = 3	Number of waiting days	

9) Number of Acceptable Waiting Days for Diagnostic Test

Variable name: WTMDTA

Based on: WTM_38A, WTM_39A, WTM_40, WTM_41A, WTM_41B, WTMDTO, WTMDTN

Description: This variable indicates the number of days, in the respondent's view, he or she can wait to receive a magnetic resonance imaging test (MRI), a Computed Tomography exam (CT-SCAN) or a non-emergency angiography (heart test) and still find it acceptable.

Note: The number of acceptable waiting days was only considered for respondents 15 years and older who were referred to pass a MRI or a CT-SCAN exam, or a non- emergency heart test during the past 12 months, whether the respondent received the test or not at the time of the interview.

Specifications

Value	Condition(s)	Description	Notes
9996	DOWTM= 2	Module not selected	NA
9996	DHH_AGE < 15 or<br>ACC_30 = 2	Population exclusions	NA
9999	ADM_PRX = 1	Module not asked - proxy interview	NS
9999	([WTM_38A = DK, R, NS] and<br>WTM_40 = 1) or<br>([WTM_39A = DK, R, NS] and<br>WTM_40 = 1) or<br>(WTM_41A = DK, R, NS)	At least one required question was not answered (don't know, refusal, not stated)	NS
WTMDTO	WTM_38A < 996 and<br>WTM_40 = 1	Number of acceptable waiting days	
WTMDTN	WTM_39A < 996 and<br>WTM_40 = 1	Number of acceptable waiting days	
WTM_41A	WTM_41B = 1	Number of acceptable waiting days	
WTM_41A * 7	WTM_41B = 2	Number of acceptable waiting days	
WTM_41A * 30	WTM_41B = 3	Number of acceptable waiting days	
